# Social and Structural Determinants of Urban American Indian and Alaska Native Health: A Case Study in Los Angeles

**DOI:** 10.15766/mep_2374-8265.10825

**Published:** 2019-05-15

**Authors:** Andrea N. Garcia, Monique C. Castro, John Paul Sánchez

**Affiliations:** 1 Visiting Project Scientist, Center for Health Services and Society, Department of Psychiatry, UCLA David Geffen School of Medicine; 2 Physician Specialist, Los Angeles County Department of Mental Health; 3 Board Member, BNGAP, Inc.; 4 Owner, Indigenous Circle of Wellness; 5 Founder, Indigenous Circle of Wellness; 6 Associate Professor, Emergency Medicine, Rutgers New Jersey Medical School; 7 Associate Dean, Diversity and Inclusion, Rutgers New Jersey Medical School; 8 Founder, BNGAP, Inc.

**Keywords:** Social Determinants of Health, Population Health, Cultural Competence, Health Policy, Health Equity, Diversity and Inclusion, Urban, Community-Based Medicine, Editor's Chioce, American Indian and Alaska Native, Structural Determinants, Historical Trauma, Alaska Natives, Intergenerational Trauma

## Abstract

**Introduction:**

American Indians and Alaska Natives (AIAN) experience significant health inequities, yet there are very few curricula dedicated to training a culturally sensitive workforce to care for this population. There is a further dearth of curricula that center on Indigenous values and ways of knowing.

**Methods:**

We developed a 90-minute interactive workshop aimed at increasing faculty and trainee understanding of the social and structural determinants of urban AIAN health. The workshop consisted of a PowerPoint presentation, two videos, an interactive storytelling exercise, and reflection exercises. Participants also completed pre-/postworkshop questionnaires. The workshop was implemented three times at two medical schools.

**Results:**

There were a total of 35 diverse participants. Regarding the effect of the workshop on participants’ knowledge base, a comparison of pre- and postworkshop questionnaire responses showed a statistically significant (*p* < .05) increase in the correct answer being chosen for each question. All participants agreed or strongly agreed that each of the three learning objectives had been met. Participants particularly valued the workshop's interactive nature, as well as its use of storytelling and multimedia to reinforce policy impact.

**Discussion:**

This workshop provided an interactive and effective method to increase participant knowledge of the importance of a land acknowledgment, of connecting federal Indian policy to health outcomes, and of how AIAN identity may impact access to health care.

## Educational Objectives

By the end of this activity, learners will be able to:

1. Describe the importance of and how to identify the tribal homelands on which they stand.
2. Describe how at least two federal Indian policies have impacted the demographics and health outcomes of American Indians and Alaska Natives (AIAN) in urban areas.
3. Explain how AIAN identity may be associated with access to health care.

## Introduction

Urban-dwelling American Indians and Alaska Natives (AIAN) experience disproportionate mortality secondary to heart disease, cancer, diabetes, unintentional injuries, and chronic liver disease.^1^ Access to health care is thought to explain some of the marked health disparities between AIAN and other racial groups, while larger social and structural determinants of health are the primary drivers behind these disparities. In regard to access, urban-dwelling AIAN also face unique and complex challenges. The Indian Health Service (IHS) is the federal agency responsible for providing health care to 2.3 million AIAN.^2^ However, IHS is not health insurance, cannot provide minimum essential health benefits, and sometimes requires being enrolled in a tribe.^3,4^ Urban AIAN face additional barriers since urban Indian health organizations (UIHOs) funded by IHS receive only one percent of the entire IHS budget, despite 71% of AIAN residing in urban areas.^5,6^ In Los Angeles County, for instance, which boasts the largest population of AIAN nationwide at 171,163, only one culturally sensitive UIHO exists. AIAN can access health systems outside of the IHS, but there remains a lack of literature on AIAN preferences for culturally specific health care and even utilization of IHS, insurance, or both.

Additionally, there is a dearth of AIAN health professionals and an arguably inadequate culturally sensitive health workforce knowledgeable about the unique determinants of health among this population.^7,8^ Approximately 43% of MD-granting institutions in 2016–2017 had no (zero) students who identified as AIAN (alone).^8^ A recent report by the Association of American Medical Colleges (AAMC) and the Association of American Indian Physicians (AAIP) outlines recommendations to boost AIAN representation in medicine.^8^ While this long-term strategy is exceedingly important, a shorter-term strategy of training a culturally sensitive workforce remains of practical and pressing significance. There are no known data on how many medical schools teach specifically about urban AIAN issues in health care, let alone the larger structural issues that have influenced the health of this population. To this last point, other AIAN groups and initiatives find it exceedingly important to shift the narrative from an “individual deficits and contradictory stereotypes” lens to a framework that centers on Indigenous values, history, and contemporary portrayals of Indigenous people.^9,10^ The University of Minnesota, Duluth, successfully Indigenized the process of medical school curriculum development and implemented a 7-hour region-specific course for all of its first-year medical school trainees.^11^ Our workshop is intimately aligned with the values of these frameworks.

*MedEdPORTAL* has one excellent AIAN-specific curriculum that provides a comprehensive health systems overview, and it uses a rural South Dakota community as a case study.^12^ However, the reality is that urban-dwelling AIAN represent approximately 71% of the AIAN population, and most medical schools are in urban areas. Our workshop contextualizes urban AIAN disparities through centering Indigenous values and history as noted above. Of note, two of the coauthors of this module are urban-dwelling AIAN and have extensive experience in AIAN health systems research, clinical care, and development and implementation of AIAN-specific curricula for various audiences. We draw on our shared cultural values of storytelling to reinforce the workshop's content and provide multimedia tools to address different learning styles. The multimedia tools are also intentionally used to provide a contemporary portrayal of Indigenous people.

The six-step Kern model was used as a tool for creating the structure, implementation, and assessment of the workshop.^13^ In step 1, our problem identification and general needs assessment consisted of performing a literature review. For step 2, we relied on the recent AAMC/AAIP report, *Reshaping the Journey: American Indians and Alaska Natives in Medicine,*^8^ to act as a needs assessment in the learning environment. In step 3, we determined our goals and objectives based on the literature review and coauthor collaboration. In step 4, our chosen educational strategies included a multimedia and interactive PowerPoint (PPT) presentation, storytelling exercise, and videos. Each component incorporated reflection, understanding, and sharing of perspectives via large-group reflection. In step 5, workshop implementation was via special Native American Heritage Month health presentations for medical students, residents, and physicians at four different medical schools in urban New York and New Jersey. In step 6, evaluation and feedback, a questionnaire was developed for each participant to use to evaluate the design and content of the workshop. However, the Institutional Review Board (IRB) application covered just two of the four sites (CUNY School of Medicine [CUNY] and Rutgers New Jersey Medical School [RNJMS]), and so, the Results section reflects only participants from these two sites.

This workshop addresses core concepts such as understanding the importance of doing a land acknowledgment, describing how at least two federal policies have impacted the demographics and health of urban AIAN, and describing how AIAN identity may impact access to health care. For facilitators who may not be familiar, a land acknowledgment is a formal acknowledgment of the original stewards of the land and their connection to it. It is important to do a land acknowledgment as a sign of respect and a step towards healing, truth, and reconciliation for Indigenous communities and beyond. One does not need to be Indigenous to do a land acknowledgment. This module was tailored for medical students, residents, and physicians but can also be appropriate for other health care professionals or trainees. This workshop provides an engaging and interactive curriculum that is rooted in Indigenous knowledge and ways of knowing, is unique to urban Indian health, and adds an important contribution to the currently few targeted curricula on AIAN.

## Methods

The workshop was drafted and revised by a team of experts in AIAN health, AIAN research, educational scholarship, and/or academic medicine. The primary team consisted of three individuals: Monique C. Castro, an AIAN licensed psychotherapist, consultant, trainer, educator, and activist; Andrea N. Garcia, an AIAN physician, public health practitioner, and researcher within an academic institution; and John Paul Sánchez, an associate dean for diversity and inclusion with prior research experience on AIAN representation in the academic medicine workforce. The workshop was cofacilitated by coauthors Andrea N. Garcia and Monique C. Castro. It was implemented five times at four different medical schools in the states of New York (once each at New York Medical College, CUNY, and Weill Cornell Medical College) and New Jersey (twice at RNJMS). We were able to attain IRB approval for only two of the four sites (CUNY and RNJMS), and so, our results include participants from two workshops at RNJMS and one at CUNY. The reason why this Los Angeles–based curriculum was implemented in New York/New Jersey was that each of the above institutions hosted Native American Heritage Month events in November 2018 and invited speakers to present.

The presentation was developed to increase understanding of urban AIAN social and structural determinants of health. The workshop was targeted to medical students, residents, and physicians but can also be appropriate for other health care professionals or trainees. The preferred facilitator would be a fellow or faculty member with experience in diversity and inclusion programming and/or a robust understanding of the social and structural determinants of health and/or specific understanding of AIAN health and health systems. The workshop featured three primary educational strategies: (1) an interactive didactic component via PPT presentation, (2) a large-group interactive storytelling exercise for trainees to reflect on potential policies and social determinants that may have impacted the health of their assigned characters, and (3) the use of technology and art, in this case, two videos, that employed visual and auditory methods to reinforce the primary learning objectives, as well as an unofficial objective of depicting contemporary AIAN people.

The PPT (Appendix A) started with the facilitators doing a land acknowledgment, followed by a video that contextualized the importance of doing so, and concluded with a resource describing how to perform a land acknowledgment. These slides were followed by a second video. Before the start of the video, participants were asked to keep in mind certain questions that were meant to prime the discussion on social and structural determinants of health. At the conclusion of the video, participants were asked to reflect on and discuss those questions. This was followed by reinforcing and defining terms such as *social and structural determinants of health, Indigenous,* and *colonization.*

These activities were then followed by the experiential storytelling exercise outlined in detail in the facilitator guide (Appendix B). In short, the participants received specific cards distributed at random and were asked to read their cards at the designated time. Participants were asked to keep in mind certain questions at the beginning of the exercise; these questions were discussed as a large group at the end of the exercise.

The remainder of the presentation was more didactic and meant to reinforce policies explored in the storytelling exercise in a linear and visual fashion. A brief overview of urban AIAN access to health care and of how AIAN identity can impact access to health care followed, concluding with individual, institutional, and federal recommendations/best practices for caring for AIAN populations.

The following is a listing and description of the resources provided to conduct the workshop successfully.

### Appendix A. PowerPoint Presentation

The flow and content of the workshop are featured in this 48-slide PPT presentation. The presentation outlines the core content for the participants, including why and how to do a land acknowledgment, key terms related to the social and structural determinants of health, a review of federal Indian policy, and an overview of urban AIAN access to health care.

### Appendix B. Facilitator Guide

This document outlines step-by-step instructions for conducting the workshop along with an explanation of how to discuss each PPT slide. Slide instructions have been included to ensure consistent implementation of the workshop across presentation sites. Facilitators who do not identify as AIAN or are not knowledgeable about AIAN content are encouraged to adhere to the guide rather strictly, using the examples provided to further reinforce key concepts. This is important both for consistency and also because facilitators need to portray examples that are in alignment with Indigenous values, history, and contemporary portrayals.

### Appendix C. Video Honor Native Land

This video is provided as an appendix to minimize the likelihood of technical difficulties or website changes. The video is to be shown at slide 3.

### Appendix D. Video The Art of Indigenous Resistance

This video is provided as an appendix to minimize the likelihood of technical difficulties or website changes. The video is to be shown at slide 9.

### Appendix E. Evaluation Form

Participants were asked to complete a pre- and postworkshop questionnaire. The primary questions served to assess participants’ knowledge of AIAN content that aligned with the workshop objectives. Secondary questions served to collect demographic information about participants, as well as to gather feedback about the effectiveness of the workshop. Each questionnaire took approximately 5 minutes to complete. The following questions were included in both the pre- and postworkshop questionnaires:

1. A land acknowledgment (circle one response):
  a. Can only be performed by someone of American Indian/Alaskan Native identity.
  b. Recognizes the impact that climate change is having on the land.
  c. Is to acknowledge the original caretakers of the land.
  d. All of the above.
2. Which American Indian/Alaska Native tribe identifies the land that this institution resides on as their original homelands (circle one response)?
  a. Cherokee.
  b. Mohawk.
  c. Lenape.
  d. All of the above.
3. The purpose of the Relocation Act was to do the following (circle one response):
  a. To forcibly move American Indian/Alaska Native tribes to reservation land.
  b. To encourage American Indians/Alaska Natives to gain vocational skills and move to urban areas.
  c. To relocate American Indians/Alaska Natives from their land to make space for the U.S. agricultural industry.
  d. All of the above.
4. Who is eligible for health care through the Indian Health Service?
  a. Self-identified American Indian/Alaska Native individuals.
  b. Federally recognized American Indians/Alaska Natives with proof of tribal enrollment.
  c. Any individual residing on a reservation.
  d. All of the above.

Demographic questions were asked on the preworkshop survey. The postworkshop survey also included questions on how well the workshop objectives had been met. Lastly, the following two open-ended questions were included in the postworkshop survey alone:

• What did you like about this workshop?
• What suggestions do you have to improve this workshop?

Review of the PPT, facilitator guide, and evaluation form and testing of the videos by facilitators take approximately 2 hours. No more than two cofacilitators should lead the workshop. If there is more than one facilitator, then additional time should be spent by phone or face to face to discuss the breakdown of slides between individuals and to run a practice session.

Materials required include pens; audiovisual equipment to show the PPT presentation; computer with video and audio capability; printed story cards on pink, blue, green, or white paper as described in the facilitator guide; and printed copies of the evaluation form. The optimal length of this workshop is 90 minutes; however, it can be tailored based on resources. A suggested time line for the workshop is as follows:

• Preworkshop evaluation: 5 minutes.
• Slides 1–4, land acknowledgment: 10 minutes.
• Slides 5–13, introduction of objectives and video discussion: 15 minutes.
• Slides 14–26, storytelling exercise: 25 minutes.
• Slides 27–47, reinforcing policies, Los Angeles case study, urban Indian health care, and recommendations: 20 minutes.
• Slide 48: questions and answers: 10 minutes.
• Postworkshop evaluation: 5 minutes.

## Results

The 35 workshop respondents were a diverse sample: Twenty-six (74%) identified as female; six (17%) as lesbian, gay, or bisexual; five (14%) as Hispanic/Latino; 15 (43%) as white; six (17%) as African American/black; and nine (26%) as Asian. There were eight medical students, one dental student, two graduate students, one resident, 15 faculty, and eight staff. The number of participants per session ranged from nine to 15.

In terms of the effect of the workshop on participants’ knowledge base, a comparison of pre- and postworkshop questionnaire responses showed a statistically significant increase in the correct answer being chosen (Table). For each question, a paired-samples *t* test showed a statistically significant difference of at least at *p* < .05. All participants agreed or strongly agreed that each of the learning objectives had been met.

**Table.** Percentage of Correct Responses per Question Pre- and Postworkshop

Question and Correct Answer	Mean Correct Responses	
	Pretest	Posttest
1. A land acknowledgment … is to acknowledge the original caretakers of the land.^a^	40.0%	74.3%
2. Which American Indian/Alaska Native tribe identified the land that this institution resides on as their original homelands … Lenape.^b^	45.7%	77.1%
3. The purpose of the Relocation Act was to do the following … to encourage American Indians/Alaska Natives to gain vocational skills and move to urban areas.^a^	2.9%	65.7%
4. Who is eligible for health care through the Indian Health Service … federally recognized American Indians/Alaska Natives with proof of tribal enrollment.^b^	65.7%	85.7%

a *p* < .01.

b *p* < .05.

Organized by learning objective, the following qualitative feedback from participants describes what they liked about the workshop.

• Objective 1: Describe the importance of and how to identify the tribal homelands on which you stand.
  ○ “Starting out with land acknowledgement and lifting up the strength and resilience of native organizing was an excellent way to start. Indigenizing and not just decolonizing shift is powerful.”
• Objective 2: Describe how at least two federal Indian policies have impacted the demographics and health outcomes of AIAN in urban areas.
  ○ “Enjoyed the used of storytelling to educate participants about the historical policies that have impacted AIAN health over many generations.”
  ○ “I liked the integration of policy into personal stories to show the impact that these policies have on Native Americans.”
  ○ “Historical background, via storytelling, provided tangible ways to continue to educate ourselves and move institutions forward.”
  ○ “The interactive storytelling activity was a really nice way of tying together a family's health and social case study in with the changing political movement in the last century.”
• Objective 3: Explain how AIAN identity may be associated with access to health care.
  ○ “I felt like I learned a lot and generated even more questions and feelings on how much I don't know and wish I could learn to be educated and a literate ally.”
  ○ “Gave me a better understanding of AIAN experiences of situation related to environment, health care access, etc.”
  ○ “I liked the focus on the historical impact on health today. Also liked the discussion of childhood/intergenerational trauma impact.”

The participants also commented on how the workshop could be improved:

• “More content on unique health issues and disparities.”
• “Providing more content in terms of AIAN health disparities across a broad range of health areas. Most people are completely unaware of how large many health disparities are for this community.”
• “Concrete ways as providers to better support patients would be helpful.”

Participants enjoyed the workshop because of its use of media (e.g., videos and interactive map exercise), historical perspectives, and storytelling to facilitate an appreciation of how federal policies have impacted AIAN health. Participants also appreciated how the perspectives of various generations were highlighted in the videos (e.g., youth) and in the storytelling exercise (e.g., youth to elders). Because of the inclusion of various perspectives by generation and gender, several learners desired further information by subgroup: for example, “I'd love to learn about AIAN women specific issues and empowerment and social justice work.”

## Discussion

This workshop was created to teach diverse trainees and health professionals about the social and structural determinants of urban AIAN health. The workshop helps to remedy the paucity of material related to AIAN health and in particular fills a knowledge gap in urban AIAN health. Most importantly, the workshop is taught using methods that center on Indigenous values, history, and a contemporary portrayal of AIAN people. Accordingly, the objectives are for participants to learn why and how to do a land acknowledgment, to be able to describe how at least two federal Indian policies have affected AIAN health, and to be able to describe how AIAN identity may affect access to health care. These objectives were overwhelmingly met according to both quantitative and qualitative feedback from participants. Overall, the workshop was well received, and there were statistically significant improvements in participant knowledge. Furthermore, we were able to make improvements to the workshop based on suggestions identified in participant evaluations.

There were several attributes that made this an effective workshop. Foremost, nearly all of the participants felt that the storytelling activity was a powerful way to discuss the intersection of policy and health. Some participants thought storytelling made the content more “personal,” while others said it provided a “tangible” way to learn about history. Second, most participants appreciated the interactive nature of the entire workshop, which included videos and reflection questions in addition to the actual storytelling exercise. Some participants noted that they appreciated the inclusive undertones of the presentation, while others appreciated the intergenerational approach to the material.

There were also opportunities for improvement. Some participants desired more content on the unique and specific health disparities for this population, including implications for mental health outcomes. Others wanted concrete suggestions and best practices for providers to help better care for their AIAN patients, while still others requested a handout of additional resources and a list of some of the key policies mentioned. Additional suggestions were for more time to be spent on federal programs for AIAN and for more content and comparisons to Indigenous health in other countries; two participants wished to learn more about the integration of traditional healing with Western medicine. Generally, participants wished that the workshop was longer and that content like this could be taught more frequently.

We have updated some of the workshop's content in response to participant feedback. Although this is a case study on AIAN in Los Angeles, we have added a couple of slides to better capture the health of urban AIAN nationwide in comparison to other racial/ethnic groups. That being said, learners should still be aware of the unique identities and disparities between the 570+ federally recognized tribes. We still depict Los Angeles AIAN health statistics as a more in-depth case study given that it is the county with the largest population of AIAN in the country. We have also added a link to the Urban Indian Health Institute's data dashboard for the facilitator to demonstrate, in order to simplify retrieval of statistics in other urban areas. Although participants wished for content such as comparison of Indigenous health in other countries or ways to integrate traditional medicine with Western medicine, we feel that these are beyond the scope of this particular presentation. We do believe, however, that such requests are indicative of a need and desire for more AIAN-related content in general.

Despite the robust feedback and subsequent workshop adaptation, there are limitations to our evaluation. For one, because the pre- and postworkshop assessments were administered immediately before and after the workshop, there was no way of tracking participants’ long-term knowledge retention or the workshop's potential impact on their own clinical practice. Second, it is unknown whether participants encounter AIAN patients or, if so, how many—which could indicate a possible limitation in knowledge retention and generalizability. However, we believe that the grounding in Indigenous values and history, as well as the weaving in of social and structural determinants of health, is applicable to all patients. Although our exercise tells the story of one family, we make it a point to acknowledge the diverse histories, stories, and intersections of identity held by all of our patients.

Lastly, we would like to underscore that facilitator choice and workshop size are likely the most important components in ensuring a successful workshop. We acknowledge that some may believe only AIAN individuals can facilitate a workshop on AIAN content. We respectfully disagree for a few reasons. First, the detailed facilitator guide provides a roadmap for non-AIAN to deliver or incorporate components into standing medical school curricula. Second, there are simply not enough AIAN faculty to teach the content. AIAN make up only 0.48% of the total full-time faculty members at MD-granting institutions.^8^ Furthermore, we push back on the assertion that AIAN must teach AIAN content and hold that this expectation increases the burden of the minority tax. The minority tax can be described as the burden of extra responsibilities placed on minority faculty in the name of diversity.^14^ While some AIAN faculty may wish to undertake certain efforts, others may be overburdened with similar responsibilities or perhaps may not be automatic experts on certain aspects of the content. While an AIAN person with knowledge in the content area would certainly be preferred, we also believe that allies with experience in diversity and inclusion and with a strong understanding of social and structural determinants of health could be excellent facilitators. From a sheer numeric perspective, the more inclusive the criteria are for potential facilitators, the greater potential this workshop has to be taught to more trainees and faculty. Otherwise, we highly support the model of community faculty or elders in residence who may not be medically trained faculty but are AIAN/Indigenous community members who are knowledgeable of Indigenous ways of being to teach the content. Lastly, this workshop can be offered to any large class, but we recommend limiting the storytelling portion to smaller groups to ensure a safe place for emotional discussions.

In summary, this workshop addresses a largely unmet need for AIAN content in medical education curricula. Although it is presented from the perspective of urban AIAN, the lens of social and structural determinants, coupled with the tools and themes of storytelling, intersectionality, and intergenerational approaches, is applicable to all patient populations. We believe that the interactive approach and storytelling exercise are a particularly effective technique that could be applied to other aspects of medical education curricula. We further believe that contextualizing health disparities with Indigenous values is an effective educational intervention and facilitates learning about true healing.

## Appendices

A. PowerPoint Presentation.pptx
B. Facilitator Guide.docx
C. Video Honor Native Land.mp4
D. Video The Art of Indigenous Resistance.mp4
E. Evaluation Form.pdf All appendices are peer reviewed as integral parts of the Original Publication.
