# Supplementary material for: Social and Structural Determinants of Urban American Indian and Alaska Native Health: A Case Study in Los Angeles
Source: MedEdPORTAL. 2019 May 15;15:10825. doi: 10.15766/mep_2374-8265.10825 (PMC6543927; doi:10.15766/mep_2374-8265.10825)
Supplement: Supplementary file 1 — A. PowerPoint Presentation.pptx B. Facilitator Guide.docx C. Video Honor Native Land.mp4 D. Video The Art of Indigenous Resistance.mp4 E. Evaluation Form.pdf [file mep-15-10825-s001.zip › A. PowerPoint Presentation.pptx]

## Slide 1
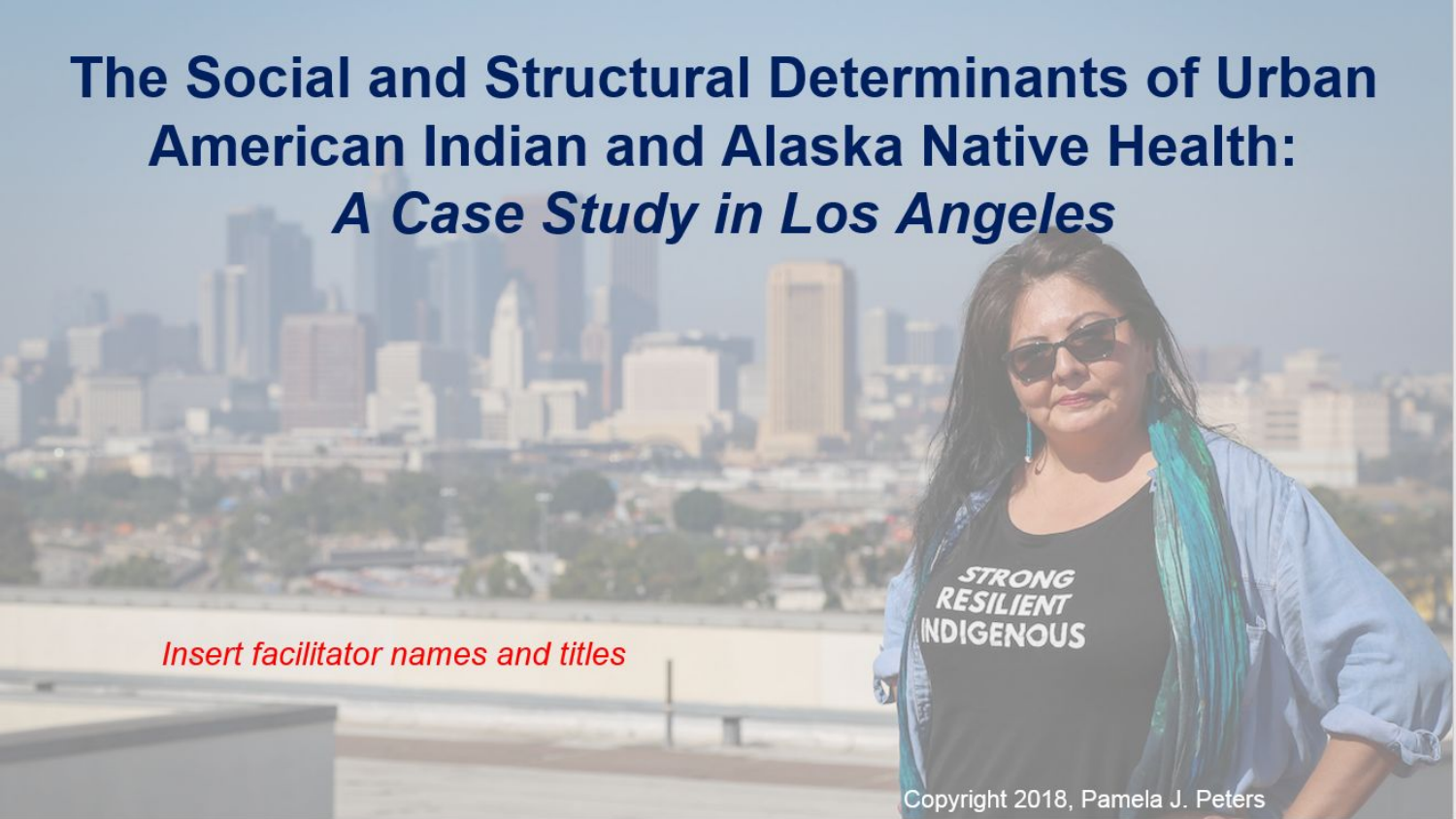

## Slide 2
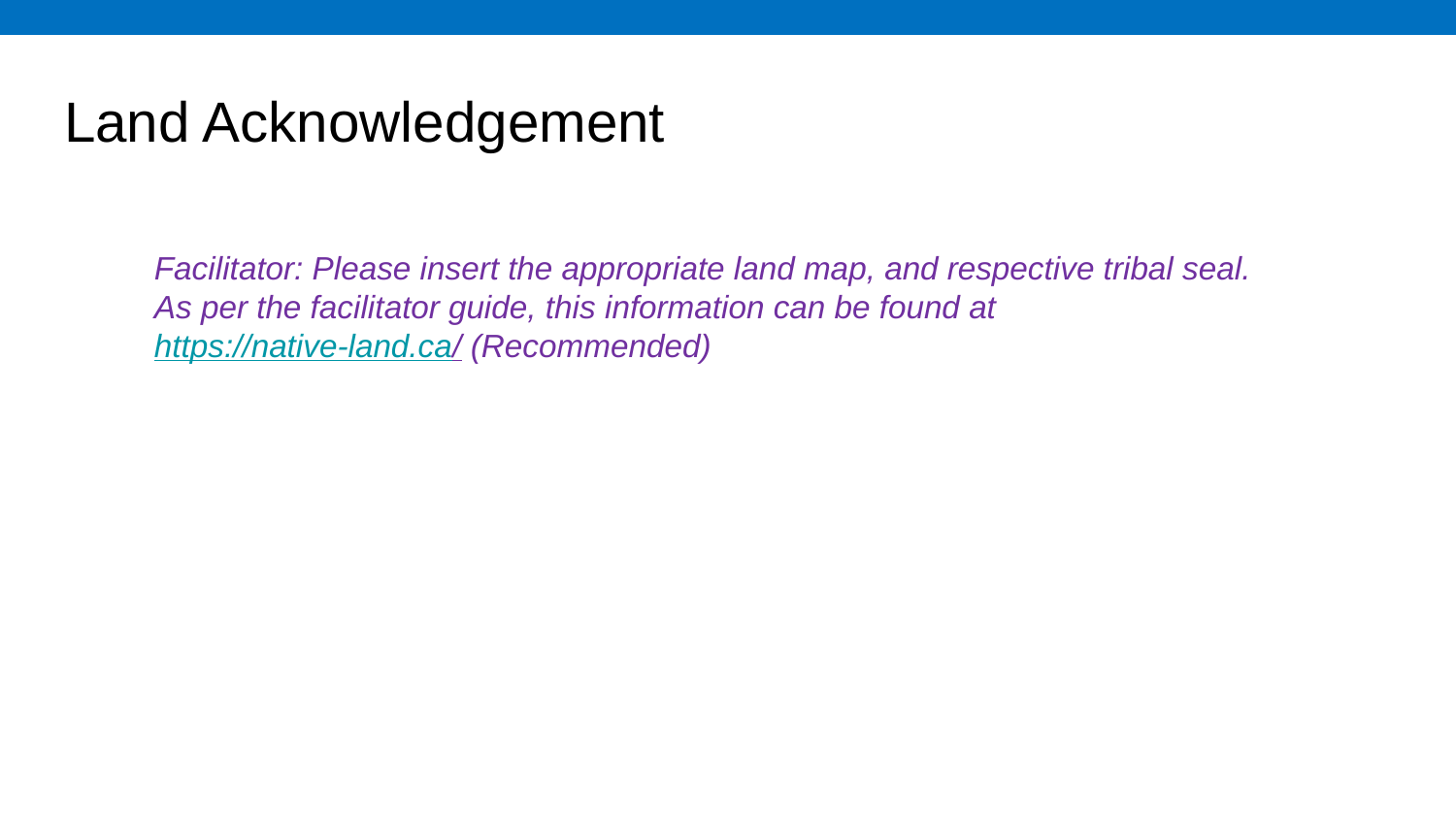

# Land Acknowledgement
Facilitator: Please insert the appropriate land map, and respective tribal seal. As per the facilitator guide, this information can be found at https://native-land.ca/ (Recommended)

## Slide 3
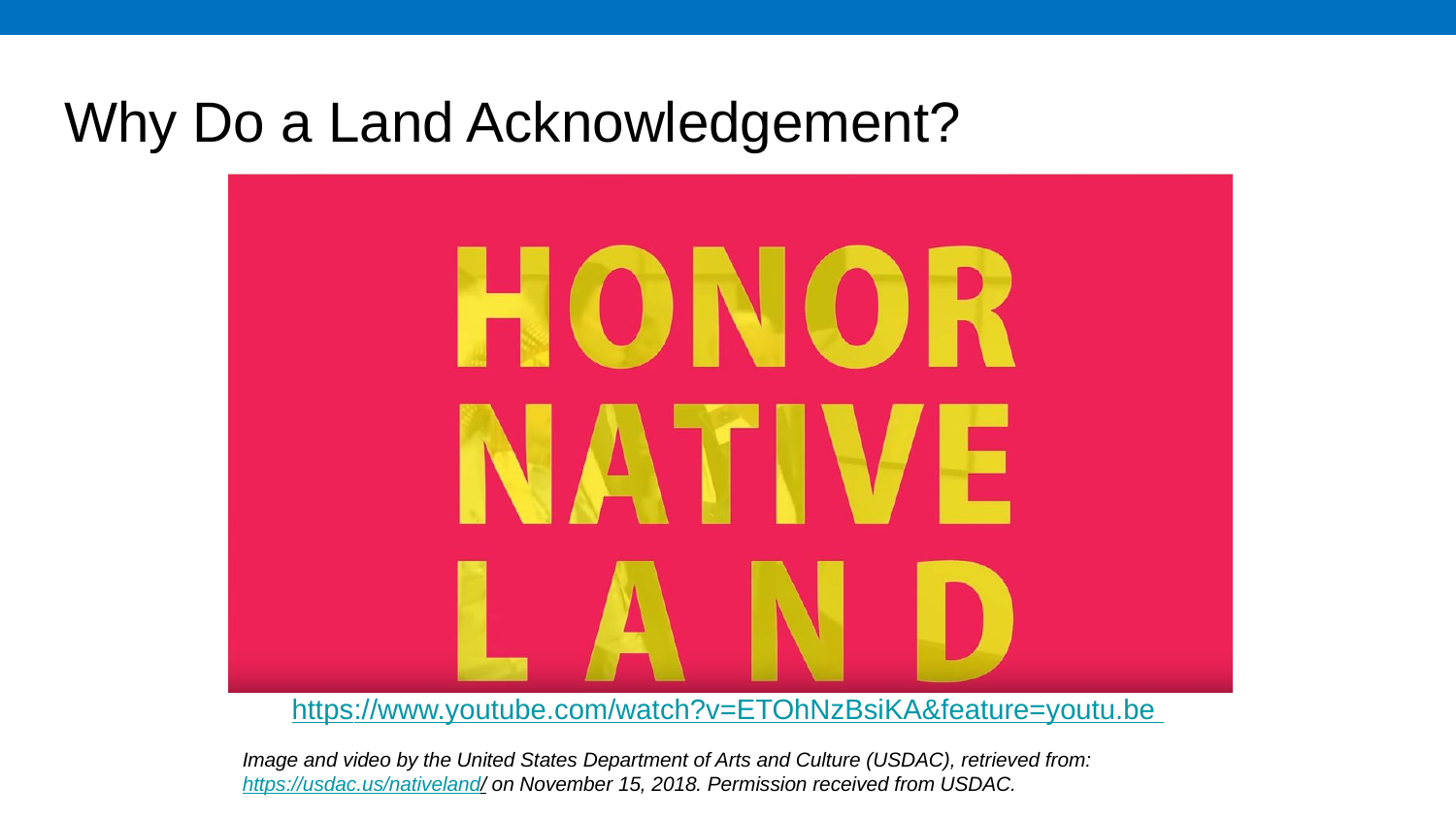

# Why Do a Land Acknowledgement?
https://www.youtube.com/watch?v=ETOhNzBsiKA&feature=youtu.be
Image and video by the United States Department of Arts and Culture (USDAC), retrieved from: https://usdac.us/nativeland/ on November 15, 2018. Permission received from USDAC.

## Slide 4
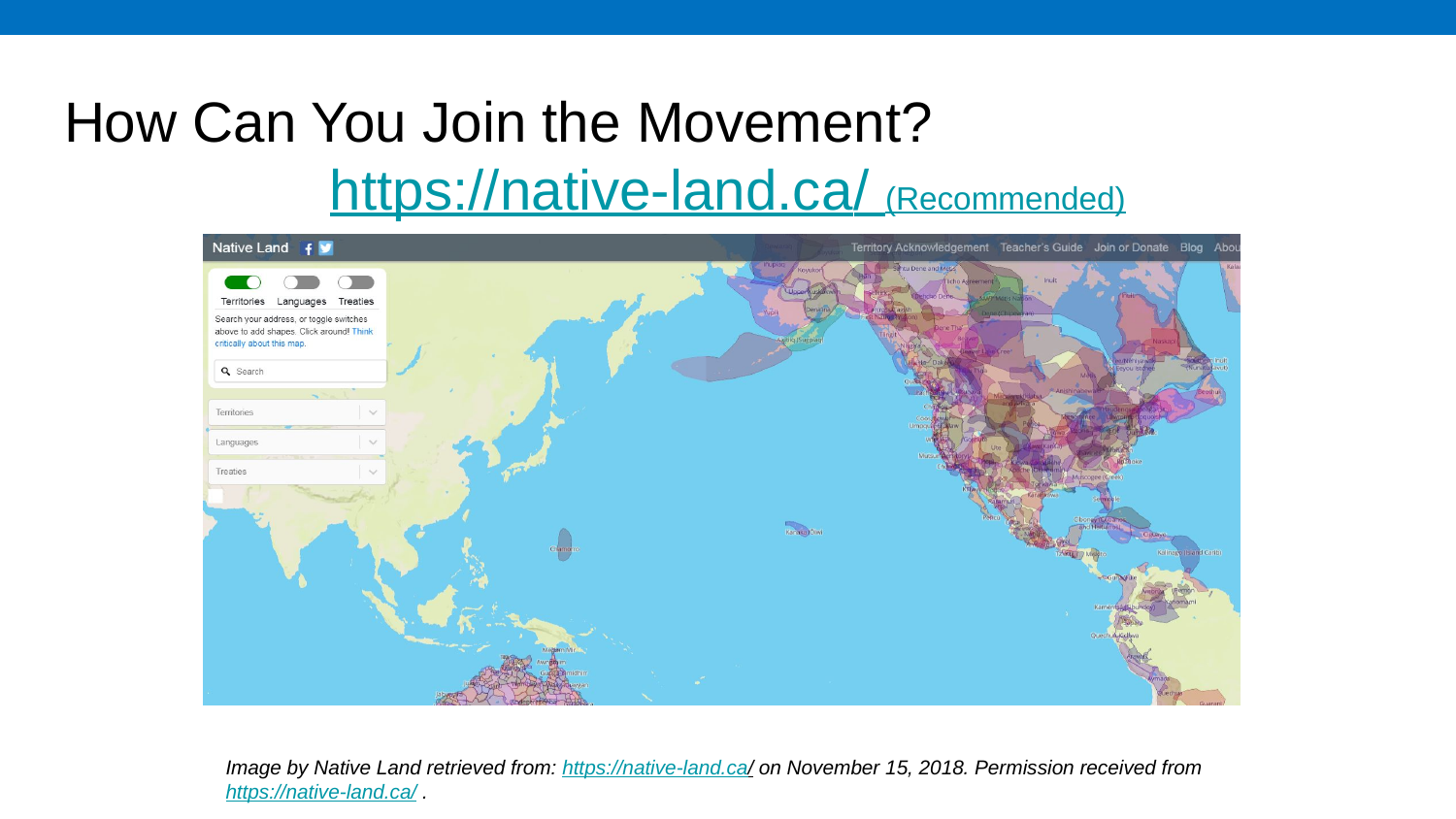

# How Can You Join the Movement?
https://native-land.ca/ (Recommended)
Image by Native Land retrieved from: https://native-land.ca/ on November 15, 2018. Permission received from https://native-land.ca/ .

## Slide 5
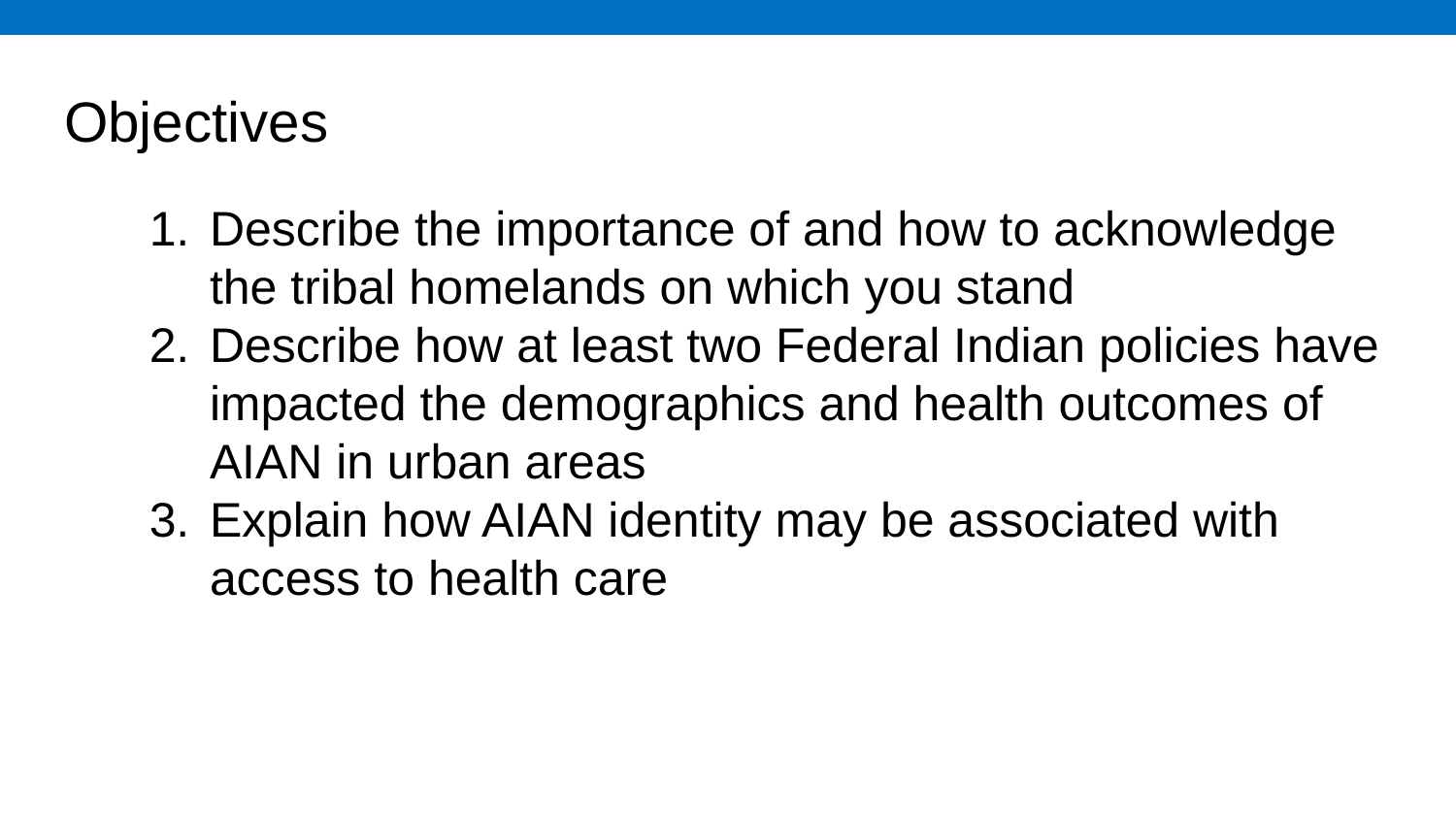

# Objectives
Describe the importance of and how to acknowledge the tribal homelands on which you stand
Describe how at least two Federal Indian policies have impacted the demographics and health outcomes of AIAN in urban areas
Explain how AIAN identity may be associated with access to health care

## Slide 6
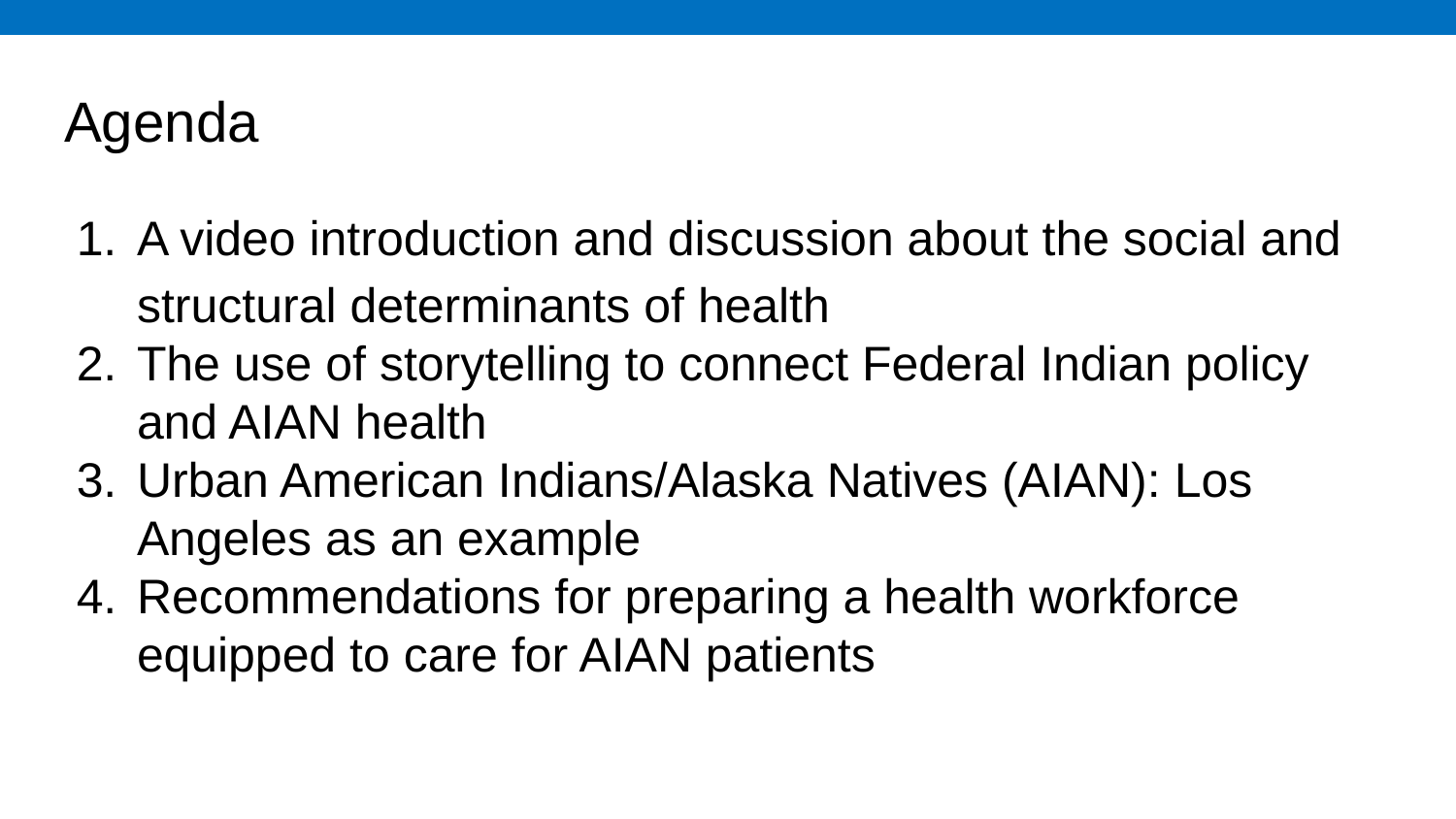

# Agenda
A video introduction and discussion about the social and structural determinants of health
The use of storytelling to connect Federal Indian policy and AIAN health
Urban American Indians/Alaska Natives (AIAN): Los Angeles as an example
Recommendations for preparing a health workforce equipped to care for AIAN patients

## Slide 7
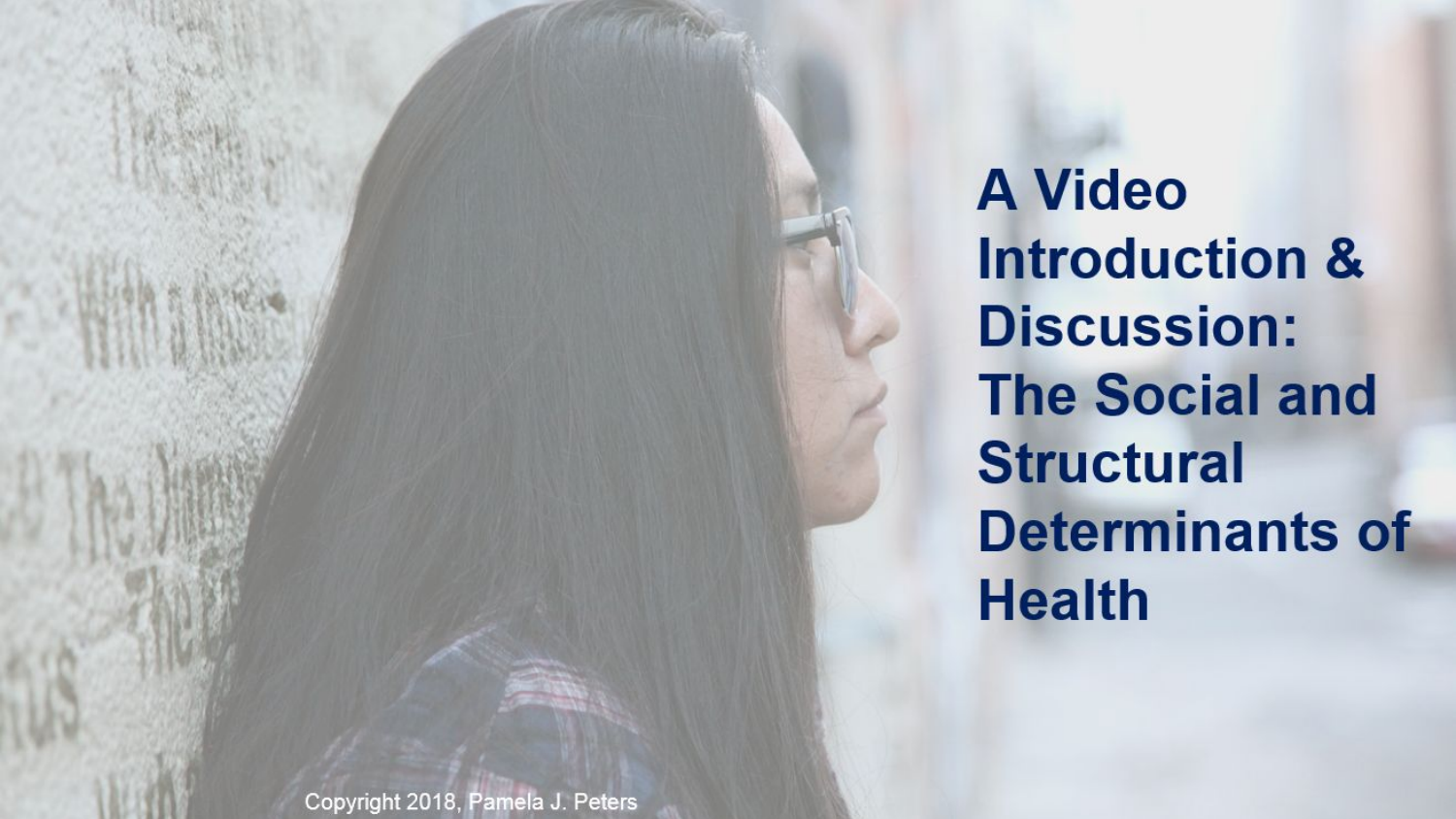

## Slide 8
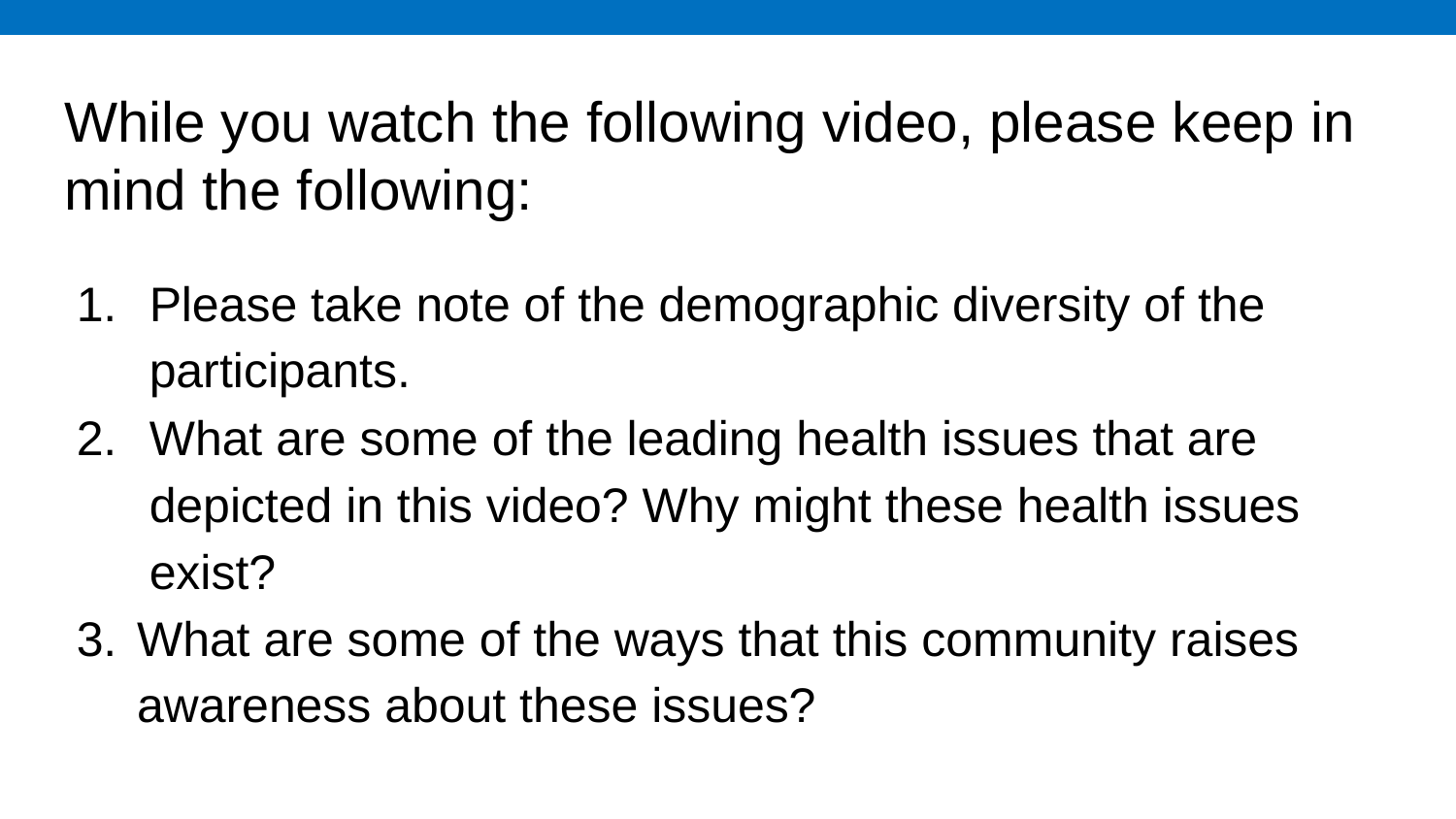

# While you watch the following video, please keep in mind the following:
Please take note of the demographic diversity of the participants.
What are some of the leading health issues that are depicted in this video? Why might these health issues exist?
What are some of the ways that this community raises awareness about these issues?

## Slide 9
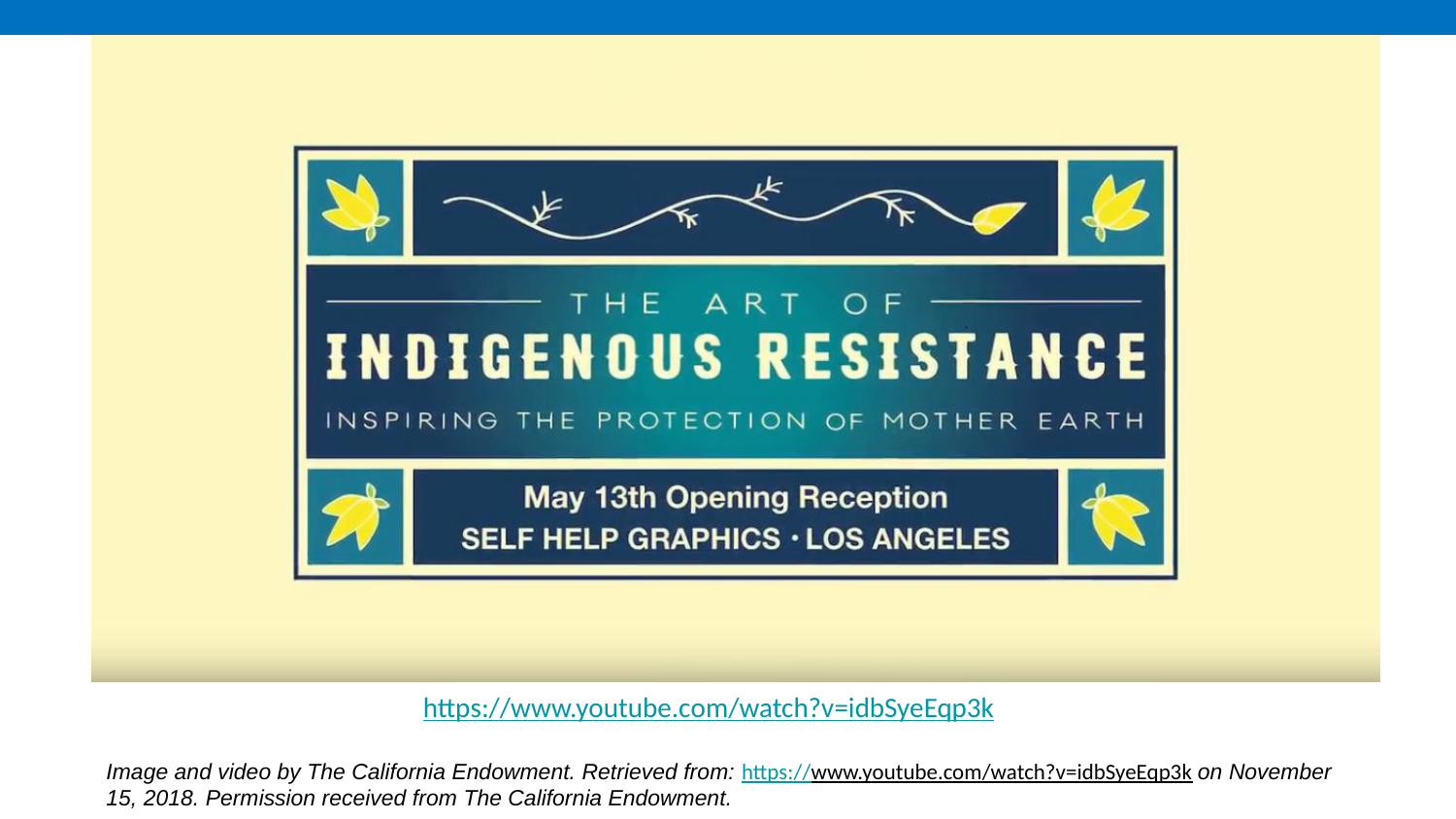

https://www.youtube.com/watch?v=idbSyeEqp3k
Image and video by The California Endowment. Retrieved from: https://www.youtube.com/watch?v=idbSyeEqp3k on November 15, 2018. Permission received from The California Endowment.

## Slide 10
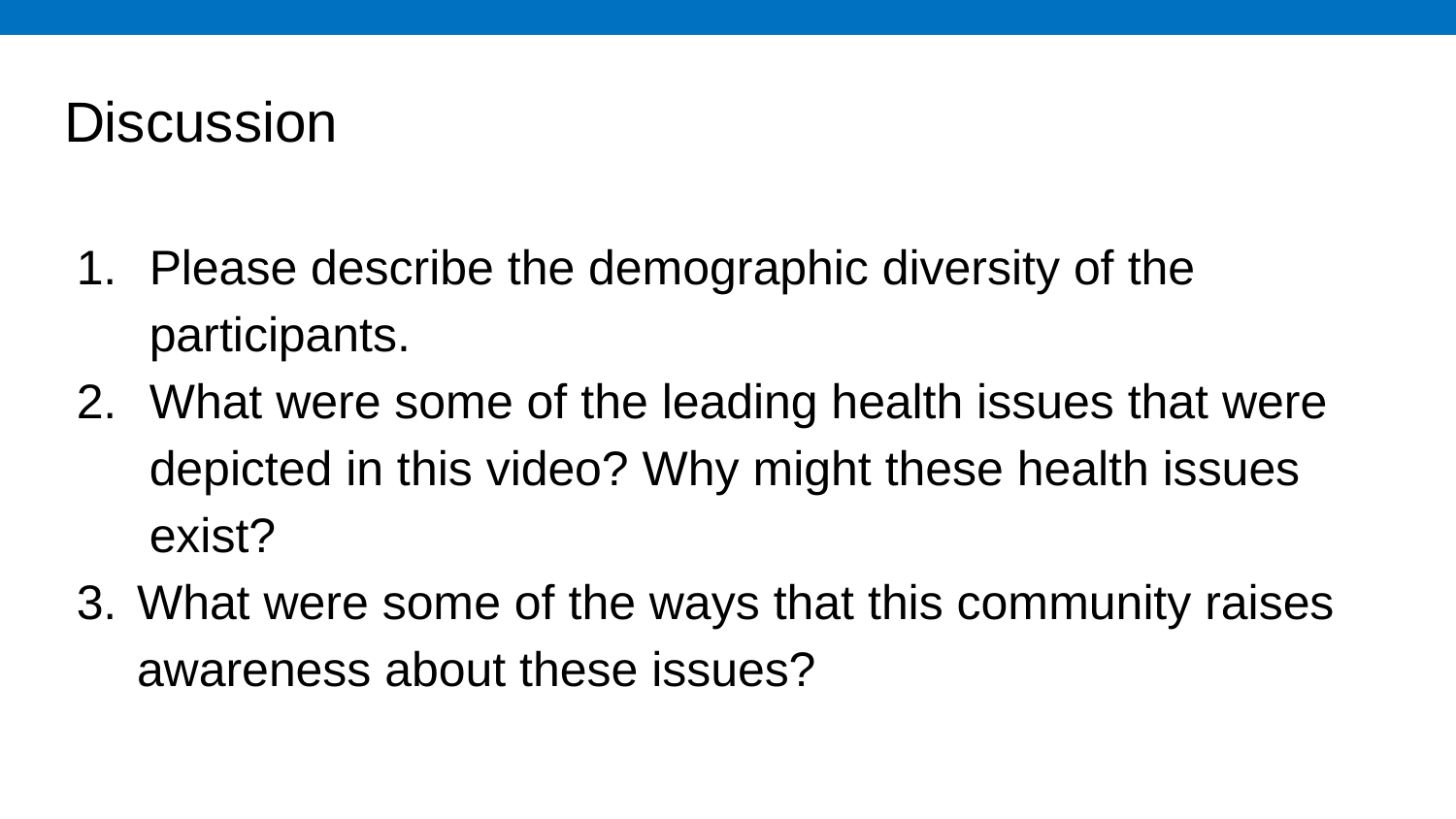

# Discussion
Please describe the demographic diversity of the participants.
What were some of the leading health issues that were depicted in this video? Why might these health issues exist?
What were some of the ways that this community raises awareness about these issues?

## Slide 11
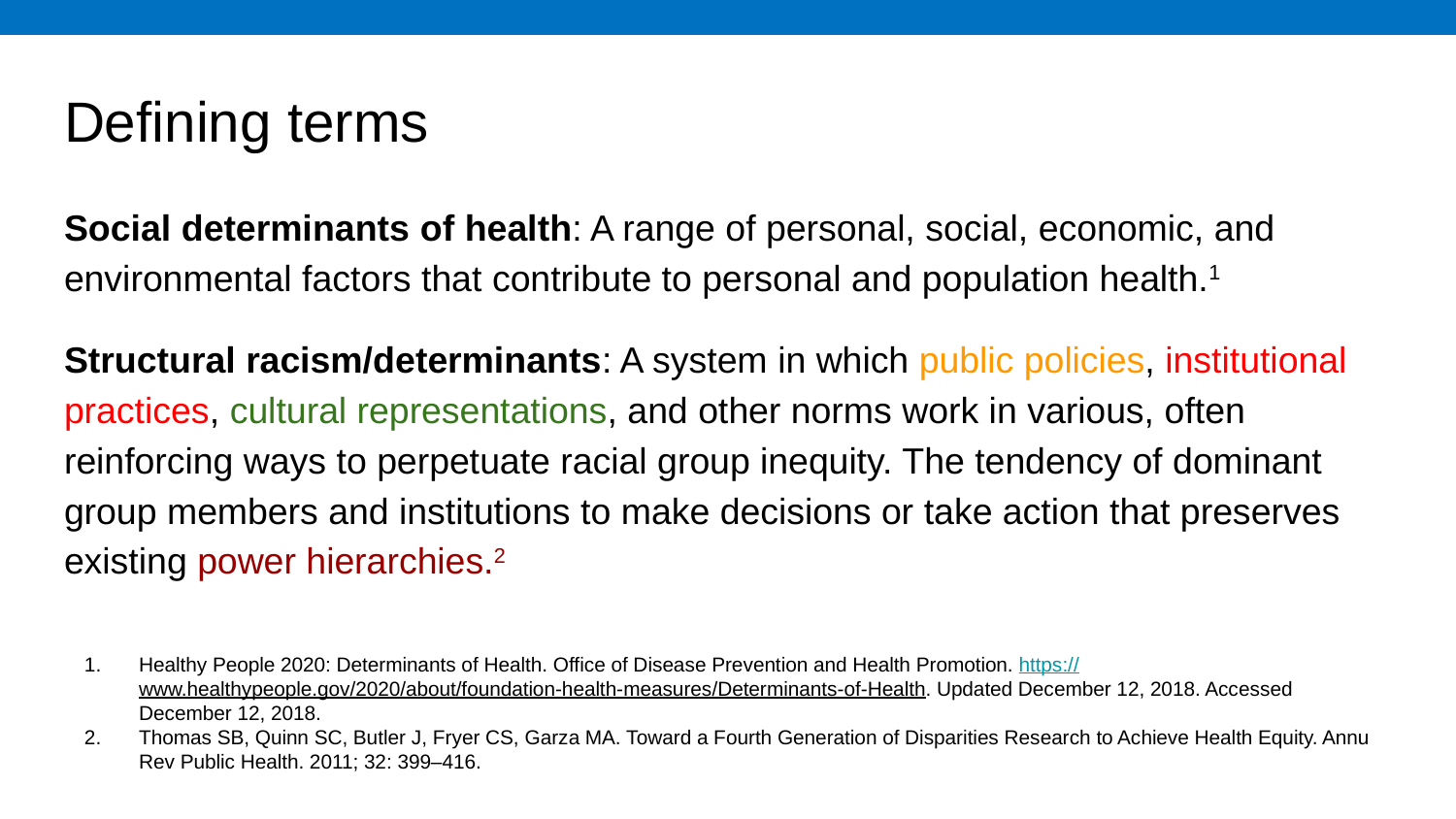

# Defining terms
Social determinants of health: A range of personal, social, economic, and environmental factors that contribute to personal and population health.1
Structural racism/determinants: A system in which public policies, institutional practices, cultural representations, and other norms work in various, often reinforcing ways to perpetuate racial group inequity. The tendency of dominant group members and institutions to make decisions or take action that preserves existing power hierarchies.2
Healthy People 2020: Determinants of Health. Office of Disease Prevention and Health Promotion. https://www.healthypeople.gov/2020/about/foundation-health-measures/Determinants-of-Health. Updated December 12, 2018. Accessed December 12, 2018.
Thomas SB, Quinn SC, Butler J, Fryer CS, Garza MA. Toward a Fourth Generation of Disparities Research to Achieve Health Equity. Annu Rev Public Health. 2011; 32: 399–416.

## Slide 12
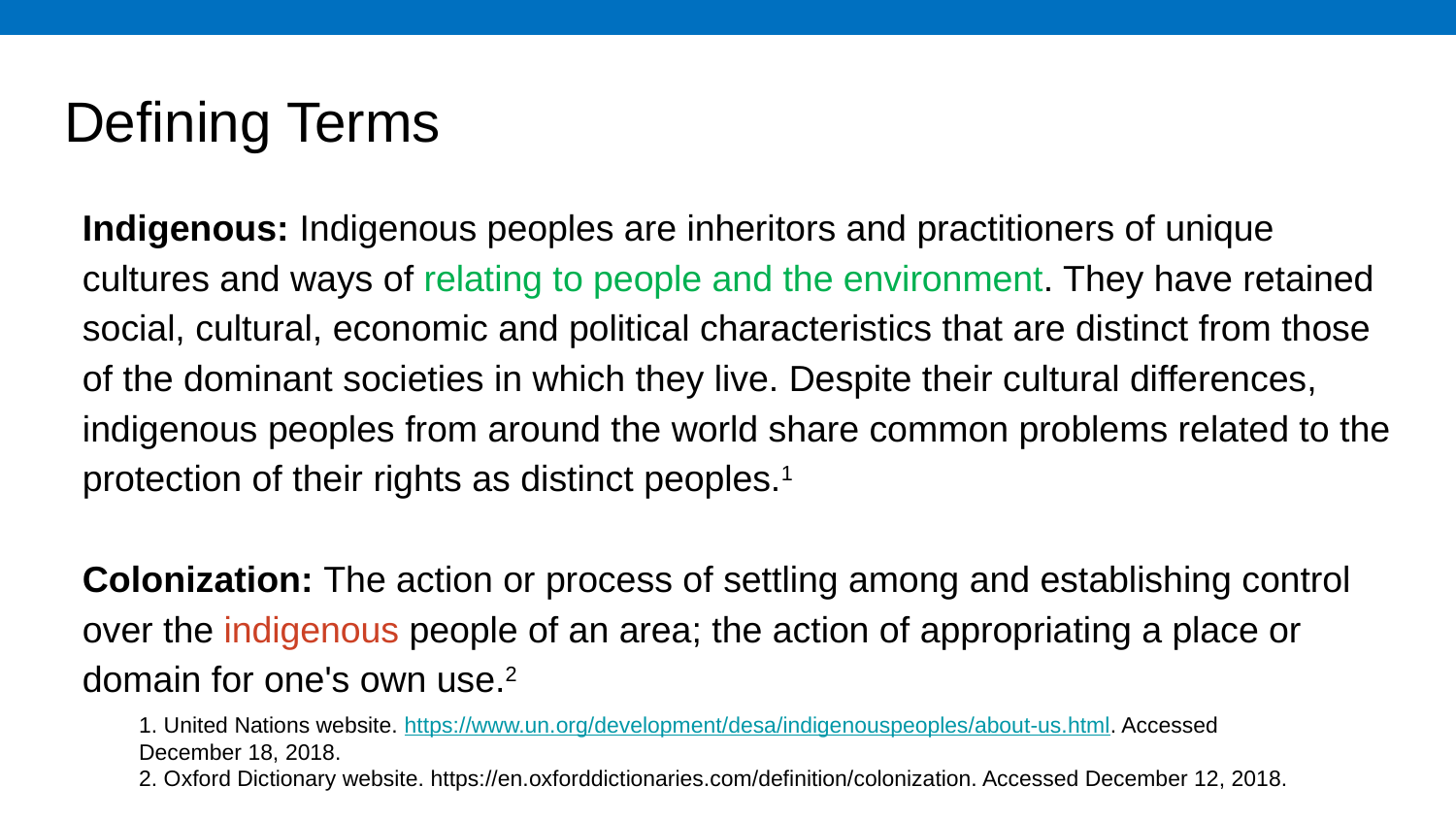

# Defining Terms
Indigenous: Indigenous peoples are inheritors and practitioners of unique cultures and ways of relating to people and the environment. They have retained social, cultural, economic and political characteristics that are distinct from those of the dominant societies in which they live. Despite their cultural differences, indigenous peoples from around the world share common problems related to the protection of their rights as distinct peoples.1
Colonization: The action or process of settling among and establishing control over the indigenous people of an area; the action of appropriating a place or domain for one's own use.2
1. United Nations website. https://www.un.org/development/desa/indigenouspeoples/about-us.html. Accessed December 18, 2018.
2. Oxford Dictionary website. https://en.oxforddictionaries.com/definition/colonization. Accessed December 12, 2018.

## Slide 13
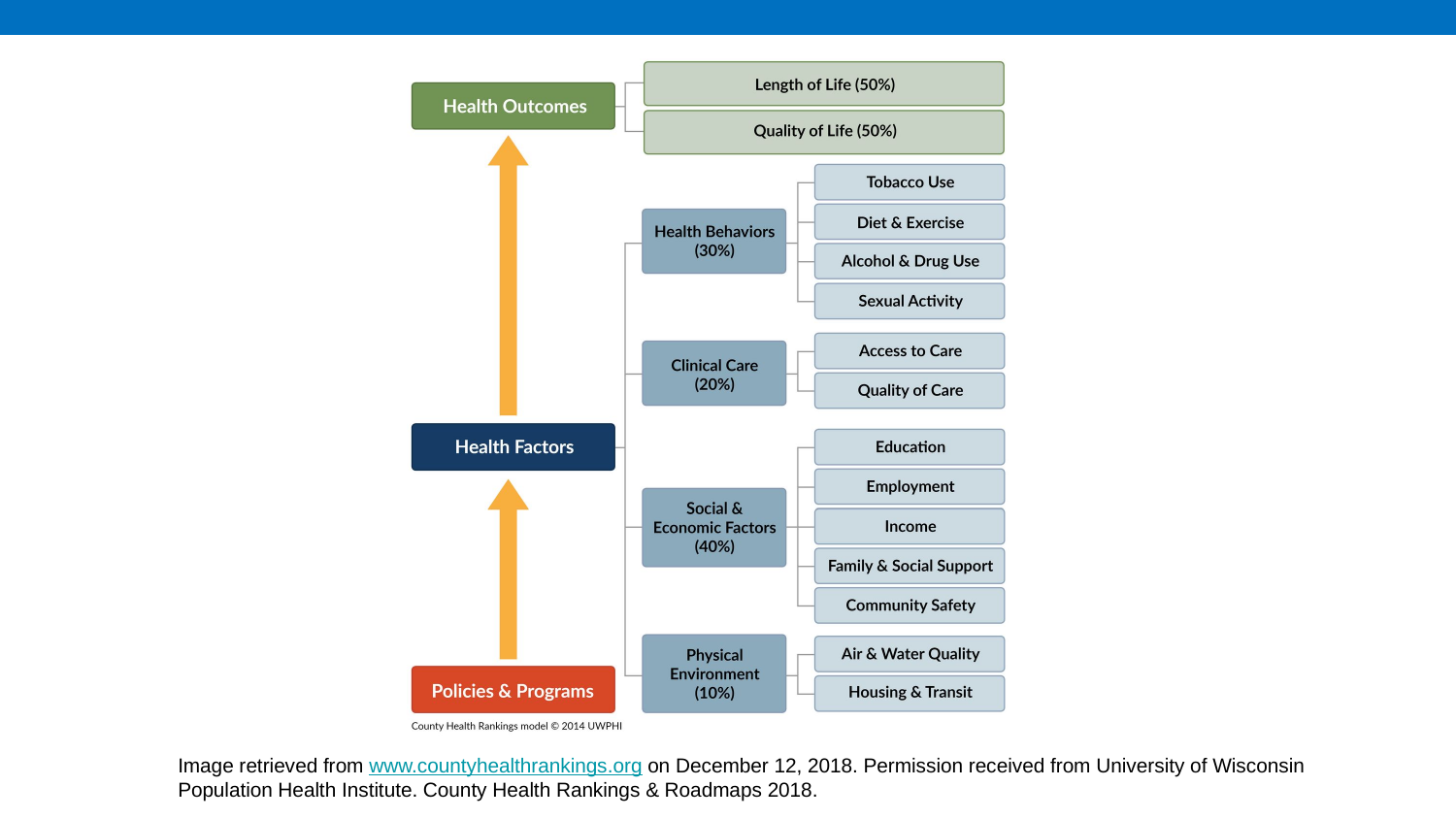

Image retrieved from www.countyhealthrankings.org on December 12, 2018. Permission received from University of Wisconsin Population Health Institute. County Health Rankings & Roadmaps 2018.

## Slide 14
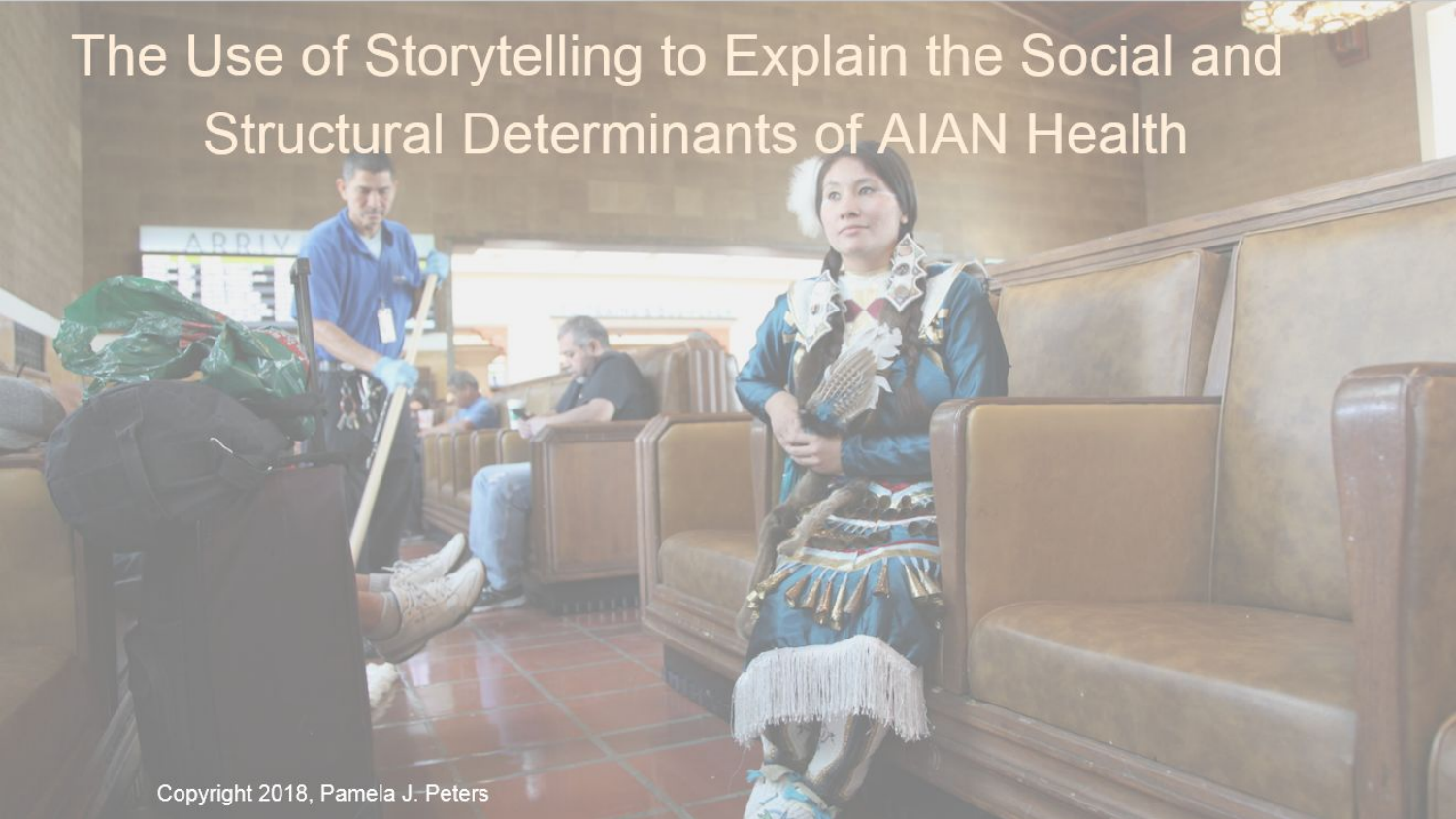

## Slide 15
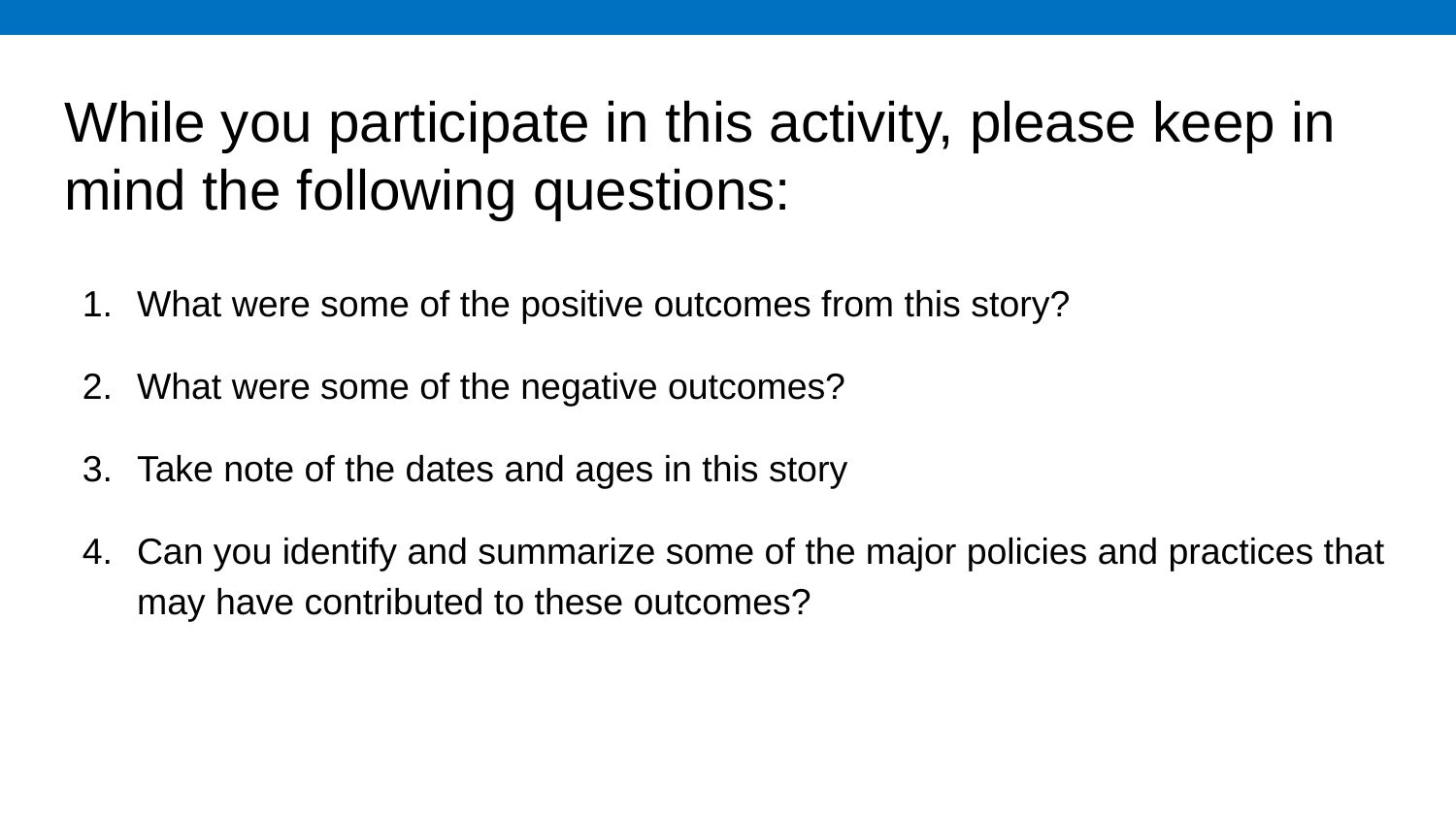

# While you participate in this activity, please keep in mind the following questions:
What were some of the positive outcomes from this story?
What were some of the negative outcomes?
Take note of the dates and ages in this story
Can you identify and summarize some of the major policies and practices that may have contributed to these outcomes?

## Slide 16
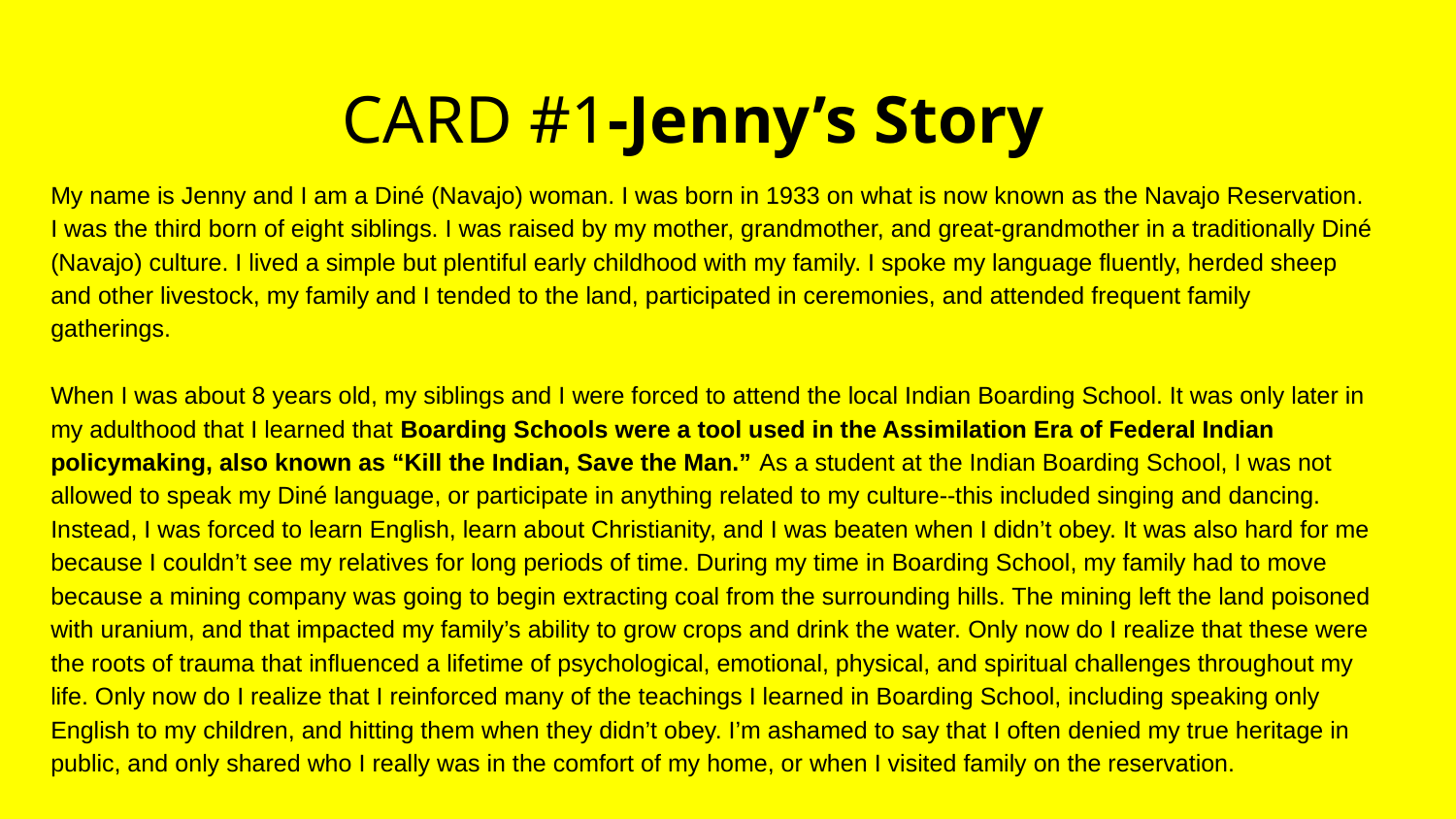

# CARD #1-Jenny’s Story
My name is Jenny and I am a Diné (Navajo) woman. I was born in 1933 on what is now known as the Navajo Reservation. I was the third born of eight siblings. I was raised by my mother, grandmother, and great-grandmother in a traditionally Diné (Navajo) culture. I lived a simple but plentiful early childhood with my family. I spoke my language fluently, herded sheep and other livestock, my family and I tended to the land, participated in ceremonies, and attended frequent family gatherings.
When I was about 8 years old, my siblings and I were forced to attend the local Indian Boarding School. It was only later in my adulthood that I learned that Boarding Schools were a tool used in the Assimilation Era of Federal Indian policymaking, also known as “Kill the Indian, Save the Man.” As a student at the Indian Boarding School, I was not allowed to speak my Diné language, or participate in anything related to my culture--this included singing and dancing. Instead, I was forced to learn English, learn about Christianity, and I was beaten when I didn’t obey. It was also hard for me because I couldn’t see my relatives for long periods of time. During my time in Boarding School, my family had to move because a mining company was going to begin extracting coal from the surrounding hills. The mining left the land poisoned with uranium, and that impacted my family’s ability to grow crops and drink the water. Only now do I realize that these were the roots of trauma that influenced a lifetime of psychological, emotional, physical, and spiritual challenges throughout my life. Only now do I realize that I reinforced many of the teachings I learned in Boarding School, including speaking only English to my children, and hitting them when they didn’t obey. I’m ashamed to say that I often denied my true heritage in public, and only shared who I really was in the comfort of my home, or when I visited family on the reservation.

## Slide 17
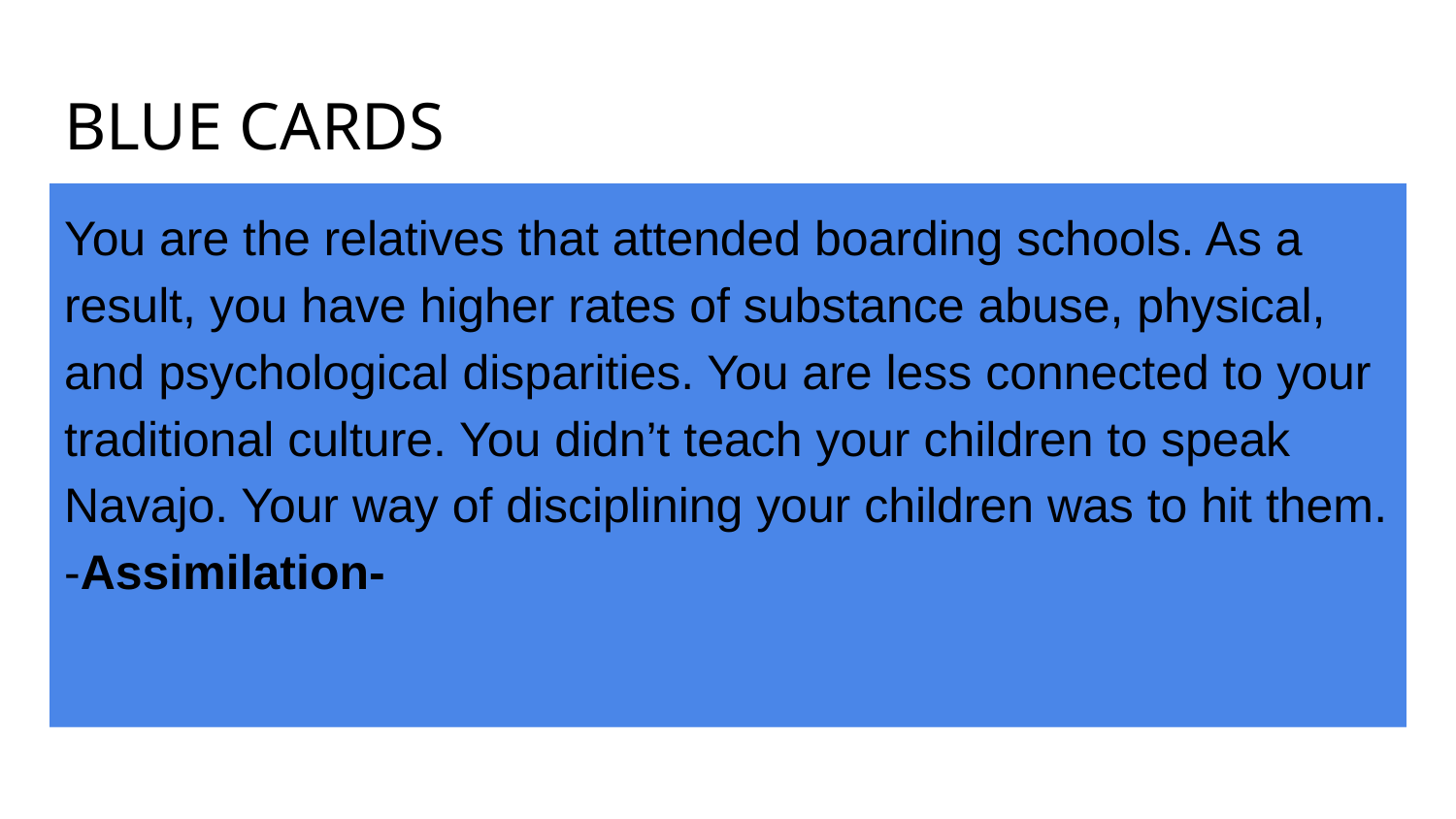

# BLUE CARDS
You are the relatives that attended boarding schools. As a result, you have higher rates of substance abuse, physical, and psychological disparities. You are less connected to your traditional culture. You didn’t teach your children to speak Navajo. Your way of disciplining your children was to hit them. -Assimilation-

## Slide 18
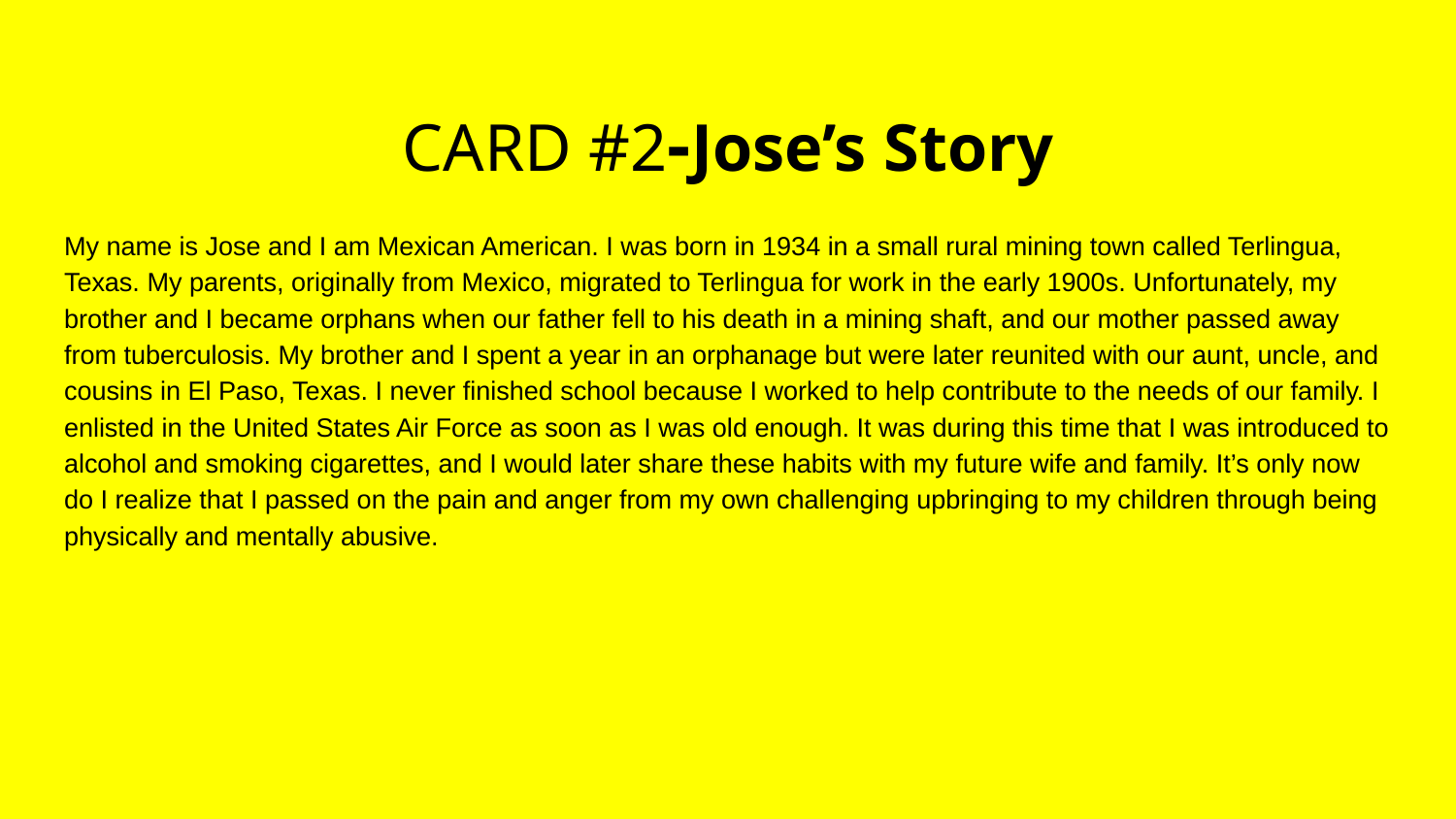

# CARD #2-Jose’s Story
My name is Jose and I am Mexican American. I was born in 1934 in a small rural mining town called Terlingua, Texas. My parents, originally from Mexico, migrated to Terlingua for work in the early 1900s. Unfortunately, my brother and I became orphans when our father fell to his death in a mining shaft, and our mother passed away from tuberculosis. My brother and I spent a year in an orphanage but were later reunited with our aunt, uncle, and cousins in El Paso, Texas. I never finished school because I worked to help contribute to the needs of our family. I enlisted in the United States Air Force as soon as I was old enough. It was during this time that I was introduced to alcohol and smoking cigarettes, and I would later share these habits with my future wife and family. It’s only now do I realize that I passed on the pain and anger from my own challenging upbringing to my children through being physically and mentally abusive.

## Slide 19
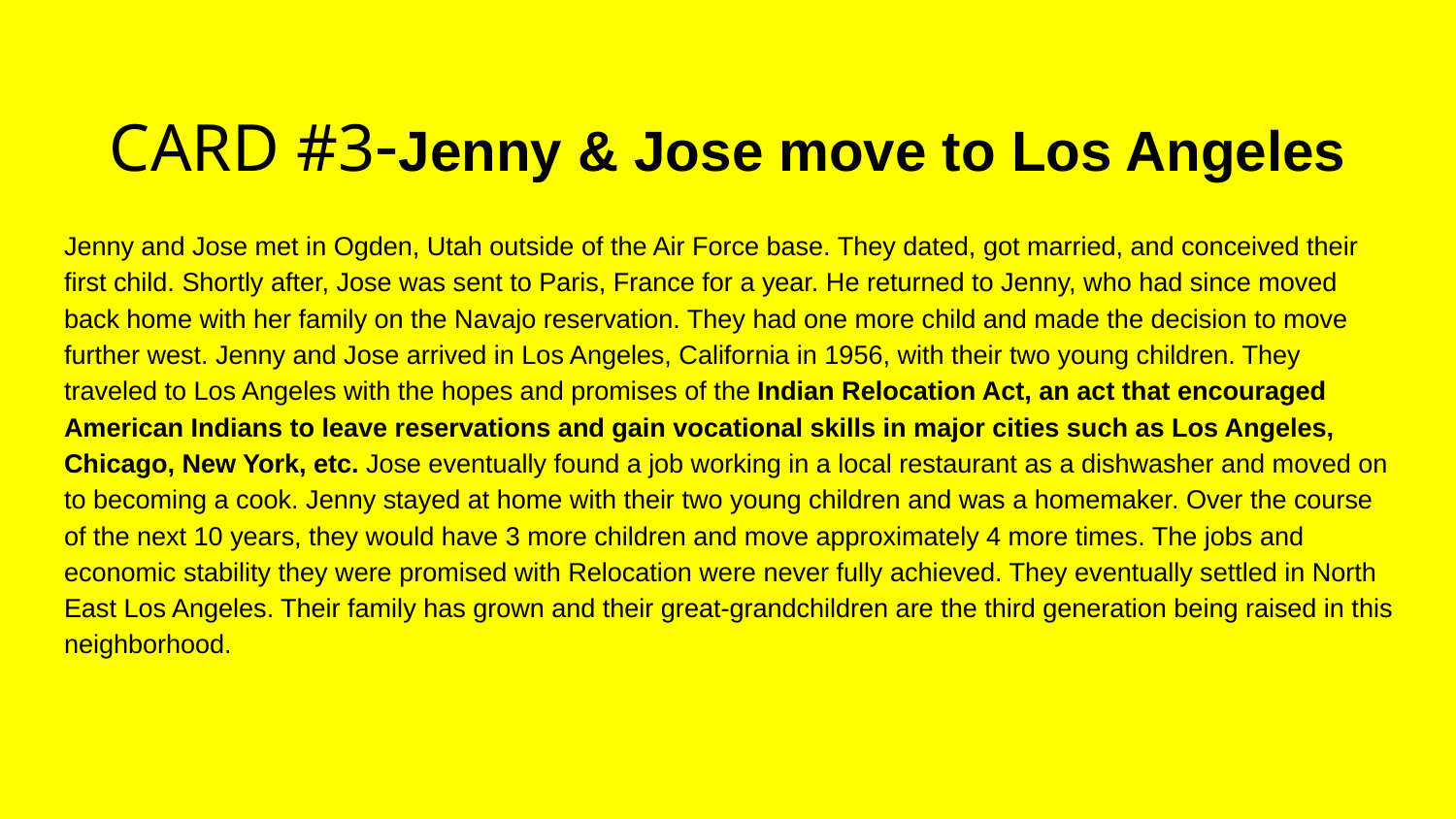

# CARD #3-Jenny & Jose move to Los Angeles
Jenny and Jose met in Ogden, Utah outside of the Air Force base. They dated, got married, and conceived their first child. Shortly after, Jose was sent to Paris, France for a year. He returned to Jenny, who had since moved back home with her family on the Navajo reservation. They had one more child and made the decision to move further west. Jenny and Jose arrived in Los Angeles, California in 1956, with their two young children. They traveled to Los Angeles with the hopes and promises of the Indian Relocation Act, an act that encouraged American Indians to leave reservations and gain vocational skills in major cities such as Los Angeles, Chicago, New York, etc. Jose eventually found a job working in a local restaurant as a dishwasher and moved on to becoming a cook. Jenny stayed at home with their two young children and was a homemaker. Over the course of the next 10 years, they would have 3 more children and move approximately 4 more times. The jobs and economic stability they were promised with Relocation were never fully achieved. They eventually settled in North East Los Angeles. Their family has grown and their great-grandchildren are the third generation being raised in this neighborhood.

## Slide 20
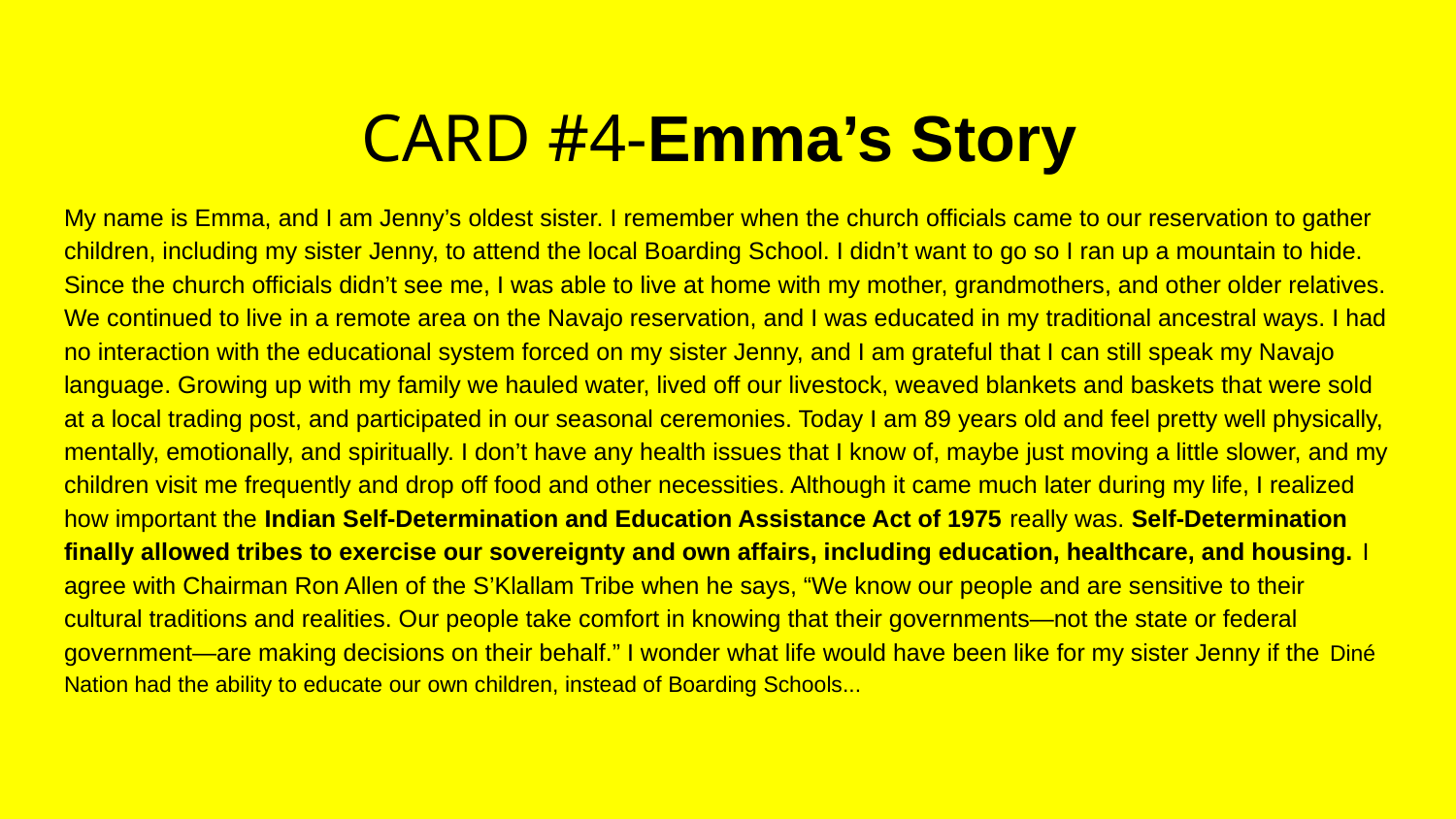

# CARD #4-Emma’s Story
My name is Emma, and I am Jenny’s oldest sister. I remember when the church officials came to our reservation to gather children, including my sister Jenny, to attend the local Boarding School. I didn’t want to go so I ran up a mountain to hide. Since the church officials didn’t see me, I was able to live at home with my mother, grandmothers, and other older relatives. We continued to live in a remote area on the Navajo reservation, and I was educated in my traditional ancestral ways. I had no interaction with the educational system forced on my sister Jenny, and I am grateful that I can still speak my Navajo language. Growing up with my family we hauled water, lived off our livestock, weaved blankets and baskets that were sold at a local trading post, and participated in our seasonal ceremonies. Today I am 89 years old and feel pretty well physically, mentally, emotionally, and spiritually. I don’t have any health issues that I know of, maybe just moving a little slower, and my children visit me frequently and drop off food and other necessities. Although it came much later during my life, I realized how important the Indian Self-Determination and Education Assistance Act of 1975 really was. Self-Determination finally allowed tribes to exercise our sovereignty and own affairs, including education, healthcare, and housing. I agree with Chairman Ron Allen of the S’Klallam Tribe when he says, “We know our people and are sensitive to their cultural traditions and realities. Our people take comfort in knowing that their governments—not the state or federal government—are making decisions on their behalf.” I wonder what life would have been like for my sister Jenny if the Diné Nation had the ability to educate our own children, instead of Boarding Schools...

## Slide 21
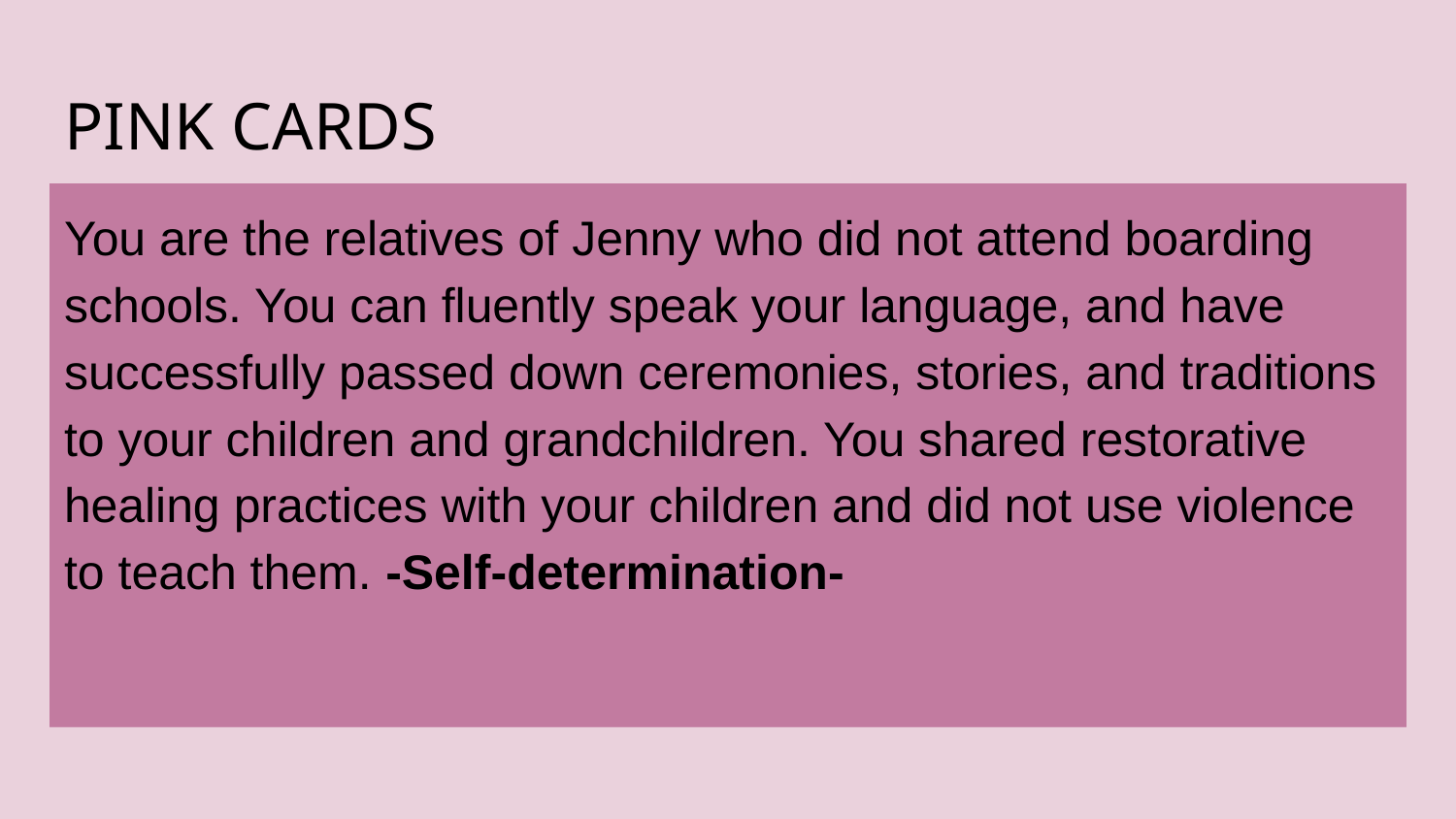

# PINK CARDS
You are the relatives of Jenny who did not attend boarding schools. You can fluently speak your language, and have successfully passed down ceremonies, stories, and traditions to your children and grandchildren. You shared restorative healing practices with your children and did not use violence to teach them. -Self-determination-

## Slide 22
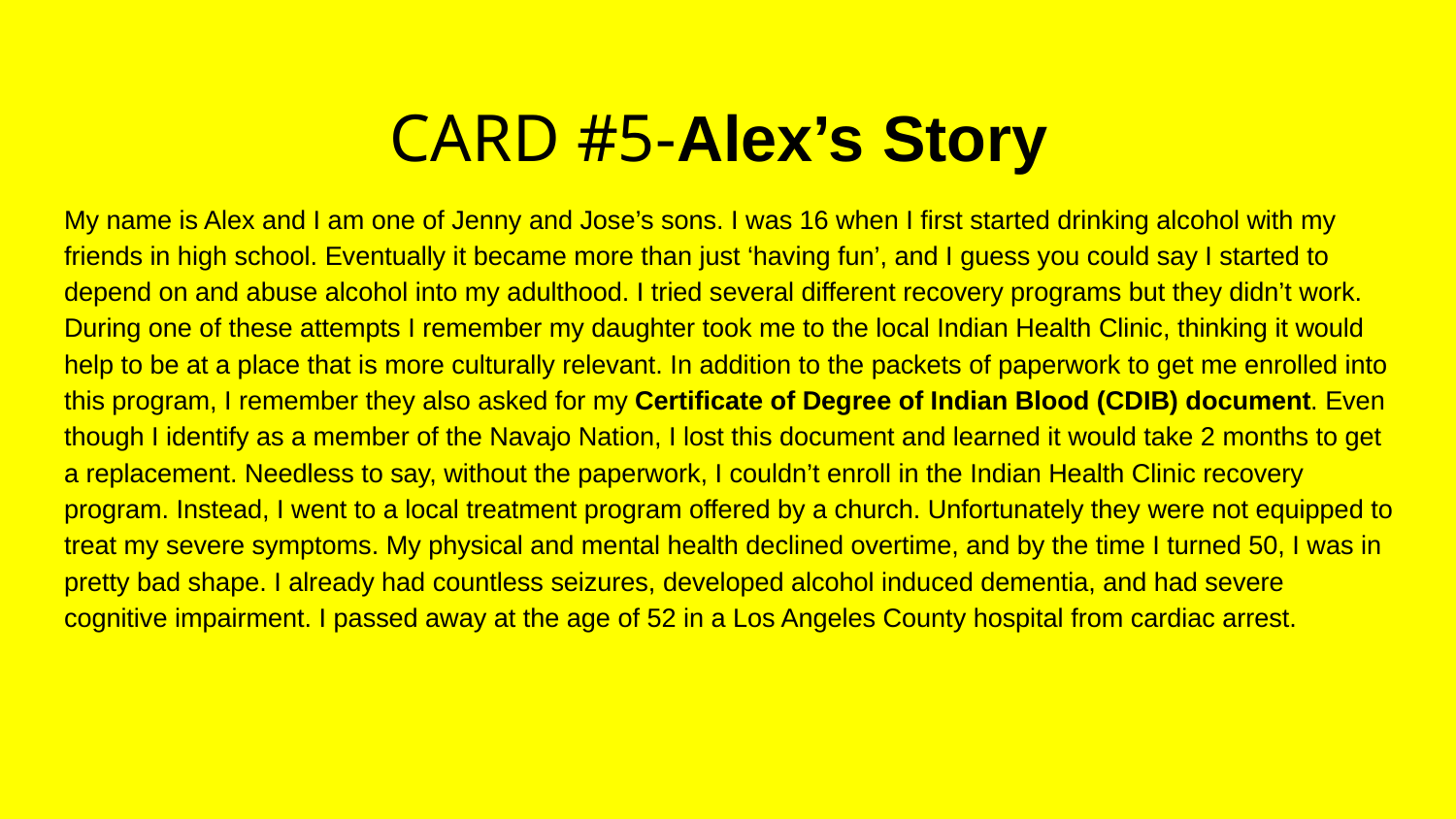

# CARD #5-Alex’s Story
My name is Alex and I am one of Jenny and Jose’s sons. I was 16 when I first started drinking alcohol with my friends in high school. Eventually it became more than just ‘having fun’, and I guess you could say I started to depend on and abuse alcohol into my adulthood. I tried several different recovery programs but they didn’t work. During one of these attempts I remember my daughter took me to the local Indian Health Clinic, thinking it would help to be at a place that is more culturally relevant. In addition to the packets of paperwork to get me enrolled into this program, I remember they also asked for my Certificate of Degree of Indian Blood (CDIB) document. Even though I identify as a member of the Navajo Nation, I lost this document and learned it would take 2 months to get a replacement. Needless to say, without the paperwork, I couldn’t enroll in the Indian Health Clinic recovery program. Instead, I went to a local treatment program offered by a church. Unfortunately they were not equipped to treat my severe symptoms. My physical and mental health declined overtime, and by the time I turned 50, I was in pretty bad shape. I already had countless seizures, developed alcohol induced dementia, and had severe cognitive impairment. I passed away at the age of 52 in a Los Angeles County hospital from cardiac arrest.

## Slide 23
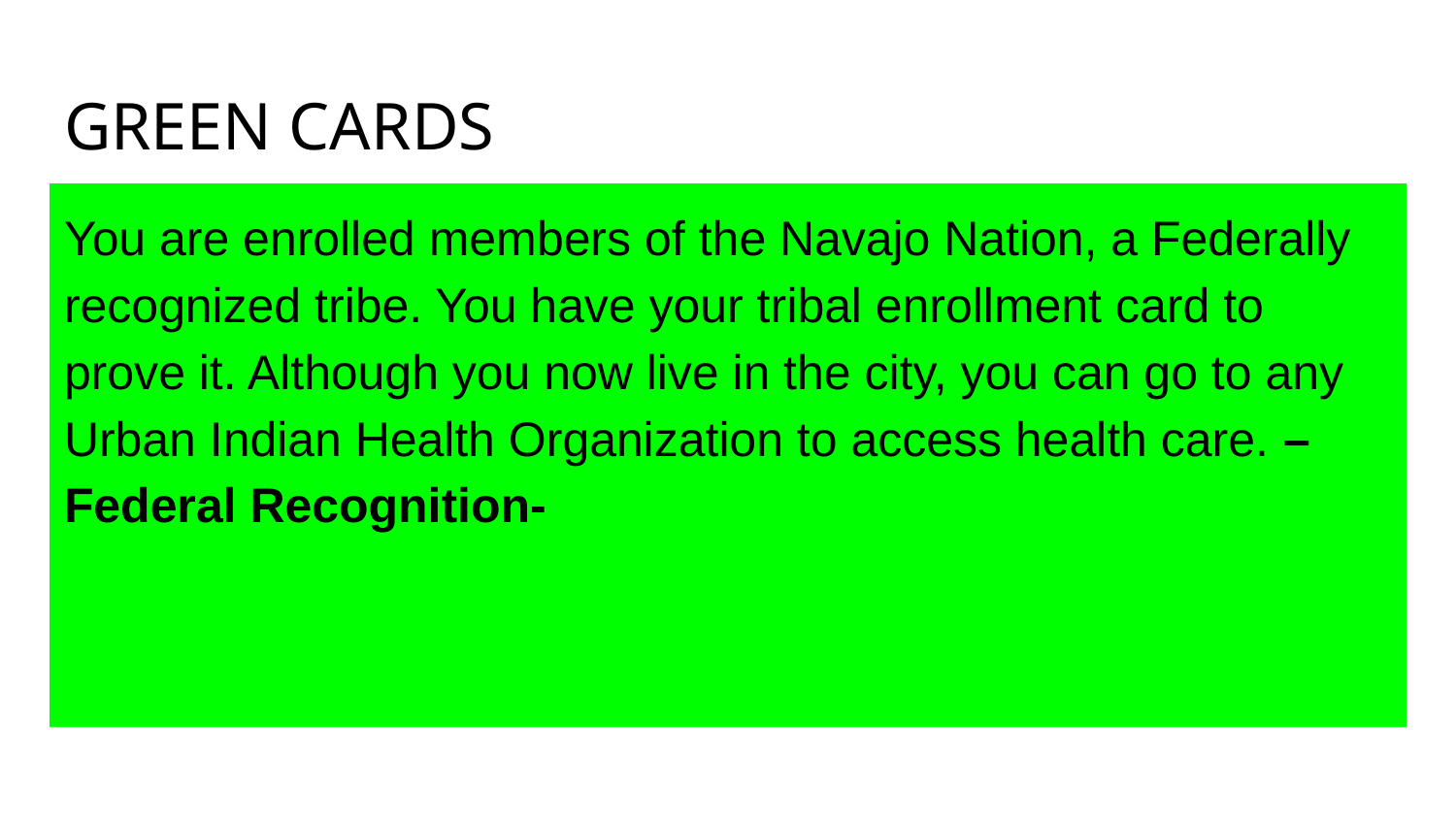

# GREEN CARDS
You are enrolled members of the Navajo Nation, a Federally recognized tribe. You have your tribal enrollment card to prove it. Although you now live in the city, you can go to any Urban Indian Health Organization to access health care. –Federal Recognition-

## Slide 24
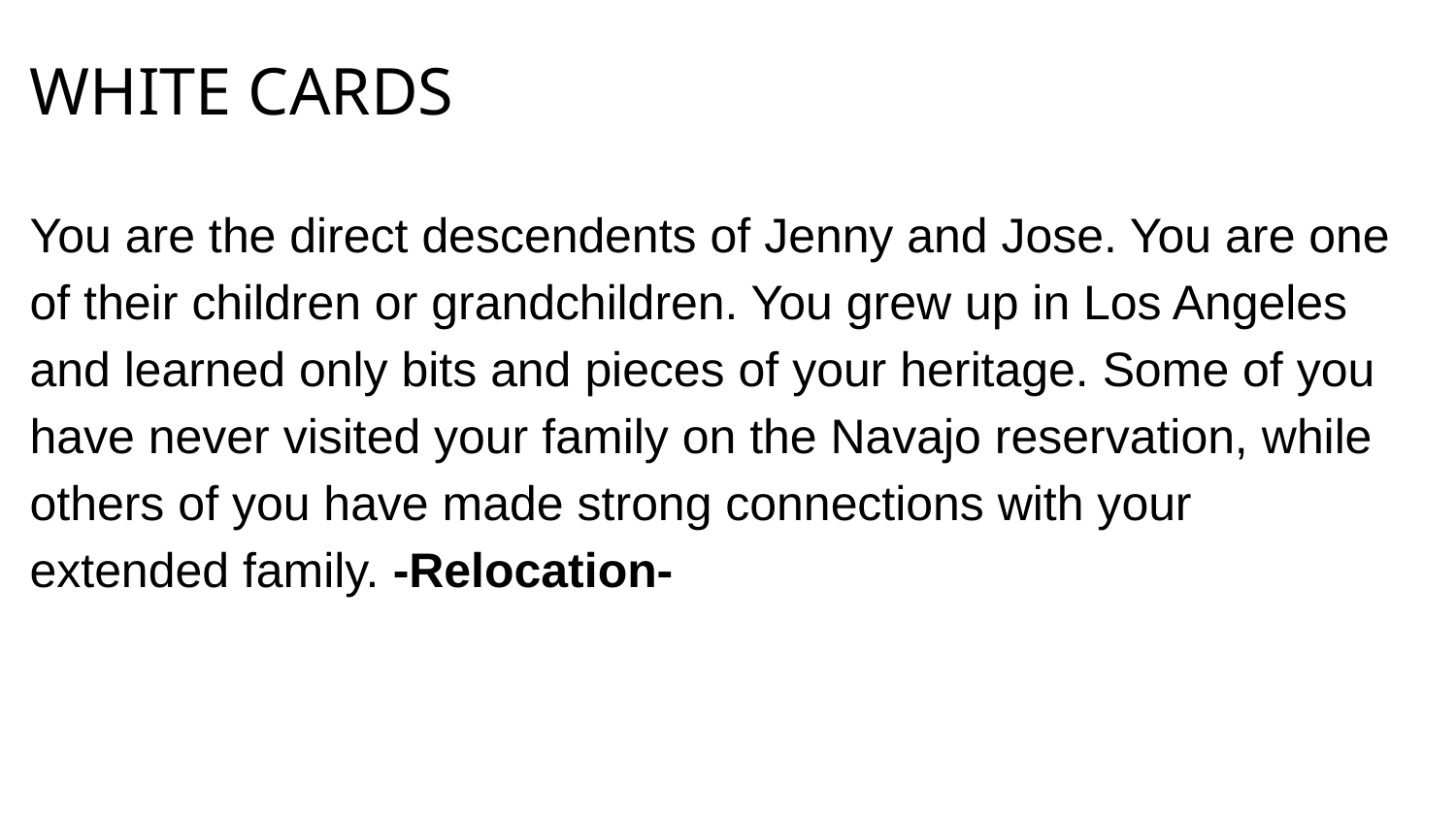

WHITE CARDS
You are the direct descendents of Jenny and Jose. You are one of their children or grandchildren. You grew up in Los Angeles and learned only bits and pieces of your heritage. Some of you have never visited your family on the Navajo reservation, while others of you have made strong connections with your extended family. -Relocation-
#

## Slide 25
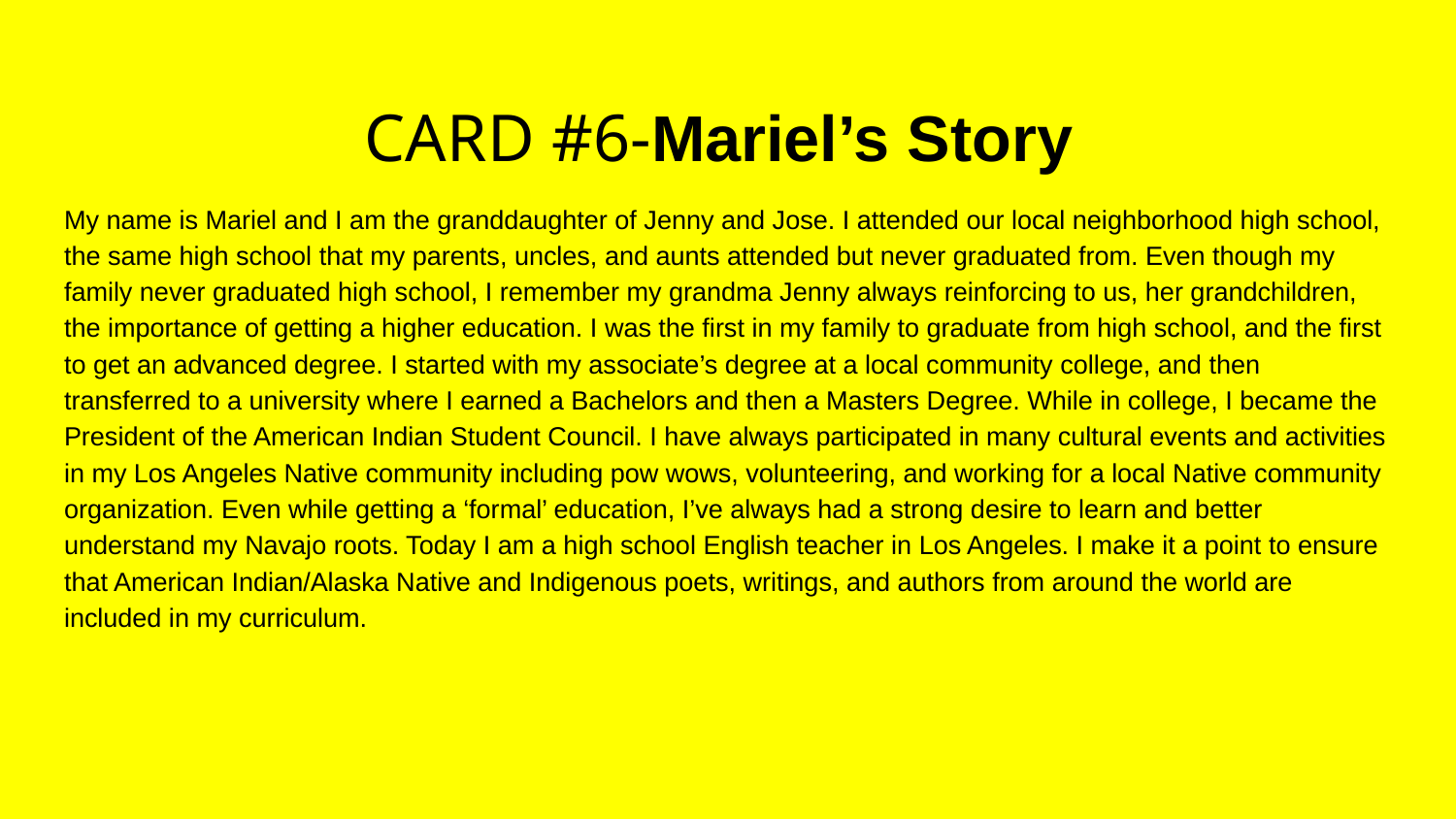

# CARD #6-Mariel’s Story
My name is Mariel and I am the granddaughter of Jenny and Jose. I attended our local neighborhood high school, the same high school that my parents, uncles, and aunts attended but never graduated from. Even though my family never graduated high school, I remember my grandma Jenny always reinforcing to us, her grandchildren, the importance of getting a higher education. I was the first in my family to graduate from high school, and the first to get an advanced degree. I started with my associate’s degree at a local community college, and then transferred to a university where I earned a Bachelors and then a Masters Degree. While in college, I became the President of the American Indian Student Council. I have always participated in many cultural events and activities in my Los Angeles Native community including pow wows, volunteering, and working for a local Native community organization. Even while getting a ‘formal’ education, I’ve always had a strong desire to learn and better understand my Navajo roots. Today I am a high school English teacher in Los Angeles. I make it a point to ensure that American Indian/Alaska Native and Indigenous poets, writings, and authors from around the world are included in my curriculum.

## Slide 26
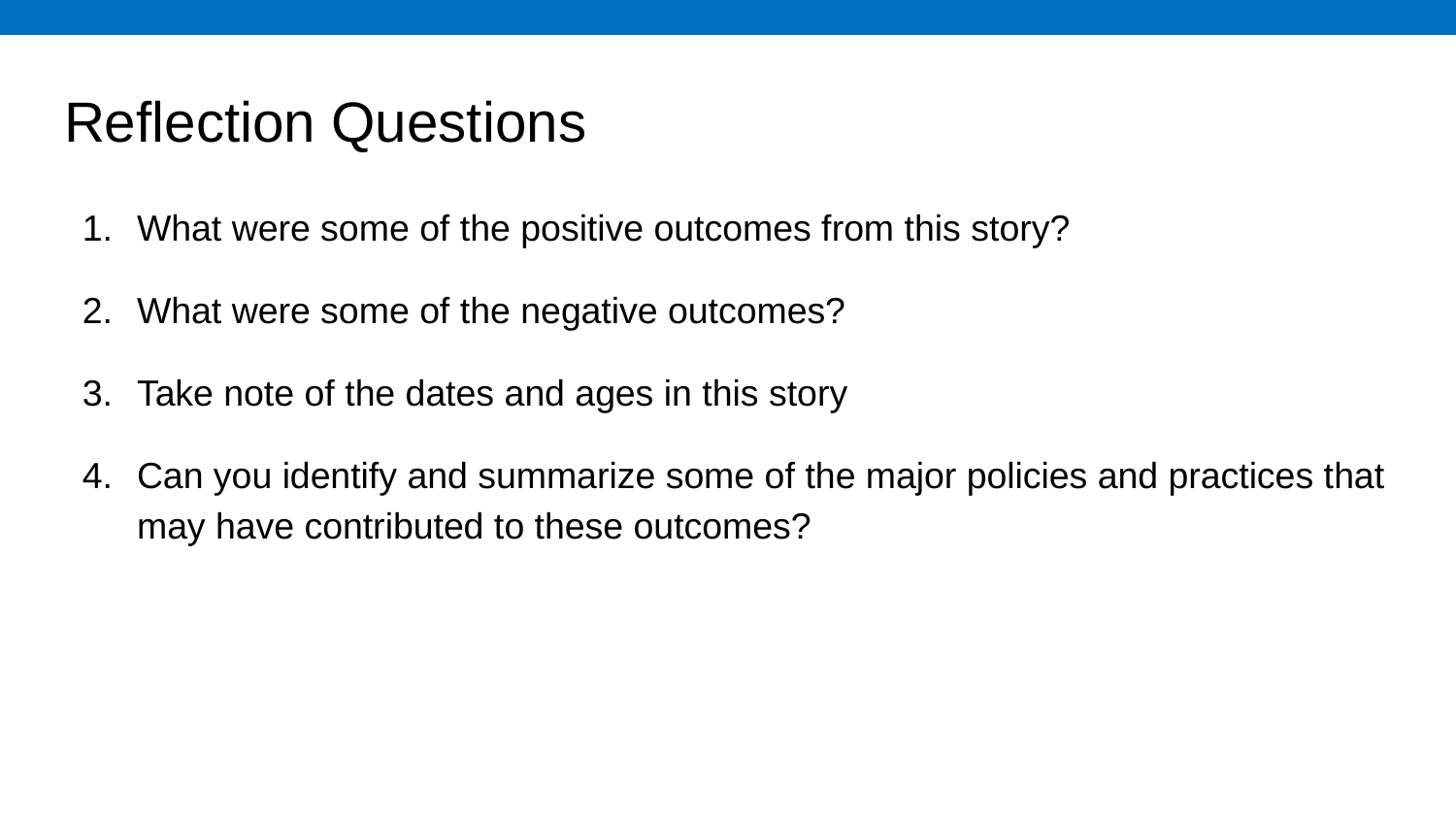

# Reflection Questions
What were some of the positive outcomes from this story?
What were some of the negative outcomes?
Take note of the dates and ages in this story
Can you identify and summarize some of the major policies and practices that may have contributed to these outcomes?

## Slide 27
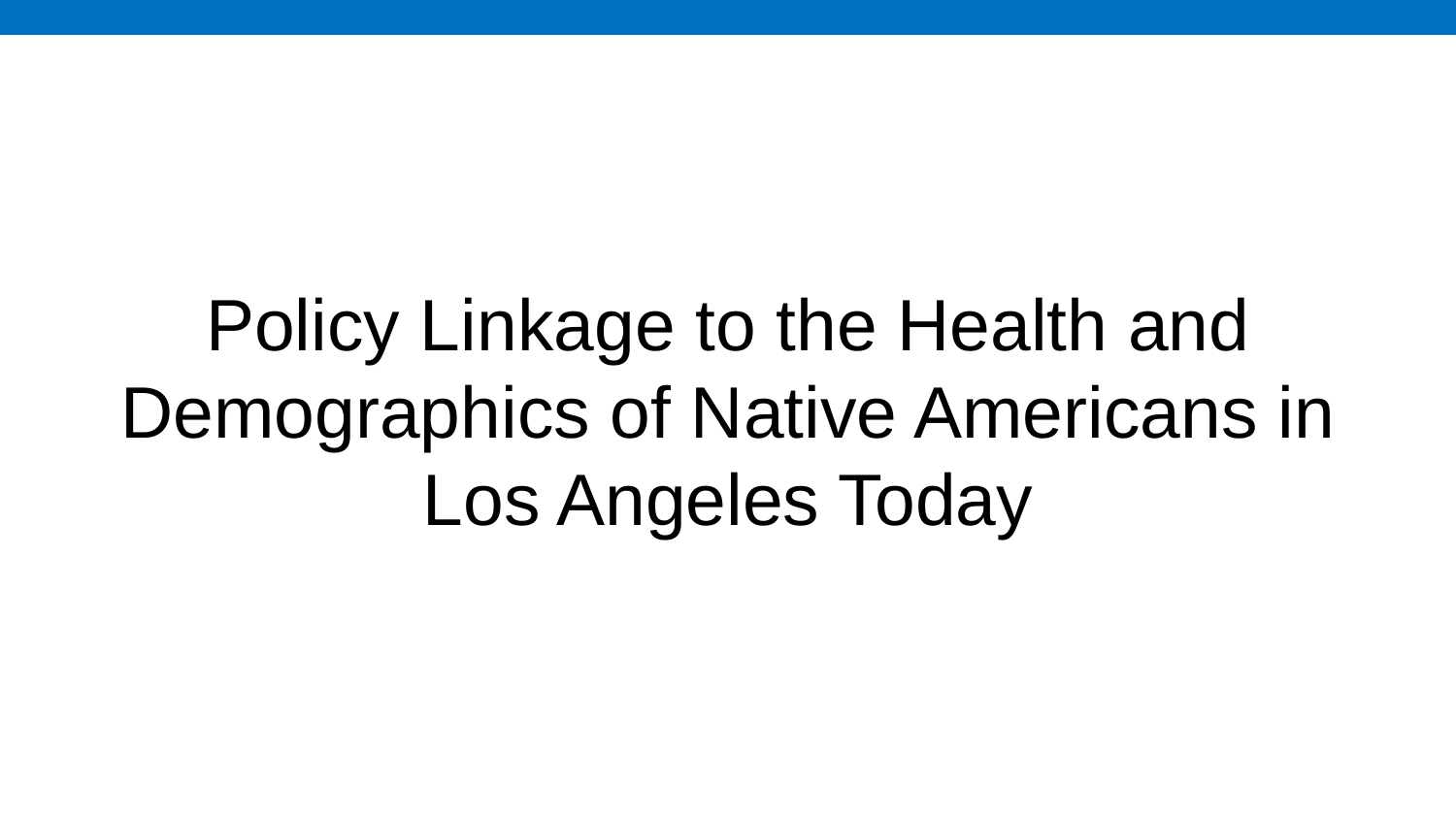

# Policy Linkage to the Health and Demographics of Native Americans in Los Angeles Today

## Slide 28
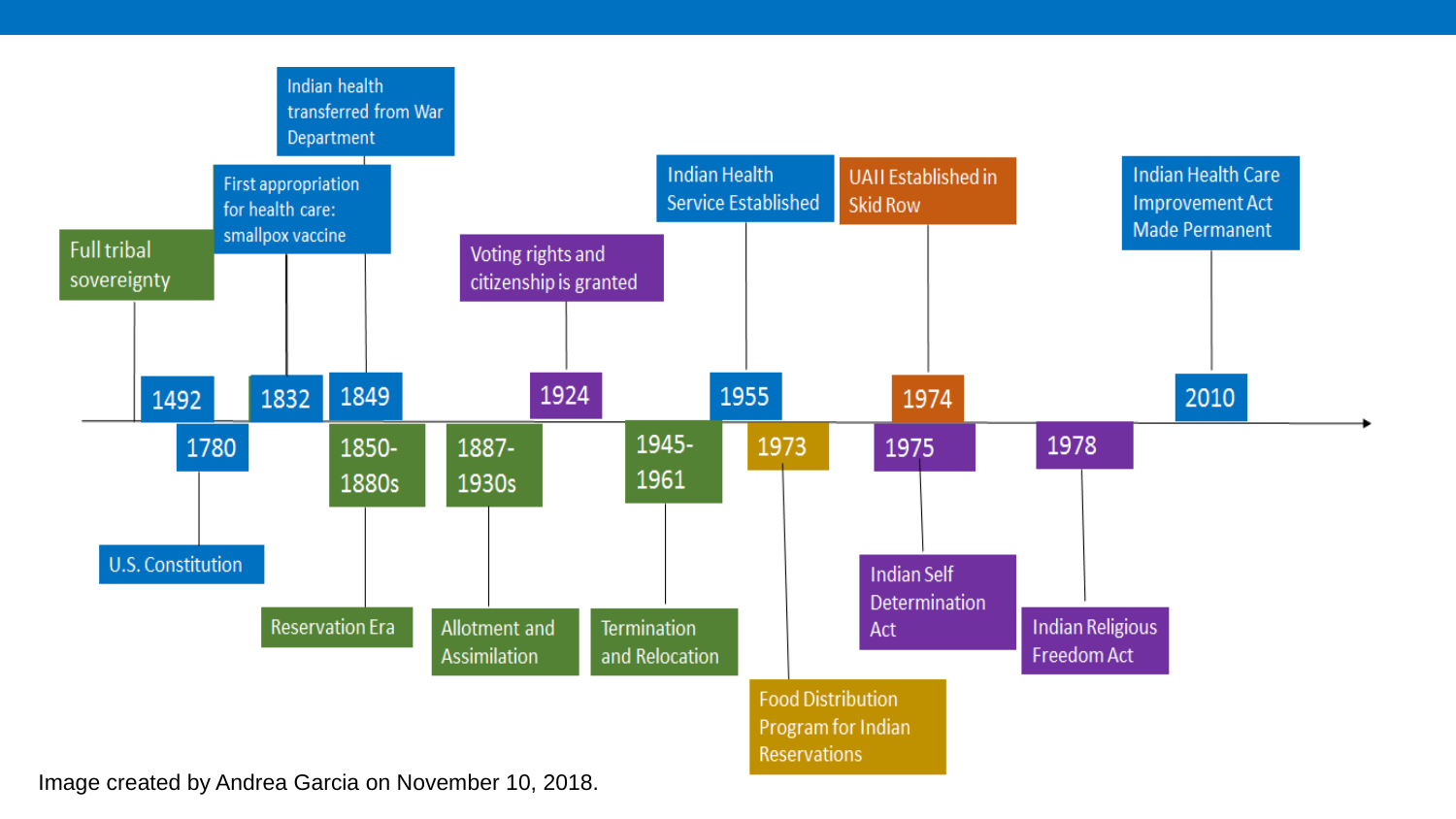

Image created by Andrea Garcia on November 10, 2018.

## Slide 29
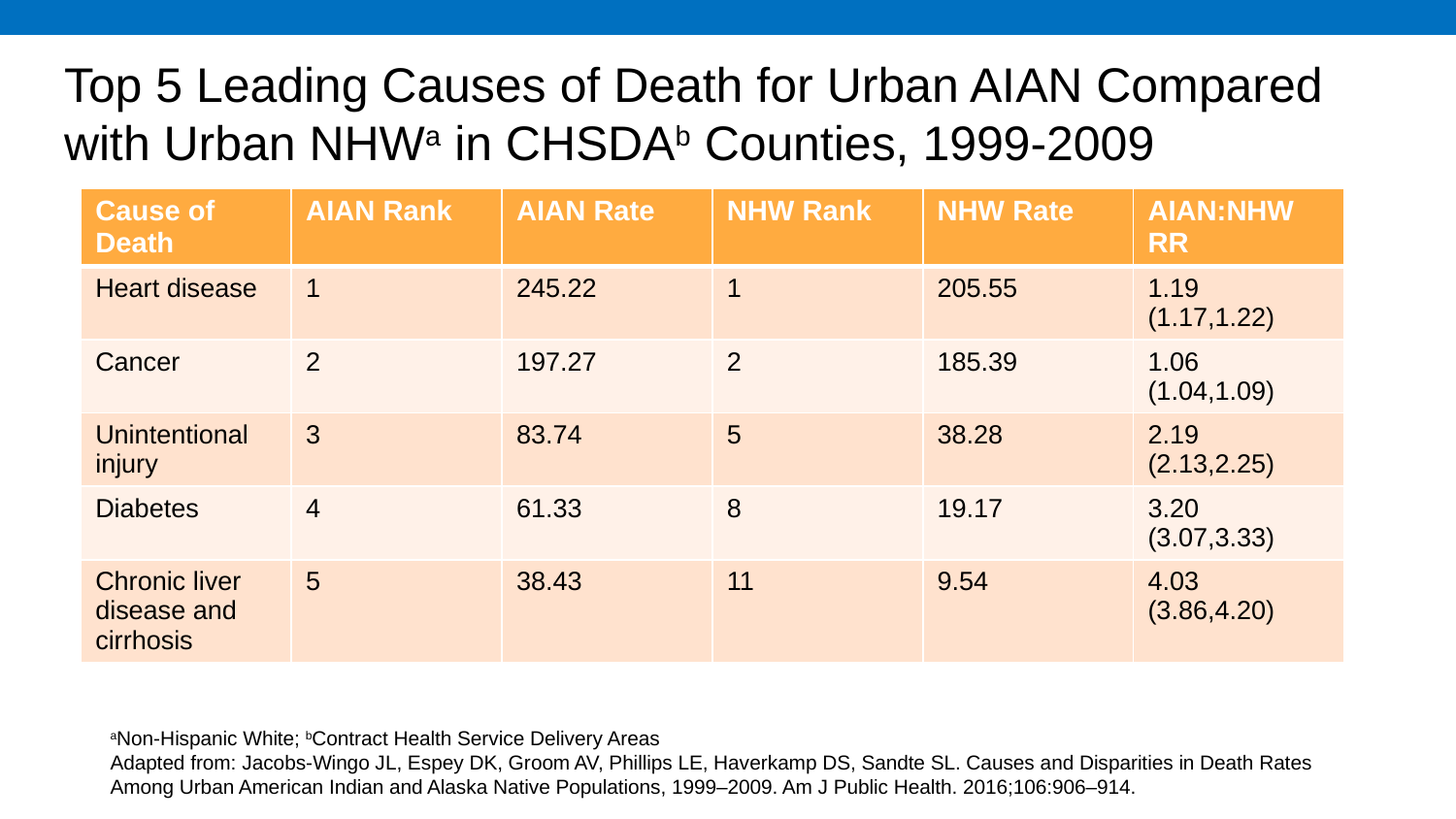

# Top 5 Leading Causes of Death for Urban AIAN Compared with Urban NHWa in CHSDAb Counties, 1999-2009
| Cause of Death | AIAN Rank | AIAN Rate | NHW Rank | NHW Rate | AIAN:NHW RR |
| --- | --- | --- | --- | --- | --- |
| Heart disease | 1 | 245.22 | 1 | 205.55 | 1.19 (1.17,1.22) |
| Cancer | 2 | 197.27 | 2 | 185.39 | 1.06 (1.04,1.09) |
| Unintentional injury | 3 | 83.74 | 5 | 38.28 | 2.19 (2.13,2.25) |
| Diabetes | 4 | 61.33 | 8 | 19.17 | 3.20 (3.07,3.33) |
| Chronic liver disease and cirrhosis | 5 | 38.43 | 11 | 9.54 | 4.03 (3.86,4.20) |
aNon-Hispanic White; bContract Health Service Delivery Areas
Adapted from: Jacobs-Wingo JL, Espey DK, Groom AV, Phillips LE, Haverkamp DS, Sandte SL. Causes and Disparities in Death Rates Among Urban American Indian and Alaska Native Populations, 1999–2009. Am J Public Health. 2016;106:906–914.

## Slide 30
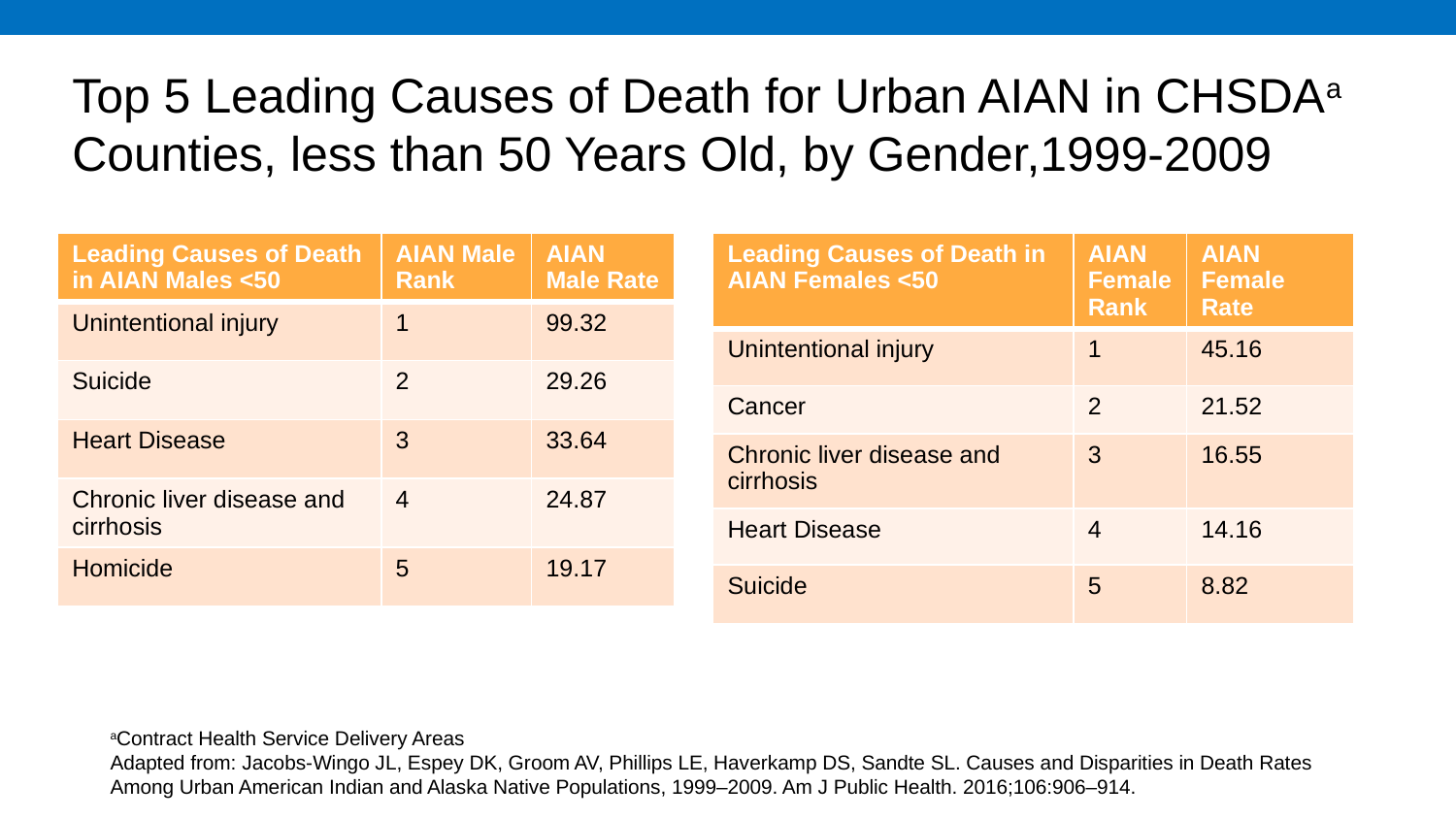

# Top 5 Leading Causes of Death for Urban AIAN in CHSDAa Counties, less than 50 Years Old, by Gender,1999-2009
| Leading Causes of Death in AIAN Males <50 | AIAN Male Rank | AIAN Male Rate |
| --- | --- | --- |
| Unintentional injury | 1 | 99.32 |
| Suicide | 2 | 29.26 |
| Heart Disease | 3 | 33.64 |
| Chronic liver disease and cirrhosis | 4 | 24.87 |
| Homicide | 5 | 19.17 |
| Leading Causes of Death in AIAN Females <50 | AIAN Female Rank | AIAN Female Rate |
| --- | --- | --- |
| Unintentional injury | 1 | 45.16 |
| Cancer | 2 | 21.52 |
| Chronic liver disease and cirrhosis | 3 | 16.55 |
| Heart Disease | 4 | 14.16 |
| Suicide | 5 | 8.82 |
aContract Health Service Delivery Areas
Adapted from: Jacobs-Wingo JL, Espey DK, Groom AV, Phillips LE, Haverkamp DS, Sandte SL. Causes and Disparities in Death Rates Among Urban American Indian and Alaska Native Populations, 1999–2009. Am J Public Health. 2016;106:906–914.

## Slide 31
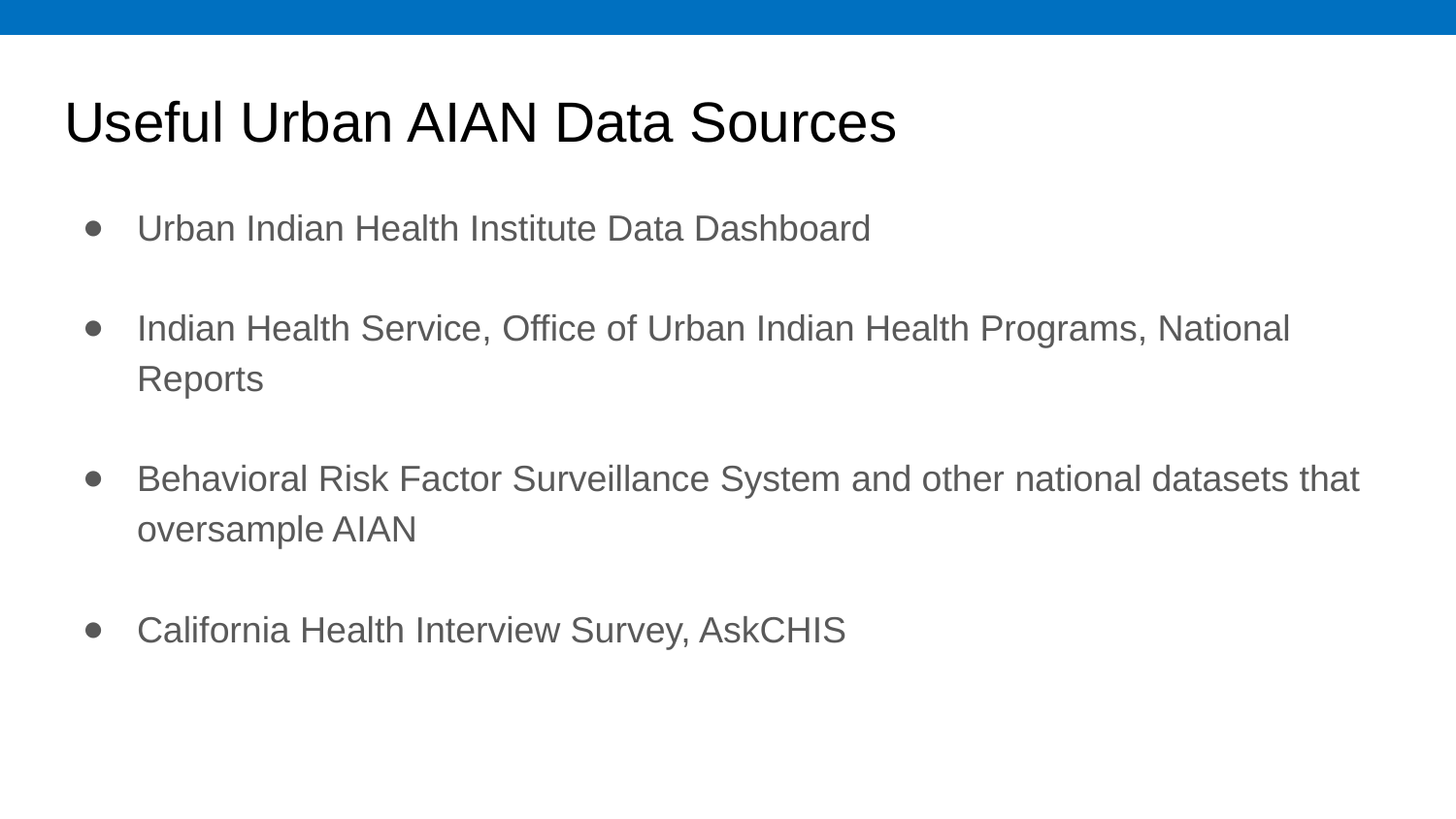

# Useful Urban AIAN Data Sources
Urban Indian Health Institute Data Dashboard
Indian Health Service, Office of Urban Indian Health Programs, National Reports
Behavioral Risk Factor Surveillance System and other national datasets that oversample AIAN
California Health Interview Survey, AskCHIS

## Slide 32
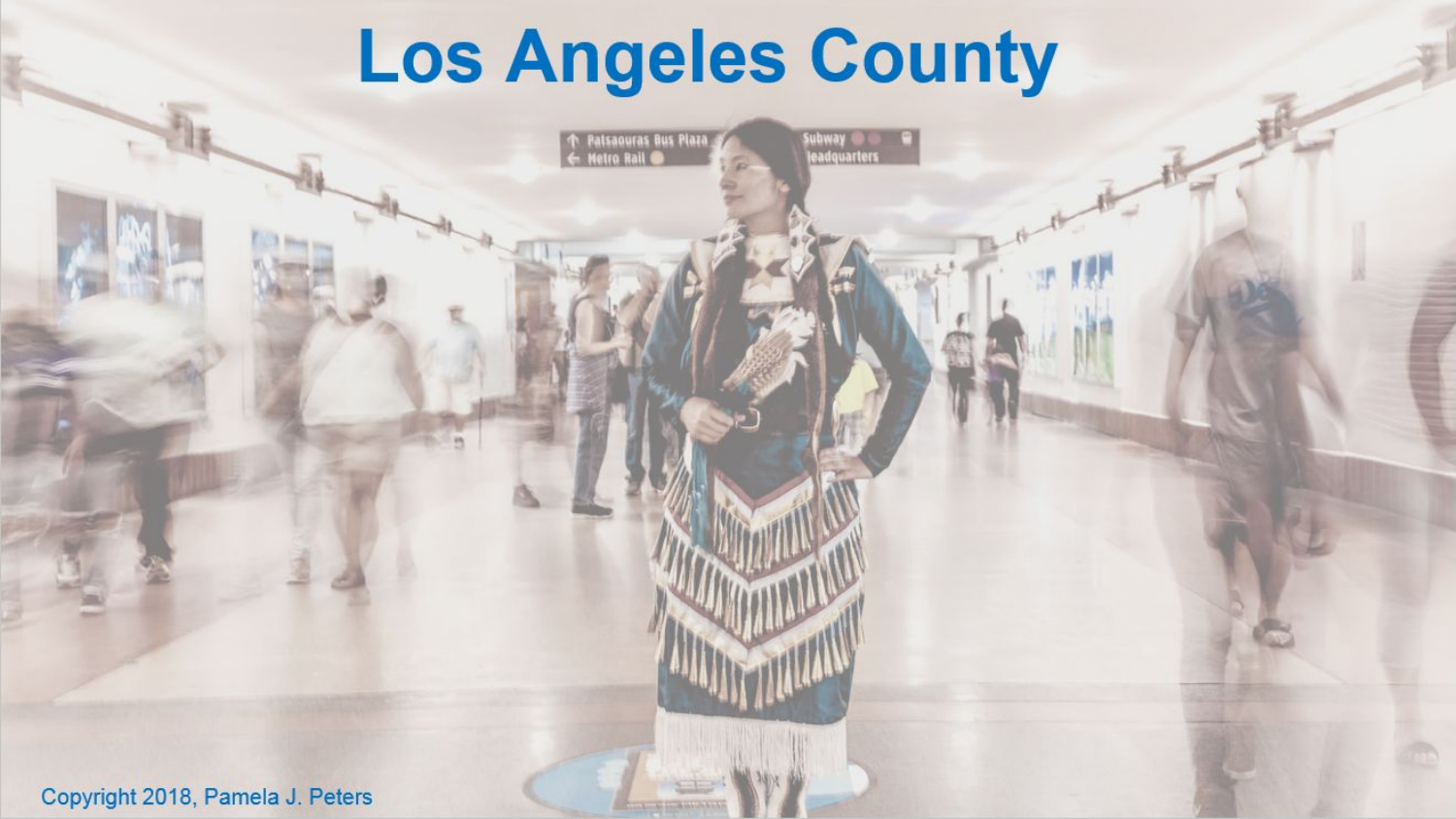

## Slide 33
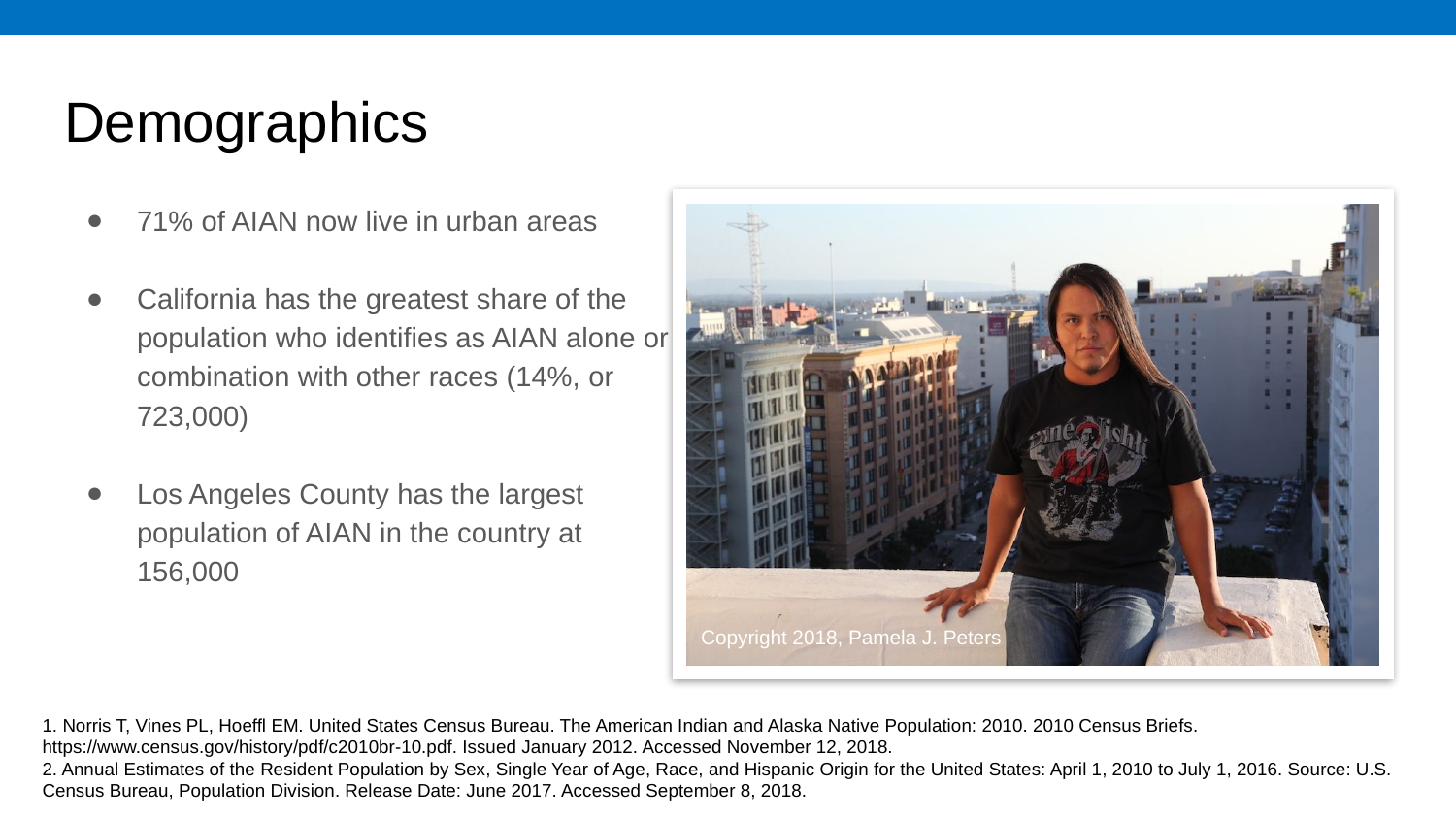

# Demographics
71% of AIAN now live in urban areas
California has the greatest share of the population who identifies as AIAN alone or combination with other races (14%, or 723,000)
Los Angeles County has the largest population of AIAN in the country at 156,000
Copyright 2018, Pamela J. Peters
1. Norris T, Vines PL, Hoeffl EM. United States Census Bureau. The American Indian and Alaska Native Population: 2010. 2010 Census Briefs. https://www.census.gov/history/pdf/c2010br-10.pdf. Issued January 2012. Accessed November 12, 2018.
2. Annual Estimates of the Resident Population by Sex, Single Year of Age, Race, and Hispanic Origin for the United States: April 1, 2010 to July 1, 2016. Source: U.S. Census Bureau, Population Division. Release Date: June 2017. Accessed September 8, 2018.

## Slide 34
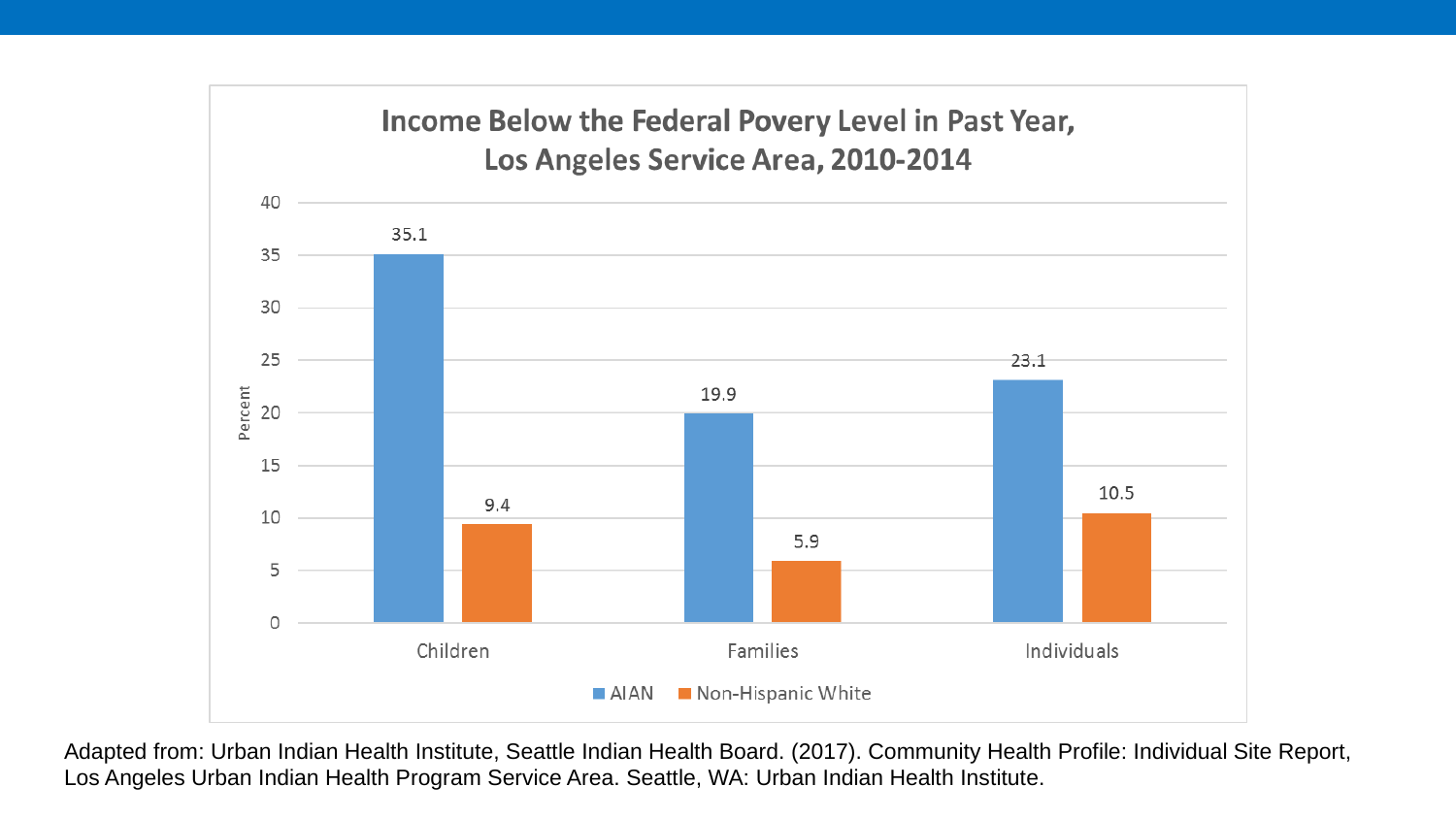

Adapted from: Urban Indian Health Institute, Seattle Indian Health Board. (2017). Community Health Profile: Individual Site Report, Los Angeles Urban Indian Health Program Service Area. Seattle, WA: Urban Indian Health Institute.

## Slide 35
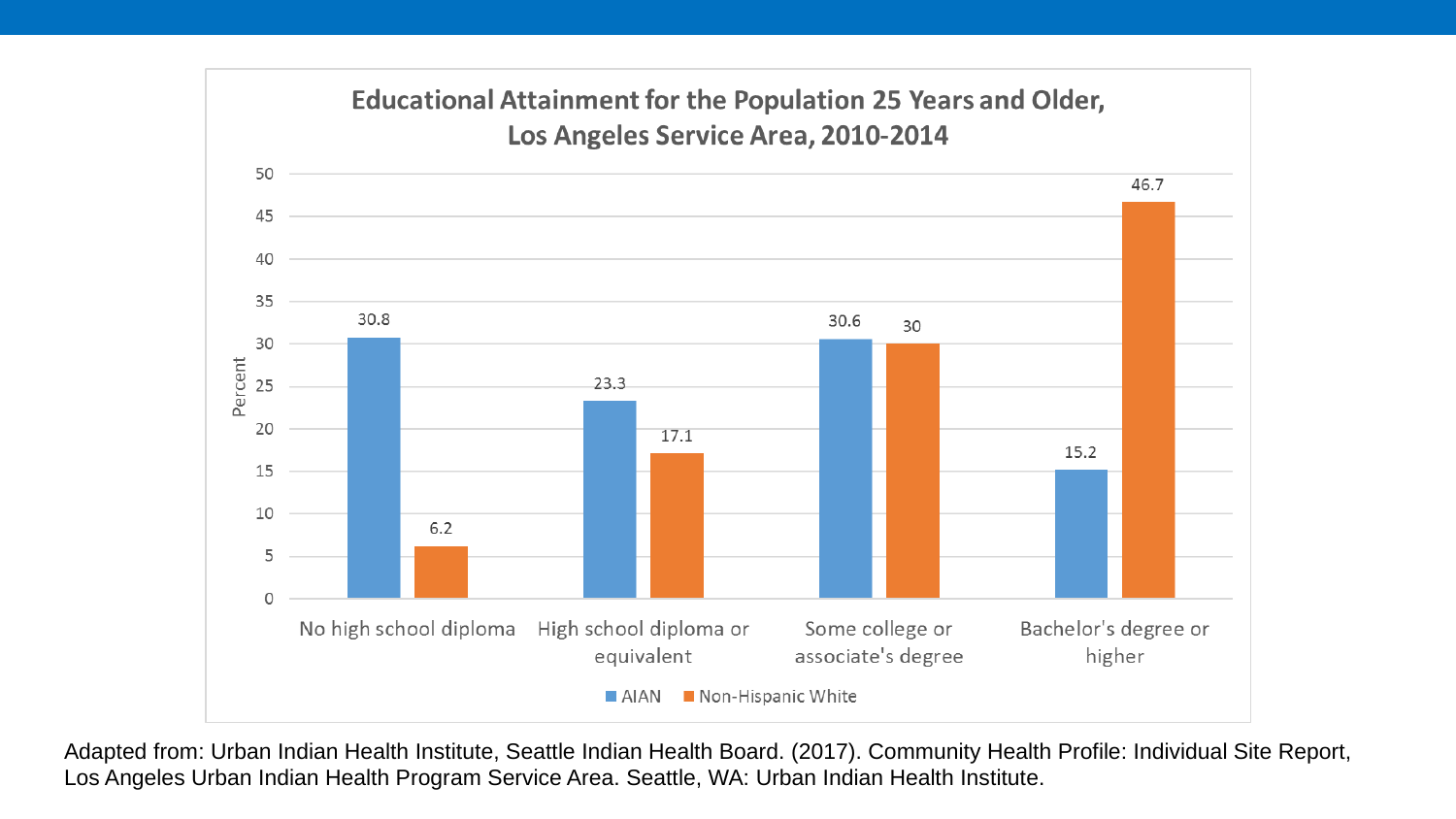

Adapted from: Urban Indian Health Institute, Seattle Indian Health Board. (2017). Community Health Profile: Individual Site Report, Los Angeles Urban Indian Health Program Service Area. Seattle, WA: Urban Indian Health Institute.

## Slide 36
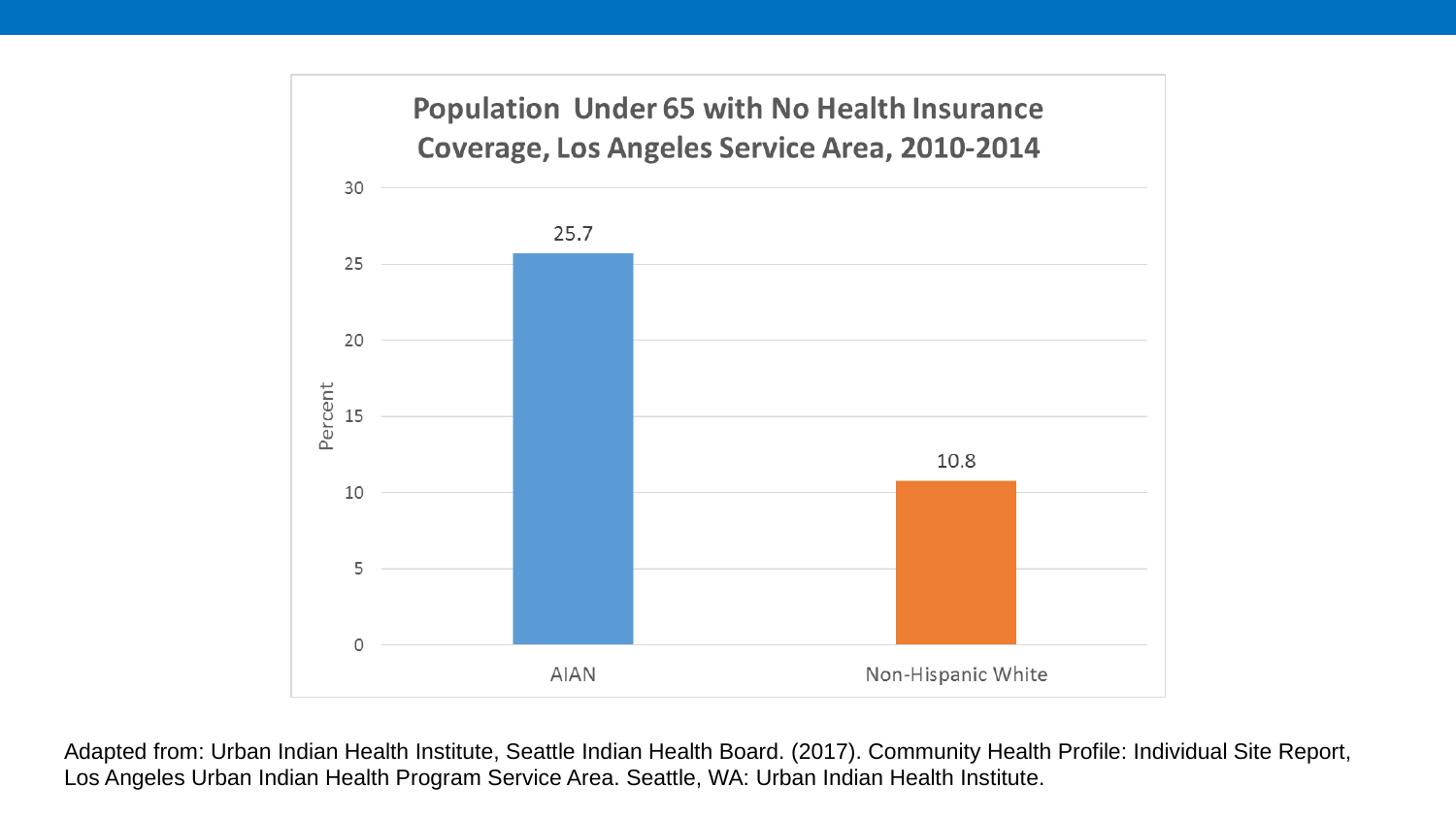

Adapted from: Urban Indian Health Institute, Seattle Indian Health Board. (2017). Community Health Profile: Individual Site Report, Los Angeles Urban Indian Health Program Service Area. Seattle, WA: Urban Indian Health Institute.

## Slide 37
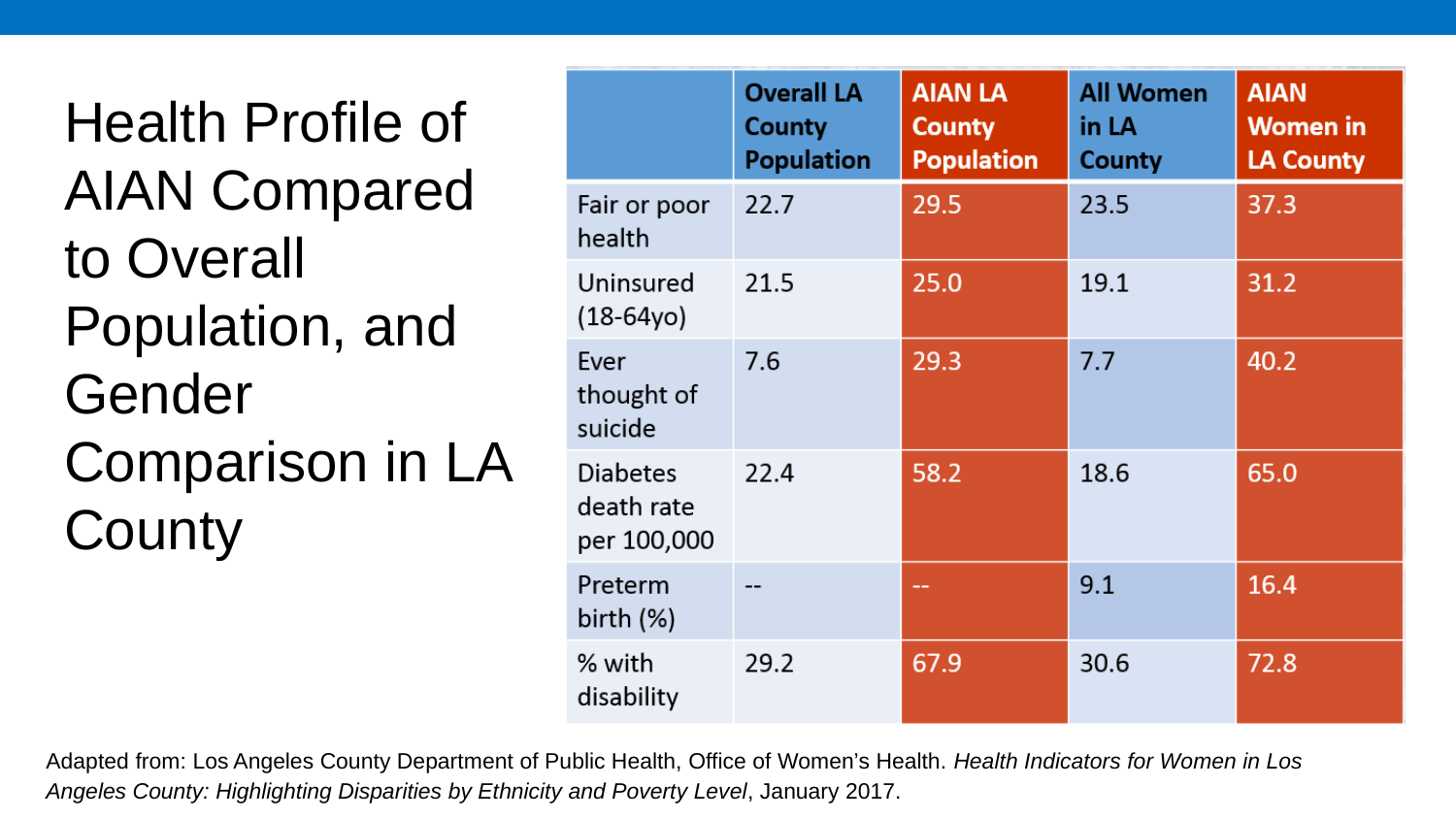

# Health Profile of AIAN Compared to Overall Population, and Gender Comparison in LA County
Adapted from: Los Angeles County Department of Public Health, Office of Women’s Health. Health Indicators for Women in Los Angeles County: Highlighting Disparities by Ethnicity and Poverty Level, January 2017.

## Slide 38
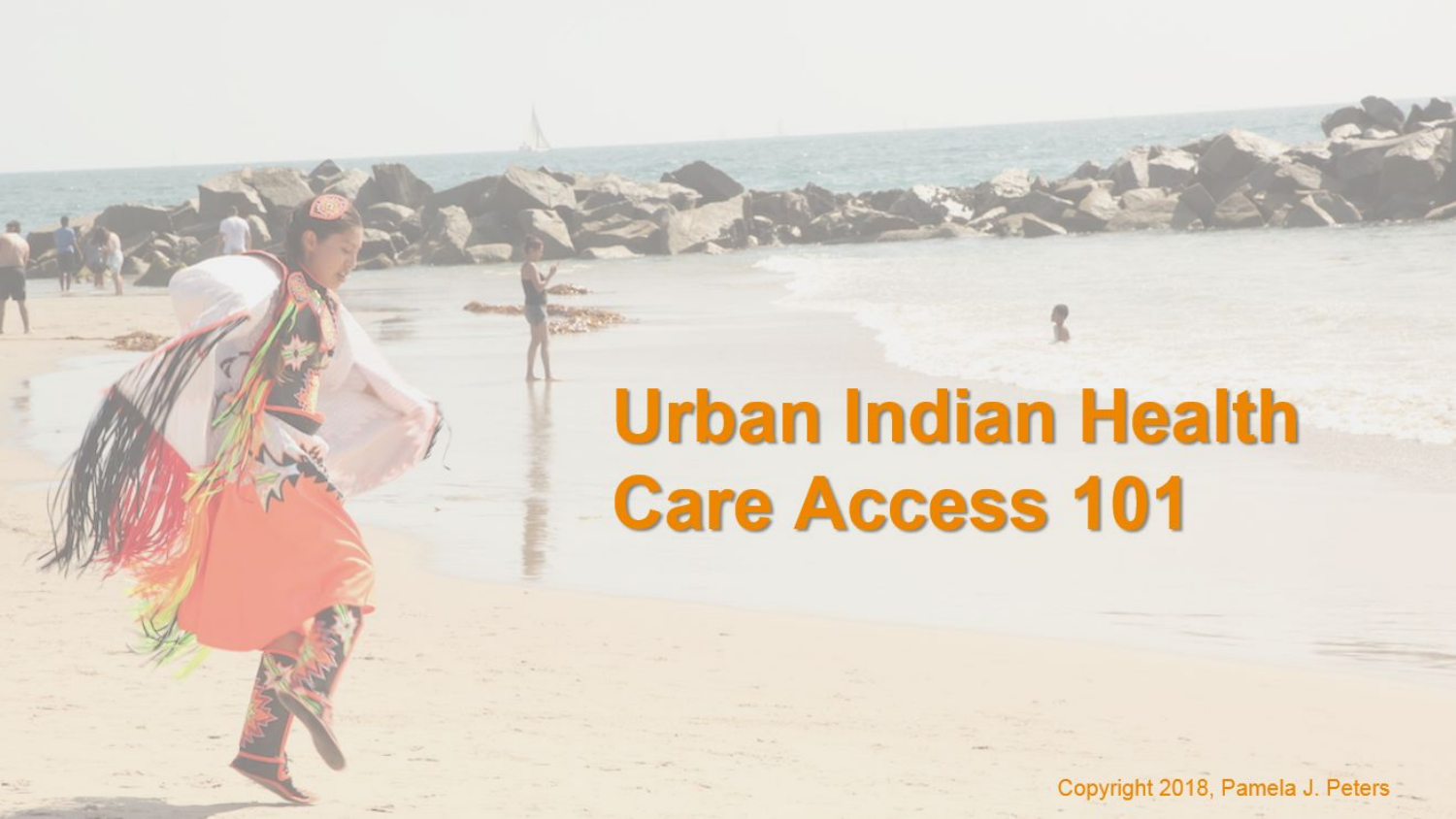

## Slide 39
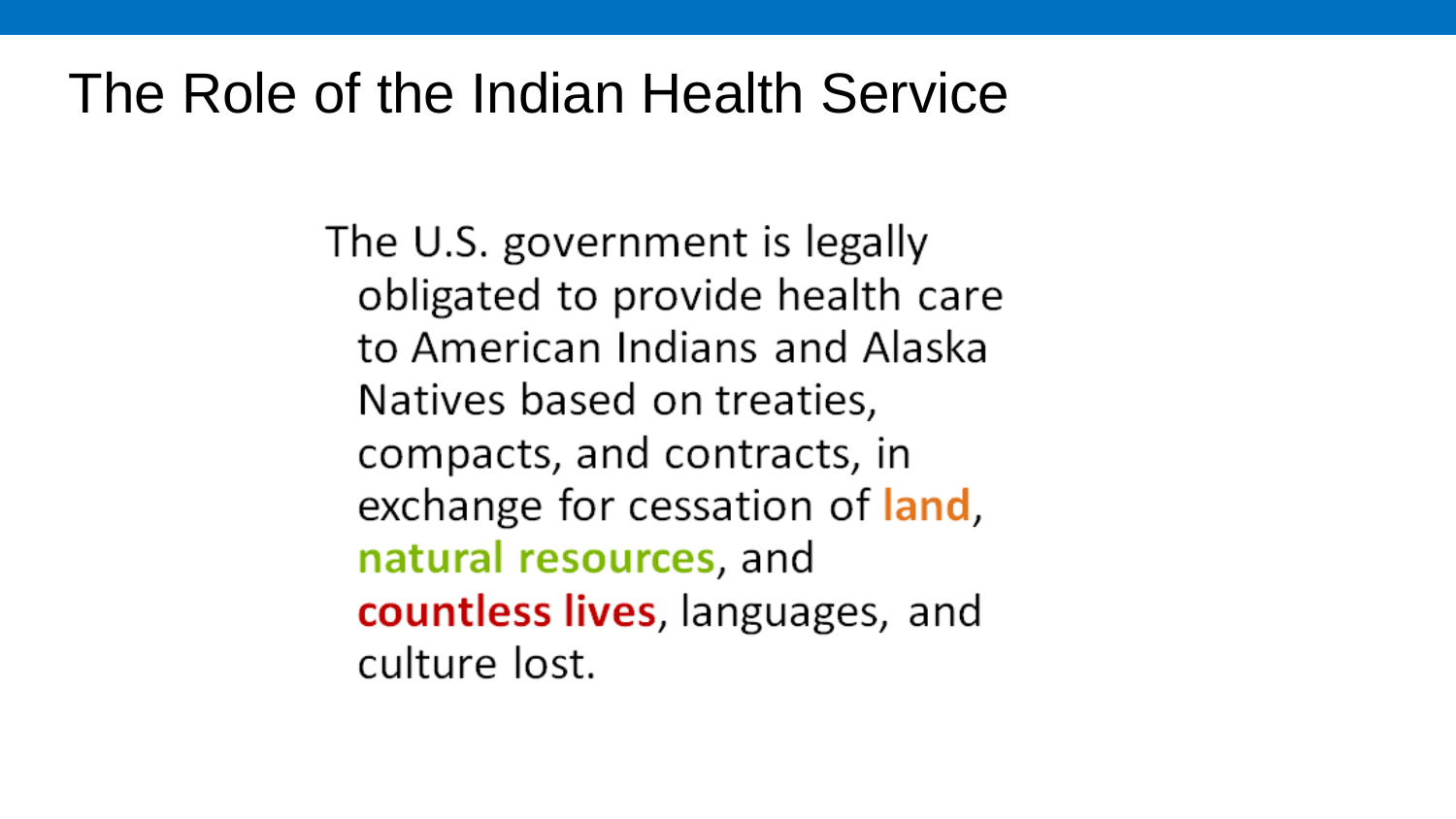

The Role of the Indian Health Service

## Slide 40
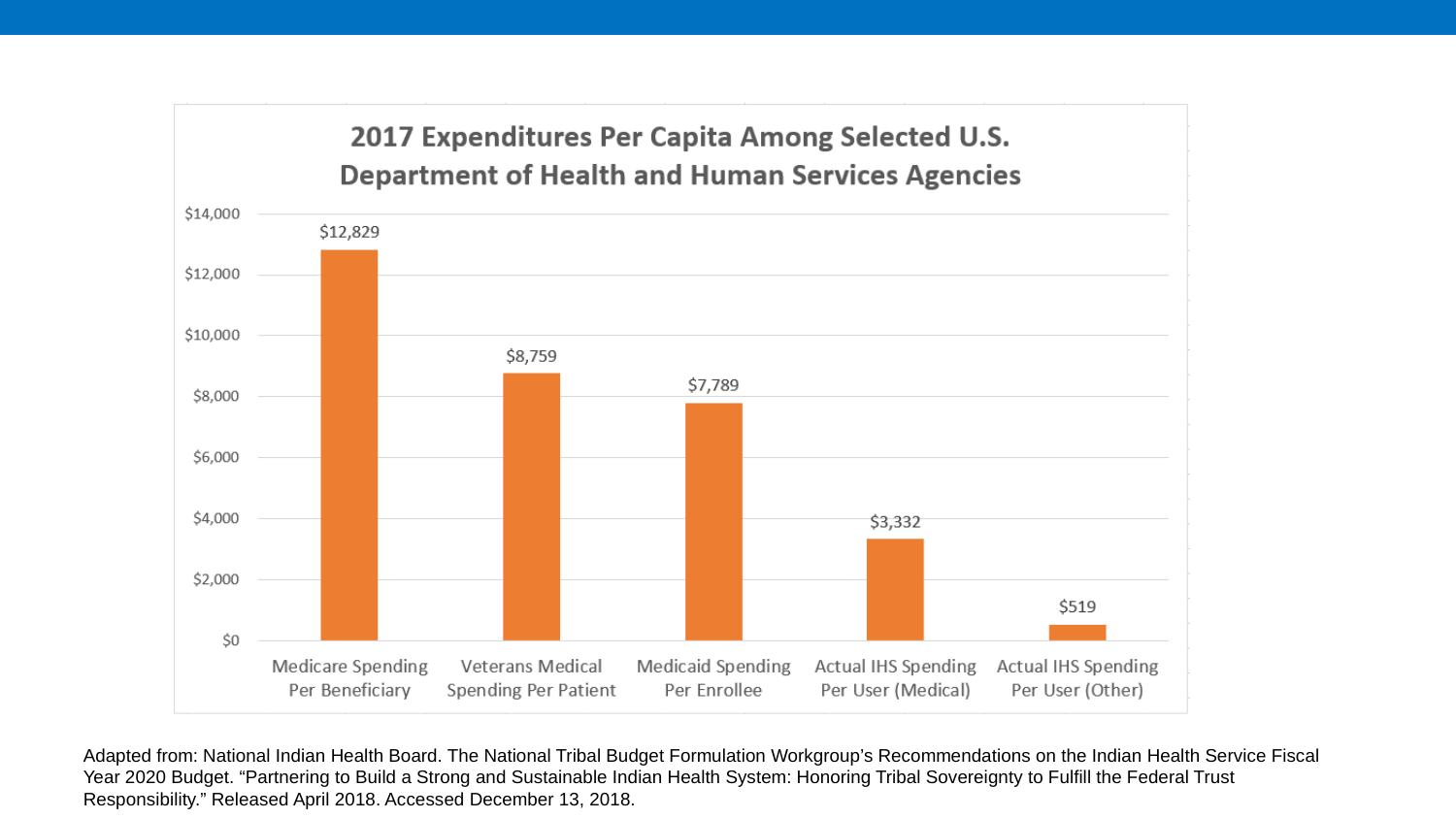

Adapted from: National Indian Health Board. The National Tribal Budget Formulation Workgroup’s Recommendations on the Indian Health Service Fiscal Year 2020 Budget. “Partnering to Build a Strong and Sustainable Indian Health System: Honoring Tribal Sovereignty to Fulfill the Federal Trust Responsibility.” Released April 2018. Accessed December 13, 2018.

## Slide 41
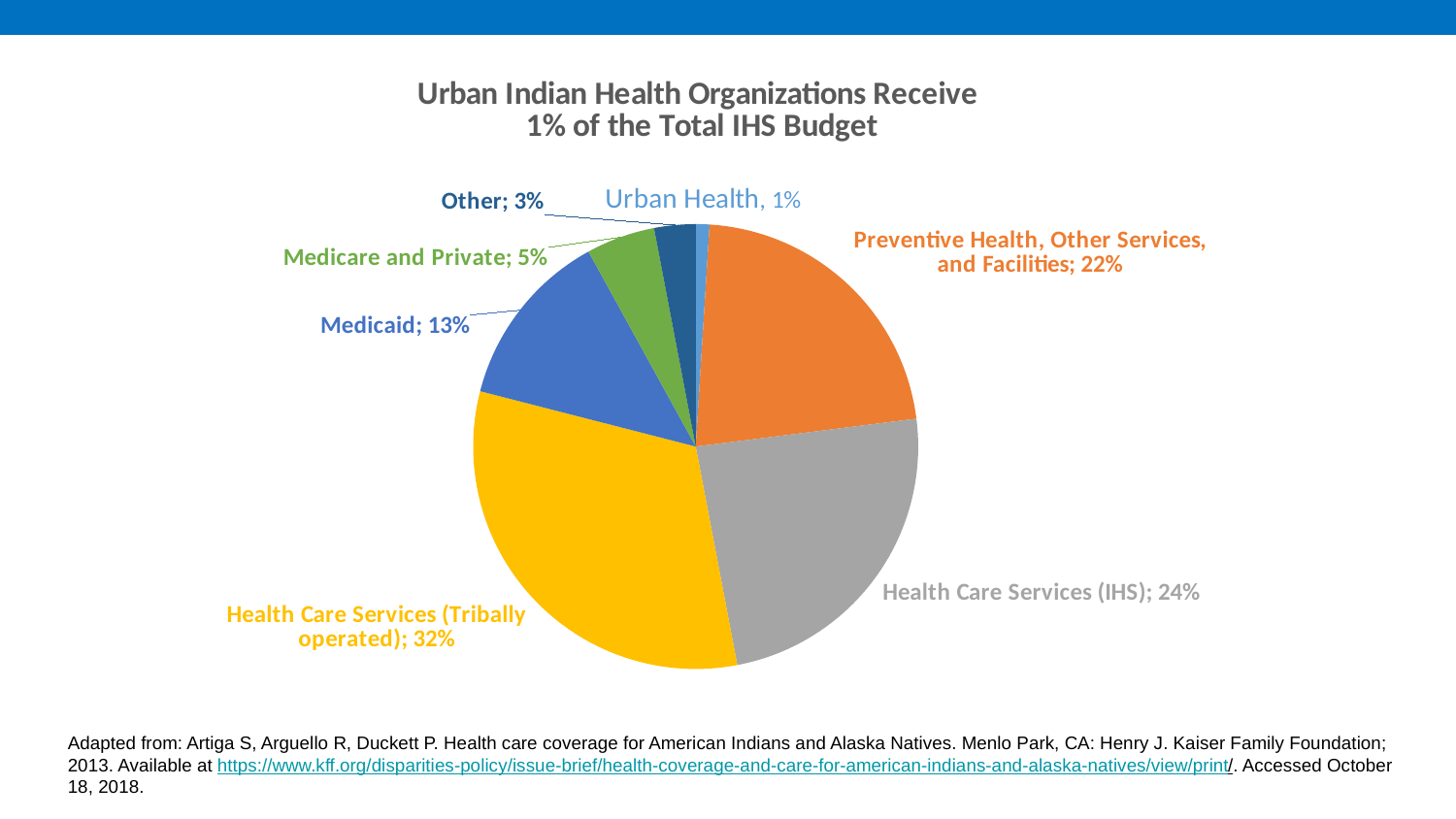

[unsupported chart]
Adapted from: Artiga S, Arguello R, Duckett P. Health care coverage for American Indians and Alaska Natives. Menlo Park, CA: Henry J. Kaiser Family Foundation; 2013. Available at https://www.kff.org/disparities-policy/issue-brief/health-coverage-and-care-for-american-indians-and-alaska-natives/view/print/. Accessed October 18, 2018.

## Slide 42
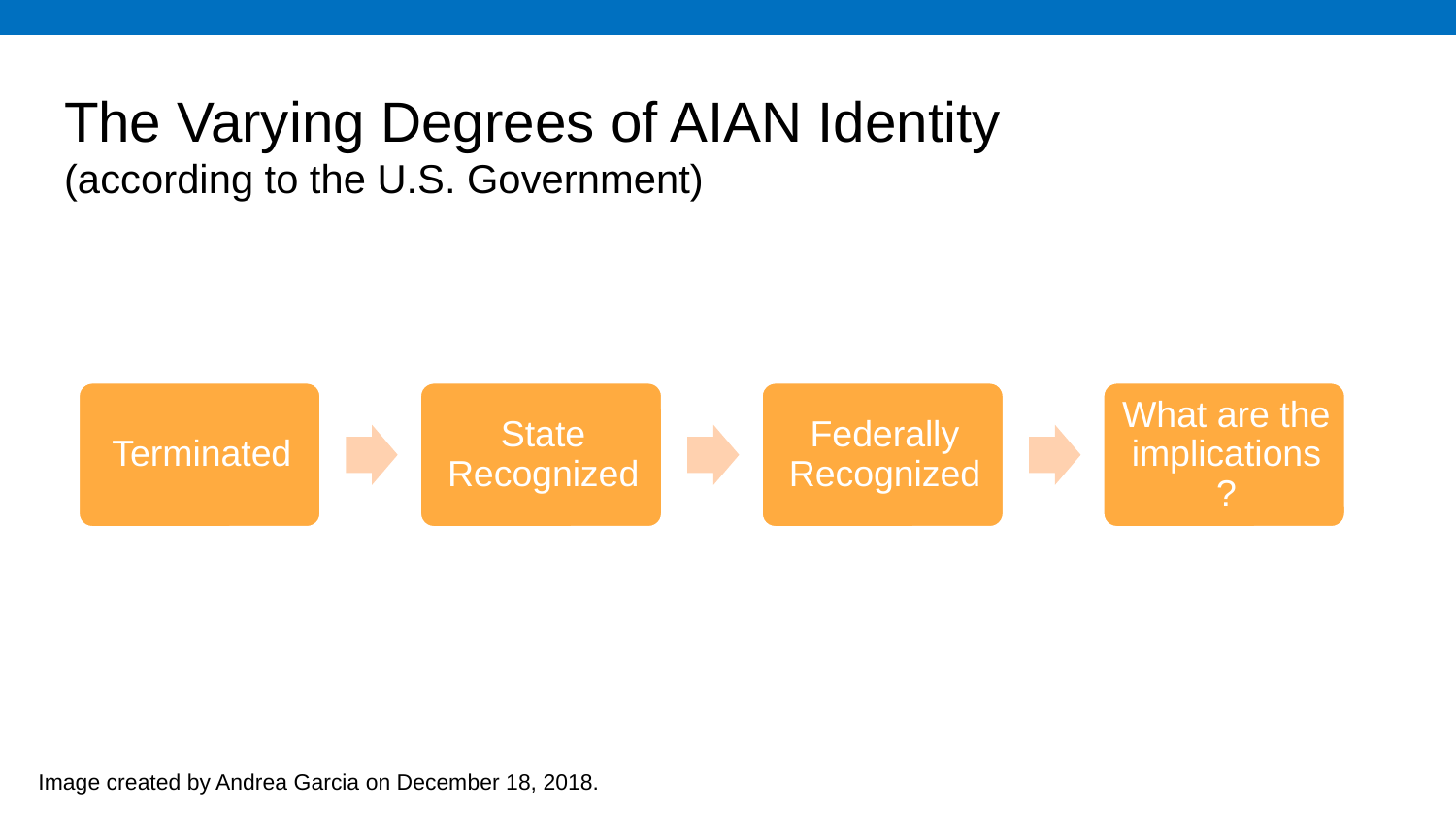

# The Varying Degrees of AIAN Identity (according to the U.S. Government)
Image created by Andrea Garcia on December 18, 2018.

## Slide 43
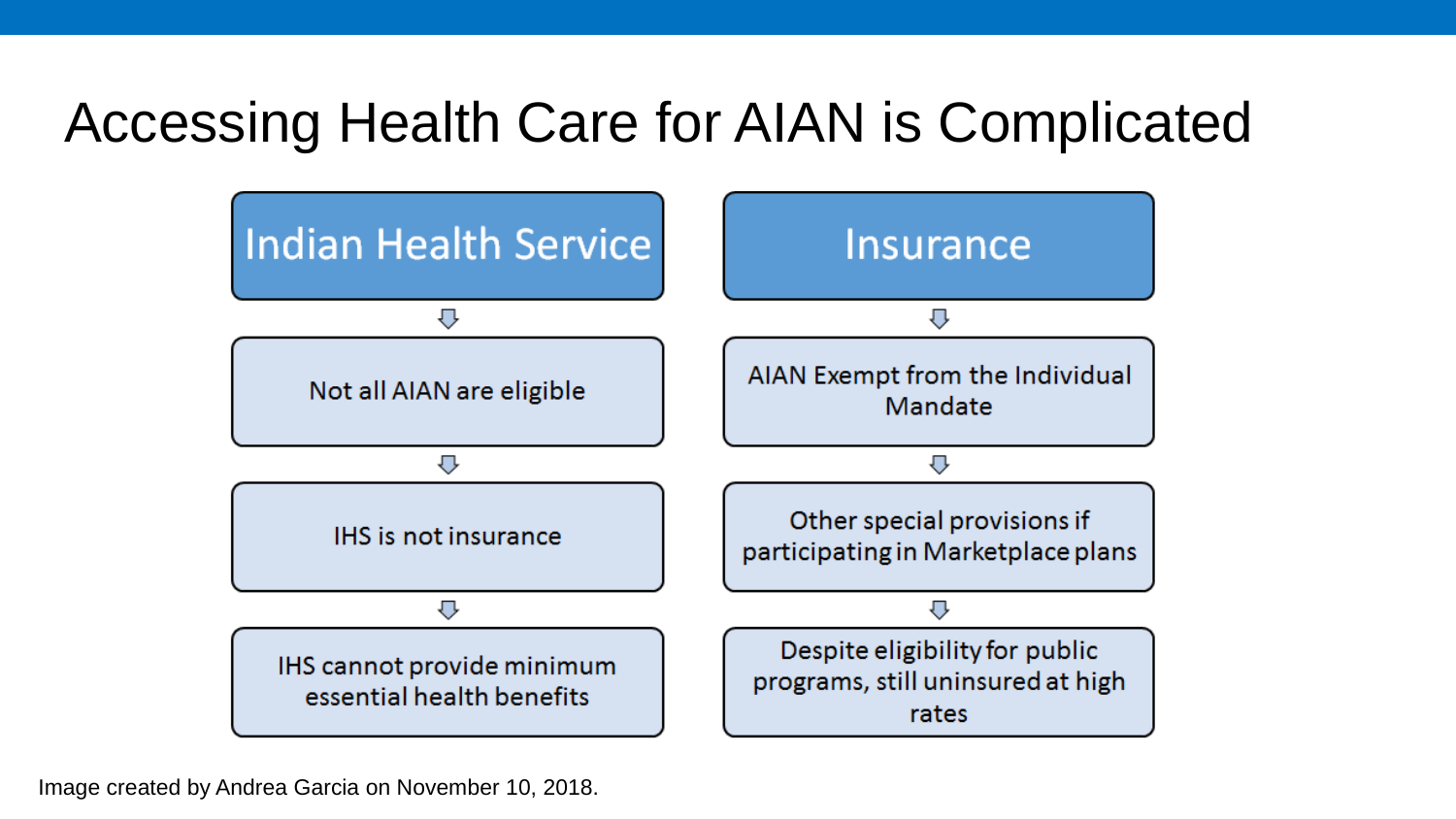

# Accessing Health Care for AIAN is Complicated
Image created by Andrea Garcia on November 10, 2018.

## Slide 44
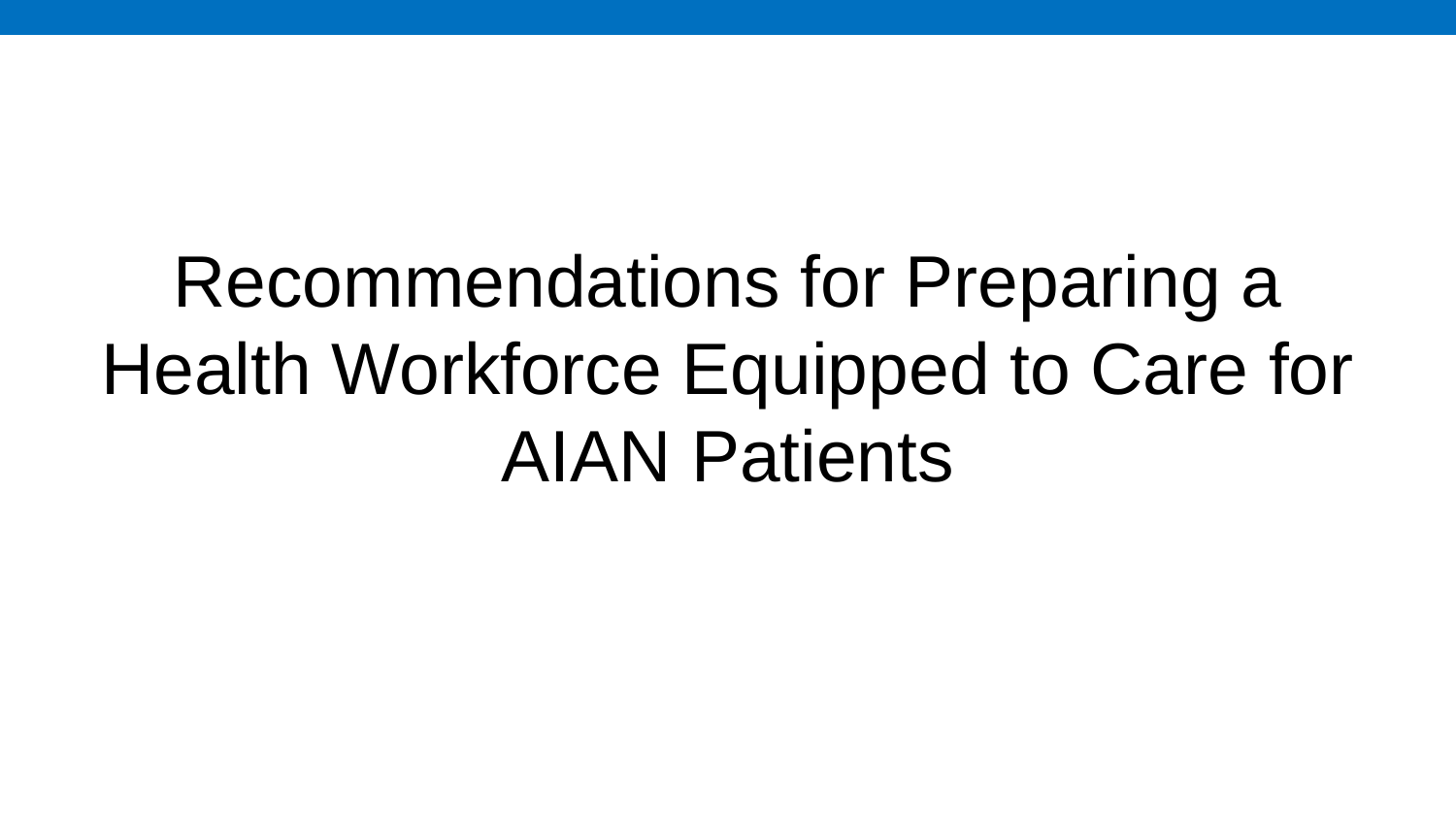

# Recommendations for Preparing a Health Workforce Equipped to Care for AIAN Patients

## Slide 45
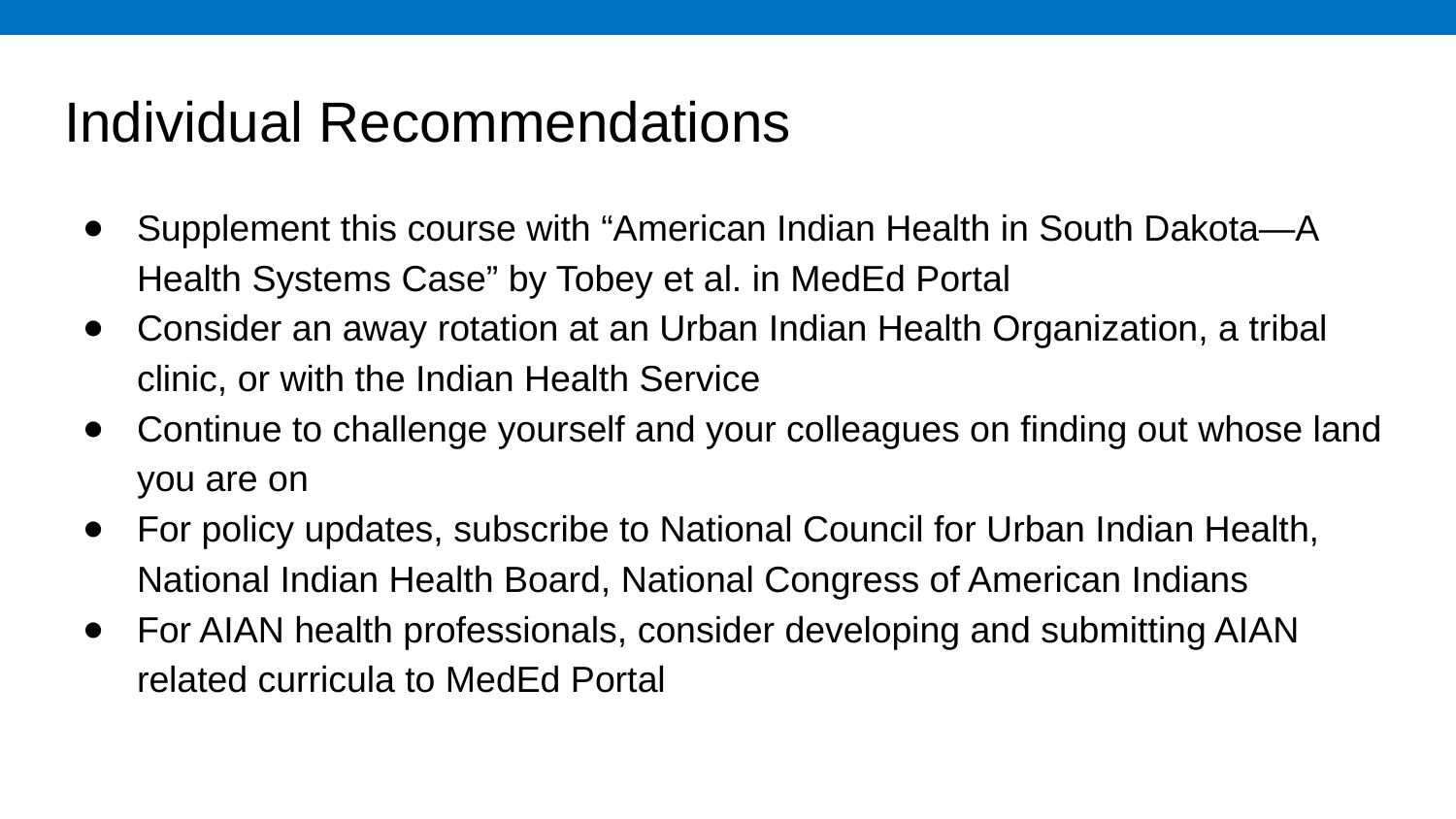

# Individual Recommendations
Supplement this course with “American Indian Health in South Dakota—A Health Systems Case” by Tobey et al. in MedEd Portal
Consider an away rotation at an Urban Indian Health Organization, a tribal clinic, or with the Indian Health Service
Continue to challenge yourself and your colleagues on finding out whose land you are on
For policy updates, subscribe to National Council for Urban Indian Health, National Indian Health Board, National Congress of American Indians
For AIAN health professionals, consider developing and submitting AIAN related curricula to MedEd Portal

## Slide 46
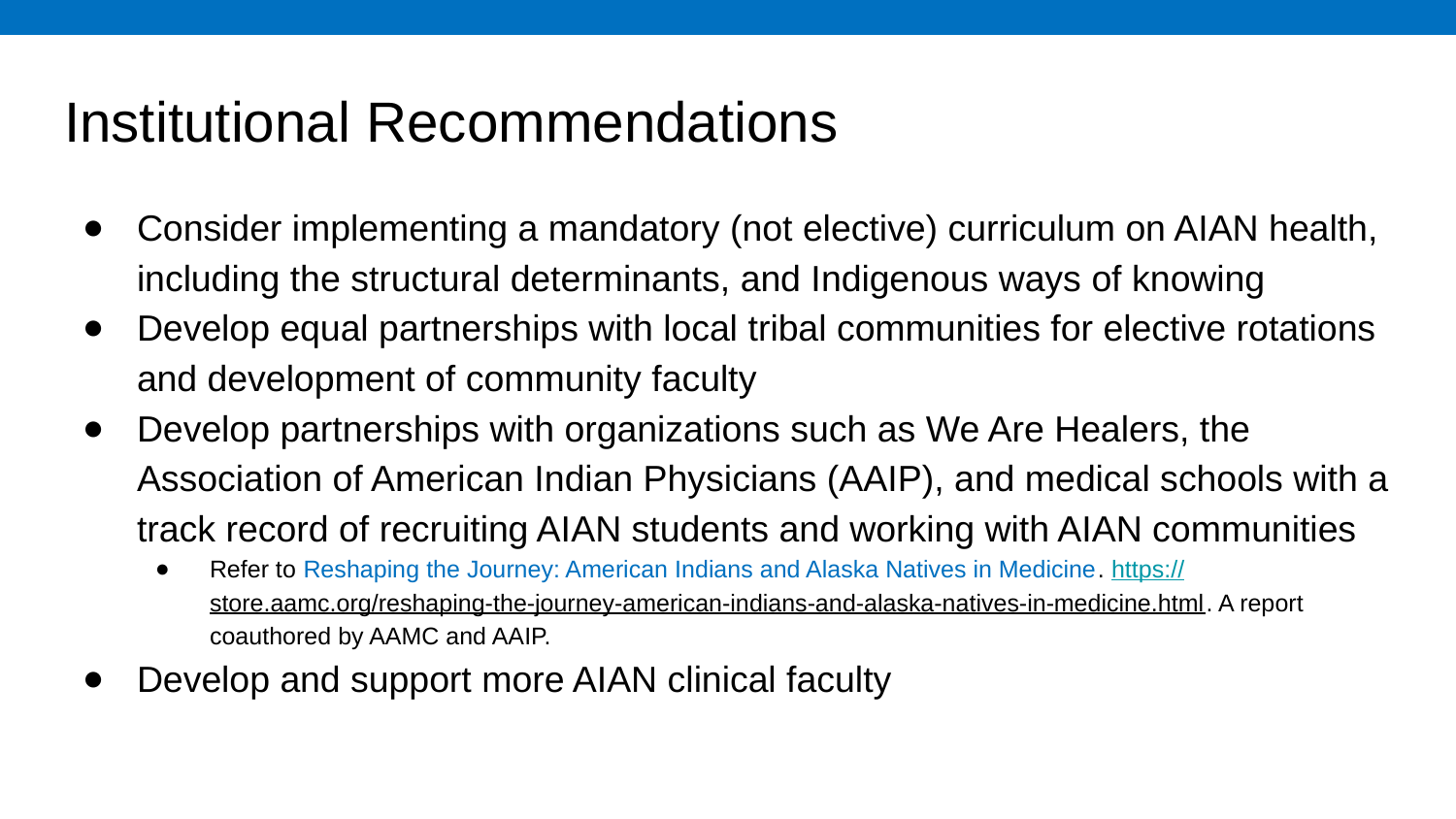

# Institutional Recommendations
Consider implementing a mandatory (not elective) curriculum on AIAN health, including the structural determinants, and Indigenous ways of knowing
Develop equal partnerships with local tribal communities for elective rotations and development of community faculty
Develop partnerships with organizations such as We Are Healers, the Association of American Indian Physicians (AAIP), and medical schools with a track record of recruiting AIAN students and working with AIAN communities
Refer to Reshaping the Journey: American Indians and Alaska Natives in Medicine. https://store.aamc.org/reshaping-the-journey-american-indians-and-alaska-natives-in-medicine.html. A report coauthored by AAMC and AAIP.
Develop and support more AIAN clinical faculty

## Slide 47
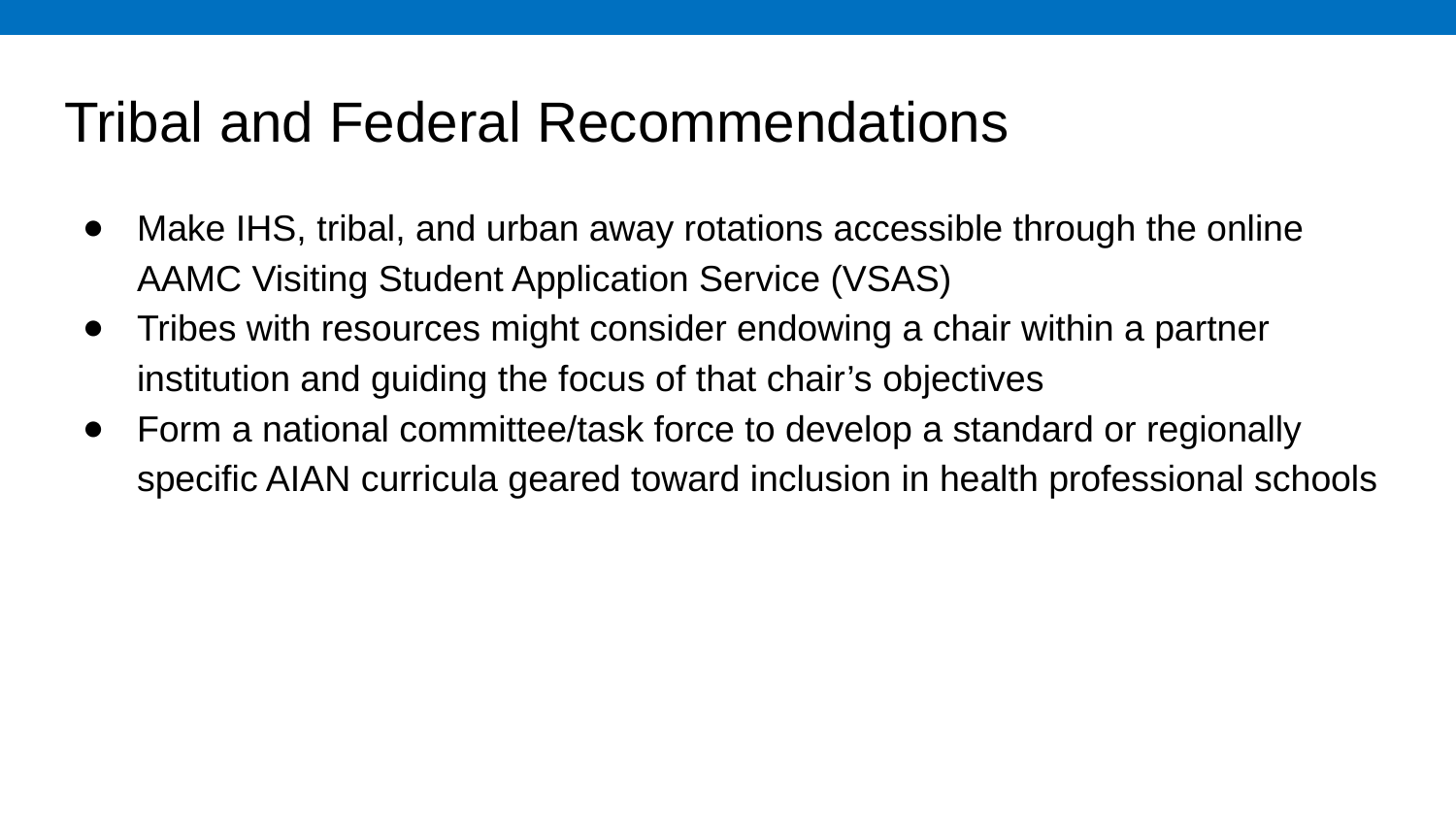

# Tribal and Federal Recommendations
Make IHS, tribal, and urban away rotations accessible through the online AAMC Visiting Student Application Service (VSAS)
Tribes with resources might consider endowing a chair within a partner institution and guiding the focus of that chair’s objectives
Form a national committee/task force to develop a standard or regionally specific AIAN curricula geared toward inclusion in health professional schools

## Slide 48
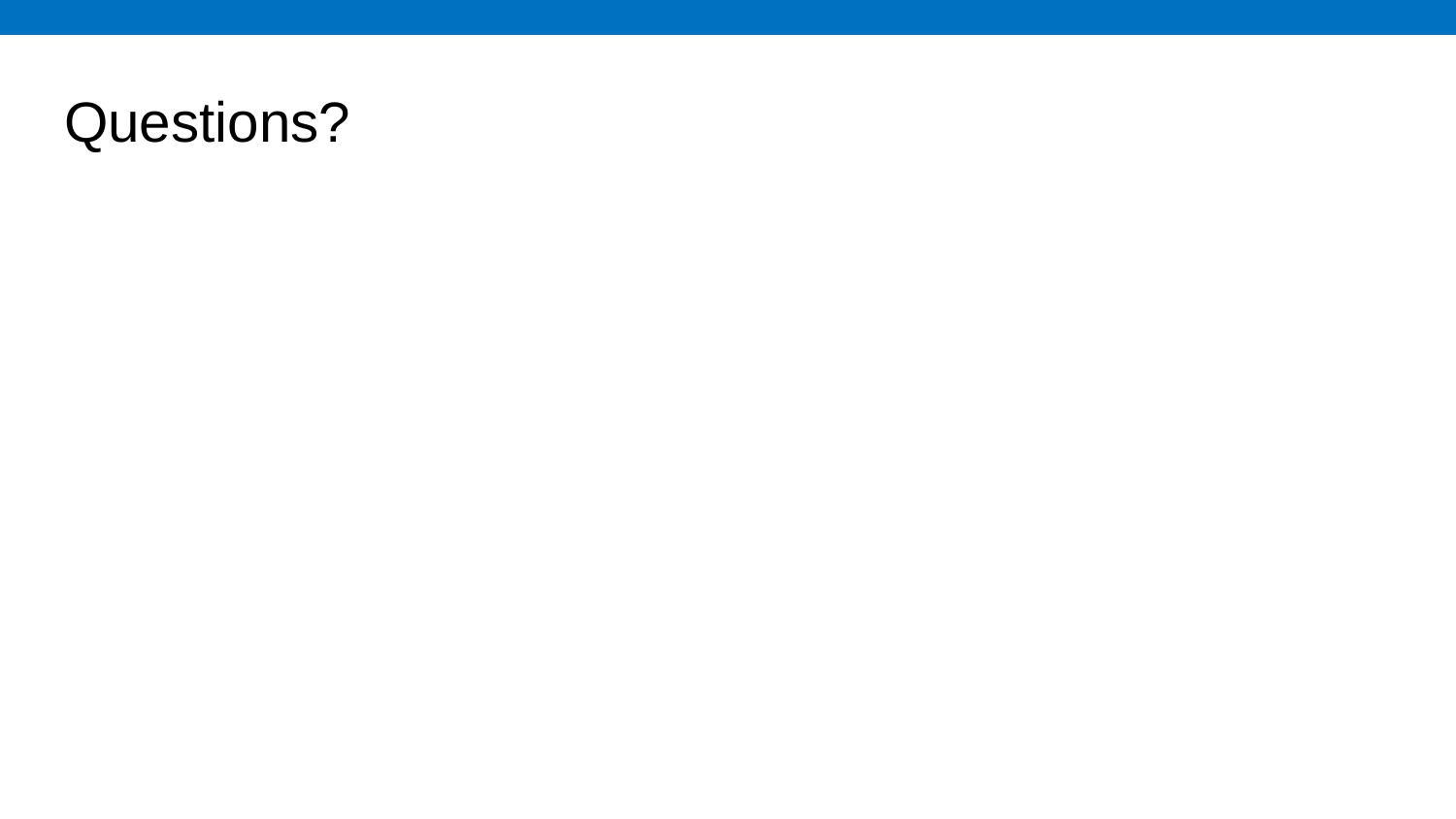

# Questions?
