# Supplementary material for: Social and Structural Determinants of Urban American Indian and Alaska Native Health: A Case Study in Los Angeles
Source: MedEdPORTAL. 2019 May 15;15:10825. doi: 10.15766/mep_2374-8265.10825 (PMC6543927; doi:10.15766/mep_2374-8265.10825)
Supplement: Supplementary file 1 — A. PowerPoint Presentation.pptx B. Facilitator Guide.docx C. Video Honor Native Land.mp4 D. Video The Art of Indigenous Resistance.mp4 E. Evaluation Form.pdf [file mep-15-10825-s001.zip › B. Facilitator Guide.docx]

**The Social and Structural Determinants of Urban American Indian and**

**Alaska Native Health**

**Facilitator Instructional Guide**

**Overall Goals**

The goals of this module are to help medical students, residents, and other health professionals describe and understand social and structural determinants of health as they pertain specifically to urban American Indian and Alaska Native communities. Please note that this module is designed to be implemented by individuals from any background.

**Workshop Objectives**

1. Describe the importance of and how to acknowledge the tribal homelands on which you stand
2. Describe how at least two Federal Indian policies have impacted the demographics and health outcomes of AIAN in urban areas
3. Explain how AIAN identity may be associated with access to health care

**Workshop Handouts and Materials**

- Story-Telling Cards
- Evaluation Form
- Computer set up with audio capability, and connection to projector
- Video: Honor Native Land
- Video: The Art of Indigenous Resistance

**Suggested Agenda and Timeline**

- Pre-workshop evaluation: 5 minutes.
- Slide 1-4, Land Acknowledgement: 10 minutes
- Slide 5-13, Introduction of objectives, and video discussion: 15 minutes
- Slide 14-26, Storytelling exercise: 25 minutes
- Slide 27-47, Reinforcing policies, Los Angeles Case Study, Urban Indian Health Care, Recommendations: 20 minutes
- Slide 48: Questions and Answers: 10 minutes
- Post-workshop evaluation: 5 minutes

**Slide Instructions**

*Speakers may wish to update the statistics listed in the presentation, depending on when/where this module is implemented. If this is the case, we suggest visiting the Urban Indian Health Institute’s “Community Health Profiles” or “Data Dashboard” for information about specific urban communities.^1^ We also suggest visiting the Indian Health Service website (https://www.ihs.gov/), and perhaps completing a PubMed search for potentially recent publications. That being said, this module uses Los Angeles as a case study because it is the largest population of AIAN in the United States.*

**Slide 1**:

*The facilitators should introduce themselves to the audience and discuss their roles in their respective institutions. Their background information (institution, degrees, etc.) should be added to the slide. If desired, those presenters who have a tribal affiliation are invited to share this with the group. For those facilitators who do not identify as American Indian/Alaska Native, we suggest you also acknowledge this fact. In both cases, facilitators should acknowledge that American Indians/Alaska Natives are a diverse group of people, with diverse histories, and there is no one ‘expert’ capable of knowing all the nuances of AIAN experiences. The facilitator, regardless of identity, should make it a point to say they approach this content with humility, and that there is much more content to be written. We suggest facilitators acknowledge that they are following the content and direction of this peer-reviewed module, and further resources will be shared for outstanding questions that may not be answered here. At this point, facilitators should set the stage for the interchangeable terminology that will be used throughout the presentation.*

Native American, American Indian/Alaska Native, “Native” will be used interchangeably throughout this presentation. All of these terms denote that AIAN are more than just a racial/ethnic group, but have a unique political status with the United States government. Federally recognized tribes are sovereign nations, and therefore a have a nation-to-nation relationship with the federal government. We’ll explain in more detail later in the presentation.

**Slide 2:**

*Before the start of the presentation, the facilitators should refer to* https://native-land.ca/ *to identify the tribal homelands on which the presentation is taking place. You simply click on the link, and enter either a zip code or city. You may zoom in or out of the map accordingly. It’s possible to take a screenshot of the map and paste onto the corresponding slide. Then, on the left side of the page you will see a box that says, “You are on the land of: [with the name and hyperlink to a tribe]. That link provides further information about the tribe, perhaps a tribal seal, and perhaps another map. You may also wish to paste the tribal seal onto the ‘Land Acknowledgement’ slide, slide 2, prior to the presentation. You may also wish to read more about the tribe in case any questions arise. Suggested dialogue follows:*

Is there anyone in the room who identifies as Tongva/Yaavitam? (*this is subject to change based on location of presentation*)?

*(If yes, please invite the individual to provide a welcome or provide comments about the traditional homelands)*

*If no one identifies themselves, suggested dialogue follows:* We respectfully acknowledge the [insert appropriate tribal name] people, whose original homelands we stand on today. We acknowledge their elders, past, present, and future for being stewards of this land, and we acknowledge their cultural resilience.

**Slide 3:**

Why are we talking about land acknowledgement, and what does it have to do with health? We’ll watch this brief video and come back to discuss.

*Facilitator then clicks on link to play video.^2^ Suggested comments follow:*

Why is it important to do a land acknowledgement?

**It is important to do a land acknowledgement because it recognizes the original caretakers of this land**, their contributions, perseverance, and existence, not just from the past, but today, and the future. It is the beginning of a larger movement of truth and reconciliation for Indigenous peoples.

**Slide 4:**

Whether you are an undergraduate, a medical student, resident, faculty member, or Dean, we challenge you to do a Land Acknowledgement whenever you have a public presentation.

You can use the link https://native-land.ca to find the original homelands of the Indigenous Peoples on which you are residing, teaching, learning, and or visiting.^3^ You can also use the app, “Native Land,” on your iPhone or Android. *Facilitator should click on the link and then ask someone from the audience to provide them with a zip code or city. Facilitator will enter the zip code and demonstrate how the website works. Also note that this map is a work in progress. Please contact the website owner if you find errors. More suggested dialogue below.*

Please note, original Native homelands/territories and reservation lands are completely different. This map is intended to show the original Native territories prior to colonization.

Disclaimer shared by the website: This map does not represent or intend to represent official or legal boundaries of any Indigenous nations. To learn about definitive boundaries, contact the nations in question.

**Slide 5:**

*The facilitators should state learning objectives for the workshop.*

The objectives for today’s presentation are the following:

1. Describe the importance of and how to acknowledge the tribal homelands on which you stand—By now, we should have succeeded at that objective
2. Describe how at least two Federal Indian policies have impacted the demographics and health outcomes of AIAN in urban areas
3. Explain how AIAN identity may be associated with access to health care

**Slide 6:**

*Please read the Agenda verbatim*

1. A video introduction and discussion about the social and structural determinants of health
2. The use of storytelling to connect Federal Indian policy and AIAN health
3. Urban American Indians/Alaska Natives (AIAN): Los Angeles as an example
4. Recommendations for preparing a health workforce equipped to care for AIAN patients

**Slide 7:**

*This is a transitional slide. Please read the section header to indicate the next activity.*

**Slide 8:**

*Suggested dialogue can be the following:*

We are going to watch a video of an event that took place in Los Angeles. The purpose of this video is to give you an example of, and set the stage for a discussion about the social and structural determinants of AIAN health. *Please read the prompts/questions verbatim:*

1. Please take note of the demographic diversity of the participants.
2. What are some of the leading health issues that are depicted in this video? Why might these health issues exist?
3. What are some of the ways that this community raises awareness about these issues?

**Slide 9:**

*Facilitator will click on link and show video.*

**Slide 10:**

*Facilitator will lead discussion based on the listed questions. Potential discussion points/answers are displayed below:*

| **Discussion Questions** | **Potential Discussion Points** |
| --- | --- |
| Please take note of the demographic diversity of the participants. | Young people; women; families with young children or babies (intergenerational); indigenous people from all over the world represented; artists; musicians |
| What are some of the leading health issues that are depicted in this video? Why might these health issues exist? | Reproductive issues caused by uranium mining; toxicity caused by local mines; potential contamination of drinking water by oil pipelines; depression that may result from not being able to practice traditional ways of life (e.g. facilitator may wish to cite the fact that AIAN youth suicide is an epidemic nationwide in tribal communities); AIAN people being displaced to undesirable lands, and/or their land being used to extract energy sources or water |
| What are some of the ways that this community raises awareness about these issues? | Through activism and organizing; through youth activism, “You’re kind of born into it in my community,” as noted by one youth; through art; through music; through events; through culture and tradition; through blending traditional culture with contemporary art forms |

*We also encourage facilitator to point out in question #1, that the reason for asking for observations about the diversity was to challenge pre-existing perceptions about AIAN people. Facilitator should acknowledge that each participant likely walked in the door with differing levels of knowledge about AIAN people, and that knowledge was likely shaped by what they learned (or didn’t learn) in school, and what they have seen in the media. Facilitator might encourage participants to silently think about those portrayals, and contrast it with what was seen in the video. Facilitator might add that it is important to show a contemporary and real-life depiction of a Native community. Facilitator might also say that as was seen in a poster in the Honor Native Land video, Native people often have to remind everyone that “We are still here.”*

**Slide 11:**

We showed you the prior videos to introduce you to examples that might help illustrate the social and structural determinants of health.

What do the terms “social and structural determinants of health” even mean?

The single most important determinant of one’s health is your zip code. That’s because your zip code is where you eat, live, work, play, and pray. These are the things that make up the social determinants of health, or the “range of personal, social, economic, and environmental factors that contribute to personal and population health.” ^4^ We saw an example of the way that environmental factors can impact the health of individuals and community.

But who/what decides where groups of people live, and whether they will live near uranium mines, for example? The social determinants of health can be influenced by structural racism, also known as the structural determinants of health. That is, “a system in which public policies, institutional practices, cultural representations, and other norms work in various, often reinforcing ways to perpetuate racial group inequity. The tendency of dominant group members and institutions to make decisions or take action that preserves existing **power** hierarchies.” ^5^ In the case of the Navajo nation being located near uranium mines, or Northern California tribes having their waterways and cultural practices of salmon fishing being halted by dams, it was Federal and local policy that determined who got to live where, and what happened on that land. Those in power ultimately dictated the health of those communities.

We also wanted to point out the role of power in cultural representations. Take for example the National Football League who chose to name a football team a derogatory and painful term used to refer to Native Americans. Many health professionals and advocacy organizations have connected the negative effect of the mascot on Native youth mental health, and the epidemic of Native youth suicide.^6,7^ In fact, the California Racial Mascots Act ended the use of the term “Redskin,” but the law at best has had a muted effect, with many schools finding loopholes to keep the associated imagery.^8,9^ This is a clear example of how power (in this case financial and lobbying power), intersects with structural racism and health.

**Slide 12:**

Now we introduce two new terms, Indigenous and colonization. According to the United Nations, “Indigenous peoples are inheritors and practitioners of unique cultures and ways of relating to people and the environment. They have retained social, cultural, economic and political characteristics that are distinct from those of the dominant societies in which they live. Despite their cultural differences, indigenous peoples from around the world share common problems related to the protection of their rights as distinct peoples.” ^10^ This is in contrast to AIAN, who are Indigenous people, but have a distinct political relationship with the United States, and whose tribes have political sovereignty.

We believe that colonization, “The action or process of settling among and establishing control over the indigenous people of an area; the action of appropriating a place or domain for one's own use” are the very practical and ideological roots of the structural determinants of health we explain above. ^11^ Additionally, colonization can not only refer to the physical act of colonizing a space, but can also refer to the act of colonizing ways of thinking and being. One concrete example could be how many AIAN nations view gender and sexuality. Traditionally, many of AIAN societies recognized several genders and had different ideas of sexuality. These ‘Two Spirit’ relatives played (and still do play) a very special spiritual role.^12^ Yet, due to colonization, some of our Two Spirit relatives no longer recognize the special power that they hold according to our traditional beliefs. Many of them have higher rates of depression, suicide, and are subject to the very violence perpetrated on the LGBTQ2S community as a whole. Just imagine what it would look like if we ‘decolonized’ and ‘re-Indigenized’ on this concept gender and sexuality alone.

**Slide 13:**

According to the County Health Rankings Model the clinical care we provide only accounts for 20% of our patients’ length and quality of life.^13^ The social and economic factors, and physical environment that we’ve discussed thus far account for at least 50% of our patients’ health and well-being.

**Slide 14:**

*For the storytelling exercise the facilitator will be asked to prepare the following materials:*

1. *Print out the individual stories from slides 18-20, 22, and 25.*
2. *Obtain blank colored note cards that coincide with the colors on slides 15, 19, 21, and 22 (pink, blue, green, and white index cards). At minimum you should have 5 index cards of each color.*

*This is an experiential exercise, several participants will be asked to participate by reading short stories and following other instructions provided by the facilitator.*

*Step 1 (optional): Ask participants to make a circle around the room.*

*Step 2: Randomly pass out the six stories to six different participants. Ask them to keep the cards face down.*

*Step 3: Randomly pass out pink, blue, green, and white index cards to the other participants.*

**Slide 15:**

Now we are going to pause and use an interactive example to describe some very specific structural determinants of health. The reason we are using storytelling as an example, is to provide an experiential learning opportunity, and illustrate the interconnectedness of how stories connect us to the past, present, and future. This activity also supports various learning styles, including auditory learning. Please take note of the following:

1. What were some of the positive outcomes from this story?
2. What were some of the negative outcomes?
3. Take note of the dates and ages in this story
4. Can you identify and summarize some of the major policies and practices that may have contributed to these outcomes?

Six (6) of you have been provided with a short story with a number and a name on it. You are that person and will be asked to read your story aloud when called upon. Others have been provided with a colored index card. Please hold onto that card and wait for further instructions.

**Slide 16:**

Jenny, can you please read your story out loud. (*follow the slide deck*)

**Slide 17:**

Can the people with blue cards please raise your cards. You are the relatives that attended boarding schools. As a result, you have higher rates of substance abuse, physical, and psychological disparities. You are less connected to your traditional culture. You didn’t teach your children to speak Navajo. Your way of disciplining your children was to hit them. –This is an example of the policy Assimilation-

**Slide 18:**

Jose, can you please read your story out loud.

**Slide 19:**

Can the person with card number three please read their story out loud.

**Slide 20:**

Emma, can you please read your story out loud.

**Slide 21:**

Can the people with pink cards please raise your cards. You are the relatives of Jenny who did not attend boarding schools. You can fluently speak your language, and have successfully passed down ceremonies, stories, and traditions to your children and grandchildren. You shared restorative healing practices with your children and did not use violence to teach them. - This is an example of the policy Self-determination-

**Slide 22:**

Alex, can you please read your story out loud. (*follow the slide deck*)

**Slide 23:**

Can the people with green cards please raise your cards. You are an enrolled member of the Navajo Nation. You also have your Certificate of Indian Blood (CIB) card to prove it. Although you now live in the city, you can go to any Urban Indian Health Organization to access health care. – This is an example of the policies that resulted in the idea and practice of Federal Recognition-

**Slide 24:**

Can the people with white cards please raise your cards. You are the direct descendents of Jenny and Jose. You are one of their children or grandchildren. You grew up in Los Angeles and learned only bits and pieces of your heritage, some of you have never visited your family on the Navajo reservation, while others of you have made strong connections with your extended family. - This is an example of the policy Relocation-

**Slide 25:**

Mariel, can you please read your story out loud. (*follow the slide deck*)

**Slide 26:**

**Special note to the facilitator: If the facilitator notices obvious discomfort or emotional reactions from participants, we invite the facilitator to guide the group through any one of the relaxation techniques such as deep breathing, for example, found at:* [*https://www.helpguide.org/articles/stress/relaxation-techniques-for-stress-relief.htm*](https://www.helpguide.org/articles/stress/relaxation-techniques-for-stress-relief.htm)*. If participants are still noticeably uncomfortable, participants may be referred to Indigenous Circle of Wellness as a resource. Indigenous Circle of Wellness is a mental wellness private practice located in Los Angeles, CA. They offer various services, including mental wellness support, consulting services, and resources. Feel free to contact them by visiting their website at* [*www.icowellness.com*](http://www.icowellness.com)*.*

*At the end of the exercise, the group will then be prompted to answer the questions above.*

*Facilitator may wish to refer to potential discussion points/answers that are displayed below:*

| **Discussion Questions** | **Potential Discussion Points** |
| --- | --- |
| What were some of the positive outcomes from this story? | Mariel, the granddaughter, got an education, and was still interested in her Diné roots; Mariel also made it a point to teach about Indigenous poets and writers in her classroom; Emma learned her traditional ancestral teachings and remained healthy psychologically, spiritually, physically, emotionally; Jenny’s ability to recognize the connection of her trauma to her experiences in boarding school; Relatives who maintained cultural connections tended to have better outcomes |
| What were some of the negative outcomes? | Disconnection from cultural teachings tended to result in negative outcomes; Negative learned behaviors from boarding school were passed down through the generations; Alex died a premature death; policies directly resulted in negative experiences |
| Take note of the dates and ages in this story | Relocation was as recent as 1956; It wasn’t until 1975 that tribes had the ability to educate, house, and provide health care to their own tribal members; there seemed to be a correlation between connection to culture and life expectancy: for example, Emma was connected to her culture and lived to age 89; Alex was relatively disconnected from his culture and experienced the consequences of intergenerational trauma—this may have contributed to his passing away at age 52; young children seemed to experience trauma early on |
| Can you identify and summarize some of the major policies and practices that may have contributed to these outcomes? | **Assimilation:** Boarding Schools were a tool used in the Assimilation Era of Federal Indian policymaking, also known as “Kill the Indian, Save the Man.” They were oftentimes physically, mentally, emotionally, spiritually, and culturally abusive.  **Relocation:** an act that encouraged American Indians to leave reservations and gain vocational skills in major cities such as Los Angeles, Chicago, New York, etc. The promises of relocation oftentimes did not result in the intended outcomes.  **Federal Recognition:** Tribes that have a nation-to-nation relationship with the Federal government are ‘Federally recognized’. Tribal members are often officially ‘enrolled.’ Most tribes have tribal identification cards. This status effectively gives access to certain resources such as health care through the Indian Health Service, or other resources offered through the Bureau of Indian Affairs (BIA), etc. This is in contrast to the ‘Certificate of degree of Indian blood’ (CIDB) referred to in the story. With adequate proof of ancestry the BIA will issue a “certificate of degree of Indian blood” that does not mean one is enrolled in a tribe, but does give the individual access to similar resources.  **Self-Determination:** allowed tribes to exercise sovereignty over their own affairs by allowing them to contract with the Federal government in areas such as education, healthcare, and housing. |

**Slide 27:**

*Transitional slide.*

You just had an interactive experience that underscored some key policies that affected the health and well-being of a family. For those of us who are more linear thinkers and visual learners, we’ll quickly reiterate some of those key policies. This slide is not meant to be memorized!

**Slide 28:**

This is an abbreviated timeline of Federal Indian policies that can be tied to health. We’ll highlight just a few key policies here^14-17^:

1832 and 1849: The first appropriation for AIAN healthcare was in the form of smallpox vaccinations (due to smallpox decimating tribal nations). Of note, the first 15 years of AIAN health was managed by the Department of War, then transferred the Department of the Interior.

Allotment: After the reservation era, reservation land was ‘alloted’ to individual Indians to farm or graze and would be held in trust for 25 years, at which point the land would be ‘patented’ to the individual for a fee. This act resulted in only about half of the land being held by Indians, while the other half was sold to non-Indians.

Assimilation: The aggressive attempt to “Kill the Indian, Save the Man” whereby boarding schools served as one mechanism of assimilating Indians to dominant culture. Children were forcibly removed from their homes, physically, sexually, culturally, and spiritually abused and neglected. This not only resulted in a tremendous loss of traditional family structures, language, culture, and spirituality, but began the cycle of intergenerational trauma where these learned behaviors would be transmitted through generations.

Termination: This was also a tool used to assimilate AIAN whereby tribes’ legal existence was terminated, and they were no longer considered ‘wards’ of the U.S. The termination era would deem more than 100 tribes ‘civilized’ and nullify their recognition by the government. This effectively opened up tribal lands to economic exploitation since their respective reservations were eliminated under this policy.

Relocation: A law intended to encourage Native Americans in the United States to leave Indian reservations, acquire vocational skills, and assimilate into the general population. AIAN were relocated to major cities such as Chicago, San Francisco, Los Angeles, Denver, etc. It’s also known for not living up to the promises made. This policy is largely responsible for the more than 71% of AIAN who live in urban areas in the present day.

Think about how the following slides about AIAN in Los Angeles might relate to some of these policies.

**Slide 29:**

*Facilitator may read the table as is.*

Note that a CHSDA is an Indian Health Service Contract Health Service Delivery Area. These are the geographic locations where most specialty care contracted by the IHS occurs. Thus, CHSDA’s are usually closer to reservation areas, but can still be in urban areas.

**Slide 30:**

*Facilitator may read the table as is.*

**Slide 31:**

*Facilitator may wish to demonstrate the real-time functionality of the Urban Indian Health Institute Data Dashboard website,* [*http://www.uihi.org/urban-indian-health/data-dashboard/*](http://www.uihi.org/urban-indian-health/data-dashboard/)

**Slide 32:**

*Transitional slide*

**Slide 33:**

71% of AIAN now live in urban areas

What policy may have contributed to these demographics?

*Answer*: Relocation

California has the greatest share of the population who identifies as AIAN alone or combination with other races (14%, or 723,000)^18^

Los Angeles County has the largest population of AIAN in the country at 156,000 (according to the US Census American Fact Finder tool, 5 year ACS estimate)^19^

**Slide 34:**

More than one-third of AIAN children in Los Angeles live below the Federal Poverty Level (FPL).

About one in five AIAN families in Los Angeles live below the FPL.

Some of these disparities are more than 3x that of NHW families.^20^

**Slide 35:**

About 45% of AIAN in Los Angeles have at least some college or a college degree^20^

**Slide 36:**

Although AIAN are eligible for public insurance programs such as Medicaid and Medicare, and can access the Indian Health Service, or private insurance, one-quarter of AIAN in Los Angeles are uninsured.^20^ This is higher than NHW, and higher than the rate of un-insurance for Latinos in LA (who often face challenges such as documentation to be eligible for public insurance).^21^

Why do you think this might this be the case?

*Answer:* One possible answer is that there is a large degree of mistrust among AIAN communities of government run programs due to a legacy of abuse and broken promises.^22,23^

**Slide 37:**

This table compares AIAN (in red) to the overall population (in blue) in Los Angeles on various health indicators. The red column on the far right depicts AIAN women specifically.

We mentioned earlier that intergenerational trauma and disconnection from culture resulted in negative outcomes. One tangible outcome is depicted in the 40% of AIAN women who report suicidal ideation, compared to only 7% of the overall population

n in Los Angeles.

*Facilitator, please feel free to highlight other disparities at your choosing.*

**Slide 38:**
Transitional slide.

**Slide 39:**

Facilitator: Read slide verbatim

**Slide 40:**

However, this trust obligation is not being fulfilled as demonstrated by the inequitable per capita spending rates of the various Department of Health and Human Services agencies. For instance, Medicare spends almost $13,000 per capita, while the VA spends roughly $9,000 per capita, and Medicaid spends about $7,800 per capita. IHS spends a fraction of that of other agencies at about $3300 per capita. It’s calculated that IHS is funded only at 60% of need.^24^

**Slide 41:**

Despite comprising 71% of the AIAN population, urban Indian health care only receives 1% of the entire IHS budget.^25^

While LA County has the largest population of AIAN in the country, there is only one small urban Indian Health Organization to serve this population.

**Slide 42:**

To our knowledge, the concept of AIAN having to provide proof of identity originated between 1887-1906. This idea came from the Dawes Act of 1887, which resulted in the creation of the Dawes Rolls. The “Dawes Rolls” were lists of individuals who were accepted as eligible for tribal membership in the "Five Civilized Tribes": Cherokees, Creeks, Choctaws, Chickasaws, and Seminoles. These tribal members were entitled to an allotment of land, in return for abolishing their tribal governments. The rolls listed their name, sex, blood degree, and census card number.^26^ After this era, the Federal government mandated that Federally recognized tribes determine enrollment criteria and suggested that blood quantum be used as the criteria. While tribes traditionally took in non-Indians as tribal members, the messy and difficult reality is that now tribes as sovereign nations must still determine their own criteria for citizenship, which frequently includes blood degree or ‘blood quantum, or some ’proof of descendency’ such as the Dawes Rolls. One unintended consequence might be the following: Say your parents came from 2 different tribal nations each. While you may be ‘full-blooded,’ your blood quantum may not be high enough to qualify you for citizenship in any one of the respective tribal nations. The mathematic reality, is that blood quantum will eventually dwindle down.

Earlier we referred to the Termination Era of policy making when >100 tribes status was terminated. In many cases, members of these tribes have difficulty providing ‘proof’ of descendency. In California, roughly 45 tribes were terminated during this time. In Los Angeles, for instance, while tribal groups include the Tongva, Chumash, and Fernandeno Tataviam, unfortunately none of them have regained their ‘Federal recognition’ status, they do not have reservation land, nor access to other benefits accessible to Federally Recognized tribes.^27^

State-recognized tribes: Tribes have a status within the state but does not guarantee funding from the state or the federal government. State-recognized tribes are not federally recognized; however, federally recognized tribes may also be state-recognized.

Federal Recognition: Tribes that have a nation-to-nation relationship with the Federal government are ‘Federally recognized’. Tribal members are often officially ‘enrolled.’ Most tribes have tribal identification cards. This status effectively gives access to certain resources such as health care through the Indian Health Service, or other resources offered through the Bureau of Indian Affairs (BIA), Bureau of Indian Education, etc.

*If learners are interested in further reading on this issue, we suggest the following article:* *Haozous EA, Strickland CJ, Palacios JF, Arambula-Solomon TG. Blood Politics, Ethnic Identity, and Racial Misclassification among American Indians and Alaska Natives. J Environ Public Health. 2014;2014:321604.*

**Slide 43:**

What does AIAN identity mean in terms of accessing health care? The short answer is it’s complicated. AIAN can access the health system just like any other U.S. Citizen. In addition, IHS is a Federal trust obligation to AIAN people, but less than half are eligible (2.2 million our of roughly 6 million). IHS is not insurance (think of it more like the VA), and it cannot provide minimum essential health benefits as mandated by the Affordable Care Act.

On the insurance side, AIAN from Federally recognized tribes are exempt from the individual mandate.

**Slide 44:**

*Transitional slide. Please read title.*

**Slides 45:**

For those of you who may want a practical clinical tip, consider asking the following questions to all of your patients in your history and physical:

1. What culture do you identify with and what does it mean to you?
2. How does your culture impact your physical, mental, spiritual, or emotional health?

**Slides 46-47:**

Please read verbatim

**Slide 48:**

Q&A

References

1. Urban Indian Health Institute. Data Dashboard. Urban Indian Health Institute website. <http://www.uihi.org/urban-indian-health/data-dashboard/>. Accessed December 14, 2018.
2. Image and video by the United States Department of Arts and Culture (USDAC), retrieved from: https://usdac.us/nativeland/ on November 15, 2018. Permission received from USDAC.
3. Image by Native Land retrieved from: https://native-land.ca/ on November 15, 2018. Permission received from <https://native-land.ca/>.
4. Office of Disease Prevention and Health Promotion. Healthy People 2020: Determinants of Health. <https://www.healthypeople.gov/2020/about/foundation-health-measures/Determinants-of-Health>. Updated December 12, 2018. Accessed December 12, 2018.
5. Thomas SB, Quinn SC, Butler J, Fryer CS, Garza MA. Toward a Fourth Generation of Disparities Research to Achieve Health Equity. Annu Rev Public Health. 2011 ; 32: 399–416.
6. American Psychological Association. APA Resolution Recommending the Immediate Retirement of American Indian Mascots, Symbols, Images, and Personalities by Schools, Colleges, Universities, Athletic Teams, and Organizations. https://www.apa.org/pi/oema/resources/indian-mascots.aspx. Accessed December 11, 2018.
7. National Congress of American Indians. Ending the Era of Harmful “Indian” Mascots campaign. http://www.ncai.org/proudtobe. Accessed December 11, 2018.
8. California Legislative Information. AB-30 School or athletic team names: California Racial Mascots Act. <https://leginfo.legislature.ca.gov/faces/billTextClient.xhtml?bill_id=201520160AB30>. Accessed December 14, 2018.
9. Wild A. Two years later, effect of California Racial Mascots Act looks diminished. The Daily Californian. <http://www.dailycal.org/2017/10/09/two-years-later-effect-california-racial-mascots-act-looks-diminished/>. Published October 2017. Accessed December 14, 2018.
10. United Nations website. https://www.un.org/development/desa/indigenouspeoples/about-us.html. Accessed December 18, 2018.
11. Oxford Dictionary website. https://en.oxforddictionaries.com/definition/colonization. Accessed December 12, 2018.
12. Indian Health Service. Two Spirit. Indian Health Service website. https://www.ihs.gov/lgbt/health/twospirit/. Accessed December 12, 2018.
13. County Health Rankings and Roadmaps. County Health Rankings Model. http://www.countyhealthrankings.org/county-health-rankings-model. Updated March 29, 2016. Accessed December 12, 2018.
14. Shelton BL. Legal and Historical Roots of Health Care for American Indians and Alaska Natives in the United States. The Henry J. Kaiser Foundation, 2004.
15. Indian Resources Timeline. The United States Department of Justice website. https://www.justice.gov/enrd/timeline/indian-resources-timeline. Updated May 2015. Accessed November 19, 2018.
16. Urban Indian Health Commission. Invisible Tribes: Urban Indians and Their Health in a Changing World. Seattle: Urban Indian Health Commission, 2007.
17. Warne D, Frizzell LB. “American Indian Health Policy: Historical Trends and Contemporary Issues,” American Journal of Public Health, vol. 104, no. S3 (June 2014): S263–S267.
18. Norris T, Vines PL, Hoeffl EM. United States Census Bureau. The American Indian and Alaska Native Population: 2010. 2010 Census Briefs. <https://www.census.gov/history/pdf/c2010br-10.pdf>. Issued January 2012. Accessed November 12, 2018.
19. Annual Estimates of the Resident Population by Sex, Single Year of Age, Race, and Hispanic Origin for the United States: April 1, 2010 to July 1, 2016. Source: U.S. Census Bureau, Population Division. Release Date: June 2017. Accessed September 8, 2018.
20. Urban Indian Health Institute, Seattle Indian Health Board. (2017). Community Health Profile: Individual Site Report, Los Angeles Urban Indian Health Program Service Area. Seattle, WA: Urban Indian Health Institute.
21. Los Angeles County Department of Public Health, Office of Women’s Health. Health Indicators for Women in Los Angeles County: Highlighting Disparities by Ethnicity and Poverty Level, January 2017.
22. CM Pacheco, SM Daley, T Brown, M Filippi, KA Greiner, MD, and CM Daley, “Moving Forward: Breaking the Cycle of Mistrust Between American Indians and Researchers,” American Journal of Public Health 103 (2013): 2152–2159.
23. VM Mays, “The Legacy of the U.S. Public Health Service Study of Untreated Syphilis in African American Men at Tuskegee on the Affordable Care Act and Health Care Reform Fifteen Years after President Clinton’s Apology,” Ethics & Behavior vol. 22, no. 6 (2012): 411–418.
24. National Indian Health Board. The National Tribal Budget Formulation Workgroup’s Recommendations on the Indian Health Service Fiscal Year 2020 Budget. “Partnering to Build a Strong and Sustainable Indian Health System: Honoring Tribal Sovereignty to Fulfill the Federal Trust Responsibility.” Released April 2018. Accessed December 13, 2018.
25. Artiga S, Arguello R, Duckett P. Health care coverage for American Indians and Alaska Natives. Menlo Park, CA: Henry J. Kaiser Family Foundation; 2013. Available at https://www.kff.org/disparities-policy/issue-brief/health-coverage-and-care-for-american-indians-and-alaska-natives/view/print/. Accessed October 18, 2018.
26. National Archives. Native American Heritage. Dawes Rolls website. <https://www.archives.gov/research/native-americans/dawes/tutorial/intro.html>. Last updated October 4, 2016. Accessed November 14, 2018.
27. National Conference of State Legislatures. Federal and State Recognized Tribes. <http://www.ncsl.org/research/state-tribal-institute/list-of-federal-and-state-recognized-tribes.aspx>. Accessed December 14, 2018.
