# Supplementary material for: Social and Structural Determinants of Urban American Indian and Alaska Native Health: A Case Study in Los Angeles
Source: MedEdPORTAL. 2019 May 15;15:10825. doi: 10.15766/mep_2374-8265.10825 (PMC6543927; doi:10.15766/mep_2374-8265.10825)
Supplement: Supplementary file 1 — A. PowerPoint Presentation.pptx B. Facilitator Guide.docx C. Video Honor Native Land.mp4 D. Video The Art of Indigenous Resistance.mp4 E. Evaluation Form.pdf [file mep-15-10825-s001.zip › E. Evaluation Form.pdf]

PRE - Workshop Survey

1. A land acknowledgement (circle one response)
   a. Can only be performed by someone of American Indian/Alaskan Native identity
   b. Recognizes the impact that climate change is having on the land
   c. Is to acknowledge the original caretakers of the land
   d. All of the above

2. Which American Indian/Alaska Native tribe identifies the land that this institution resides on as their original homelands (circle one response)?
   a. Cherokee
   b. Mohawk
   c. Lenape
   d. All of the above

3. The purpose of the Relocation Act was to do the following (circle one response)
   a. To forcibly move American Indian/Alaska Natives tribes to reservation lands
   b. To encourage American Indian/Alaska Natives to gain vocational skills and move to urban areas
   c. To relocate American Indian/Alaska Natives from their land to make space for the U.S. agricultural industry
   d. All of the above

4. Who is eligible for healthcare through the Indian Health Service?
   a. Self-identified American Indian/Alaska Natives individuals
   b. Federally recognized American Indian/Alaska Natives with proof of tribal enrollment
   c. Any individual residing on a reservation
   d. All of the above

5. In which **STATE** is your current academic medicine center (e.g. medical school, residency, etc.) located? ______________________

6. Are you a (circle one):
   a. Medical Student
   b. Intern/Resident
   c. Fellow
   d. Faculty
   e. Staff
   f. Other (please specify):__________________________________

7. What is your race/ethnicity (**circle all that apply)?**
   a. American Indian or Alaska Native
   b. Native Hawaiian or Other Pacific Islander
   c. Asian
   d. Black or African-American
   e. Hispanic or Latino
   f. White
   g. Other (please specify):

8. How do you self-identify (**circle one**)?
   a. Straight or Heterosexual
   b. Gay or Lesbian
   c. Bisexual
   d. Other (please specify): ____________________

9. How do you **self-identify**? Note: Respondents who self-identify as "Transgender female-to-male," "Transgender male-to-female," or "Transgender do not identify as exclusively male or female" are combined and displayed as "Transgender" (circle all that apply):
   a. Male
   b. Female
   c. Transgender
   d. Other: ______________________

POST - Workshop Survey					
1. A land acknowledgement (circle one response)<br>e. Can only be performed by someone of American Indian/Alaskan Native (AI/AN) identity<br>f. Recognizes the impact that climate change is having on the land<br>g. Is to acknowledge the original caretakers of the land<br>h. All of the above					
2. Which American Indian/Alaska Native tribe identifies the land that this institution resides on as their original homelands (circle one response)?<br>e. Cherokee<br>f. Mohawk<br>g. Lenape<br>h. All of the above					
3. The purpose of the Relocation Act was to do the following (circle one response)<br>e. To forcibly move American Indian/Alaska Natives tribes to reservation lands<br>f. To encourage American Indian/Alaska Natives to gain vocational skills and move to urban areas<br>g. To relocate American Indian/Alaska Natives from their land to make space for the U.S. agricultural industry<br>h. All of the above					
4. Who is eligible for healthcare through the Indian Health Service?<br>e. Self-identified American Indian/Alaska Natives individuals<br>f. Federally recognized American Indian/Alaska Natives with proof of tribal enrollment<br>g. Any individual residing on a reservation<br>h. All of the above					
5. To what extent do you agree that the workshop learning objectives were met					
	Strongly agree	Agree	Neither agree or disagree	Disagree	Strongly disagree
Obj 1: Describe the importance of and how to identify the tribal homelands on which you stand	SA	A	N	D	SD
Obj 2: Describe how at least two Federal Indian policies that have impacted the demographics and health outcomes of AIAN in urban areas	SA	A	N	D	SD
Obj 3: Explain how AI/AN identity may be associated with access to health care	SA	A	N	D	SD
6. What did you like about this workshop?					
7. What suggestions do you have to improve this workshop?					
